# Non-Negative Decomposition of Multivariate Information: From Minimum to Blackwell-Specific Information

**DOI:** 10.3390/e26050424

**Published:** 2024-05-15

**Authors:** Tobias Mages, Elli Anastasiadi, Christian Rohner

**Affiliations:** Department of Information Technology, Uppsala University, 752 36 Uppsala, Sweden

**Keywords:** partial information decomposition, redundancy, synergy, information flow analysis, f-information, Rényi-information

## Abstract

Partial information decompositions (PIDs) aim to categorize how a set of source variables provides information about a target variable redundantly, uniquely, or synergetically. The original proposal for such an analysis used a lattice-based approach and gained significant attention. However, finding a suitable underlying decomposition measure is still an open research question at an arbitrary number of discrete random variables. This work proposes a solution with a non-negative PID that satisfies an inclusion–exclusion relation for any f-information measure. The decomposition is constructed from a pointwise perspective of the target variable to take advantage of the equivalence between the Blackwell and zonogon order in this setting. Zonogons are the Neyman–Pearson region for an indicator variable of each target state, and f-information is the expected value of quantifying its boundary. We prove that the proposed decomposition satisfies the desired axioms and guarantees non-negative partial information results. Moreover, we demonstrate how the obtained decomposition can be transformed between different decomposition lattices and that it directly provides a non-negative decomposition of Rényi-information at a transformed inclusion–exclusion relation. Finally, we highlight that the decomposition behaves differently depending on the information measure used and how it can be used for tracing partial information flows through Markov chains.

## 1. Introduction

**Figure 1:**
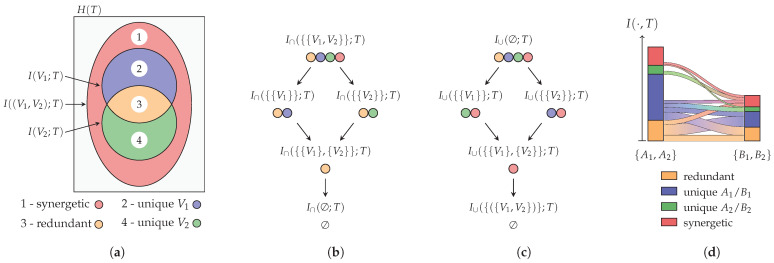
Partial information decomposition representations at two variables $V=\{V_{1},V_{2}\}$. (**a**) Desired set-theoretic analogy: Visualization of the desired intuition for multivariate information as a Venn diagram. (**b**) Representation as redundancy lattice, where the redundancy measure $I_{\cap }$ quantifies the information that is contained in all of its provided variables (inside their intersection). The ordering represents the expected subset relation of redundancy. (**c**) Representation as synergy lattice, where the loss measure $I_{\cup }$ quantifies the information that is contained in neither of its provided variables (outside their union). (**d**) Information flow visualization: When having two partial information decompositions with respect to the same target variable, we can study how the partial information of one decomposition propagates into the next. We refer to this as information flow analysis of a Markov chain such as $T\to \left(A_{1},A_{2}\right)\to \left(B_{1},B_{2}\right)$.

From computer science to neuroscience, we can find the following problem: We would like to know information about a random variable *T*, called the target, which we cannot observe directly. However, we can obtain information about the target indirectly from another set of variables $V=\{V_{1},\ldots ,V_{n}\}$. We can use information measures to quantify how much information any set of variables provides about the target. When doing so, we can identify the concept of *redundancy*: For example, if we have two identical variables $V_{1}=V_{2}$, then we can use one variable to predict the other and, thus, anything that this other variable can predict. Similarly, we can identify the concept of *synergy*: For example, if we have two independent variables and a target that corresponds to their XOR operation $T=(V_{1}\;\mathrm{XOR}\;V_{2})$, then both variables provide no advantage on their own for predicting the state of *T*, yet their combination fully determines it. Williams and Beer [1] suggested that it is possible to characterize information as visualized by the Venn diagram for two variables $V=\{V_{1},V_{2}\}$ in Figure 1a. This decomposition attributes the total information about the target to being redundant, synergetic, or unique to a particular variable. As indicated in Figure 1a by I(·,T), we can quantify three of the areas using information measures. However, this is insufficient to determine the four partial areas that represent the individual contributions. This causes the necessity to extend an information measure to either quantify the amount of redundancy or synergy between a set of variables.

Williams and Beer [1] first proposed a framework for Partial Information Decomposition (PIDs) and found favor by the community [2]. However, the proposed measure of redundancy was criticized for not distinguishing, “the *same* information and the *same amount* of information” [3,4,5,6]. The proposal of Williams and Beer [1] focused specifically on mutual information. This work additionally studies the decomposition of any *f*-information or Rényi-information at discrete random variables. They have significance, among others, in parameter estimations, high-dimensional statistics, hypothesis testing, channel coding, data compression, and privacy analyses [7,8].

### 1.1. Related Work

Most of the literature focuses on the decomposition of mutual information. Here, many alternative measures have been proposed, but cannot fully replace the original measure of Williams and Beer [1] since they do not provide non-negative results for any |V|: The special case of bivariate partial information decompositions (|V|=2) has been well studied, and several non-negative decompositions for the framework of Williams and Beer [1] are known [5,9,10,11,12]. However, each of these decompositions provides negative partial information for |V|>2. Further research [13,14,15] specifically aimed to define decompositions of mutual information for an arbitrary number of observable variables, but similarly obtained negative partial contributions and the resulting difficulty of interpreting their results. Griffith et al. [3] studied the decomposition of zero-error information and obtained negative partial contributions. Kolchinsky [16] proposed a decomposition framework for an arbitrary number of observable variables that is applicable beyond Shannon information theory, however, where the partial contributions do not sum to the total amount.

In this work, we propose a decomposition measure for replacing the one presented by Williams and Beer [1] while maintaining its desired properties. To achieve this, we combine several concepts from the literature: We use the Blackwell order, a preorder of information channels, for the decomposition and for deriving its operational interpretation, similar to Bertschinger et al. [9] and Kolchinsky [16]. We use its special case for binary input channels, the zonogon order studied by Bertschinger and Rauh [17], to achieve non-negativity at an arbitrary number of variables and provide it with a practical meaning by highlighting its equivalence to the Neyman–Pearson (decision) region. To utilize this special case for a general decomposition, we use the concept of a target pointwise decomposition as demonstrated by Williams and Beer [1] and related to Lizier et al. [18], Finn and Lizier [13], and Ince [14]. Specifically, we use Neyman–Pearson regions of an indicator variable for each target state to define distinct information and quantify pointwise information from its boundary. This allows for the non-negative decomposition of an arbitrary number of variables, where the source and target variables can have an arbitrary finite number of states. Finally, we apply the concepts from measuring on lattices, discussed by Knuth [19], to transform a non-negative decomposition with an inclusion–exclusion relation from one information measure to another while maintaining the decomposition properties.

**Remark 1.**  *We use the term “target pointwise” or simply “pointwise” within this work to refer to the analysis of each target state individually. This differs from [13,14,18], who use the latter term for the analysis of all joint source–target realizations.*

### 1.2. Contributions

In a recent work [20], we presented a decomposition of mutual information on the redundancy lattice (Figure 1b). This work aims to simplify, generalize, and extend these ideas to make the following contributions to the area of partial information decompositions:

- We propose a representation of distinct uncertainty and distinct information, which is used to demonstrate the unexpected behavior of the measure by Williams and Beer [1] (Section 2.2 and Section 3.1).
- We propose a non-negative decomposition for any *f*-information measure at an arbitrary number of discrete random variables that satisfies an inclusion–exclusion relation and provides a meaningful operational interpretation (Section 3.2, Section 3.3 and Section 3.5). The decomposition satisfies the original axioms of Williams and Beer [1] (Theorems 3 and 4) and obtains different properties from different information measures (Section 4).
- We demonstrate several transformations of the proposed decomposition: (i) We transform the cumulative measure between different decomposition lattices (Section 3.4). (ii) We demonstrate that the non-negative decomposition of *f*-information directly provides a non-negative decomposition of Rényi- and Bhattacharyya-information at a transformed inclusion–exclusion relation (Section 3.6).

## 2. Background

This section aims to provide the required background information and introduce the notation used. Section 2.1 discusses the Blackwell order and its special case at binary targets, the zonogon order, which will be used for operational interpretations and the representation of *f*-information for its decomposition. Section 2.2 discusses the PID framework of Williams and Beer [1] and the relation between a decomposition based on the redundancy lattice and one based on the synergy lattice. We also demonstrate the unintuitive behavior of the original decomposition measure, which will be resolved by our proposal in Section 3. Section 2.3 provides the considered definitions of *f*-information, Rényi-information, and Bhattacharyya-information for the later demonstration of transforming decomposition results between measures.

**Notation 1**  (Random variables and their distribution). *We use the notation T (upper case) to represent a random variable, ranging over the event space T (calligraphic) containing events t∈T (lower case) and use the notation $P_{T}$ (P with subscript) to indicate its probability distribution. The same convention applies to other variables, such as a random variable S with events s∈S and distribution $P_{S}$. We indicate the outer product of two probability distributions as $P_{S}⊗P_{T}$, which assigns the product of their marginals $P_{S}\left(s\right)\cdot P_{T}\left(t\right)$ to each event (s,t) of the Cartesian product S×T. Unless stated otherwise, we use the notation T, S, and V to represent random variables throughout this work.*

### 2.1. Blackwell and Zonogon Order

**Definition 1**  (Channel). *A channel μ=T→S from T to S represents a garbling of the input variable T, which results in variable S. Within this work, we represent an information channel μ as a (row) stochastic matrix, where each element is non-negative, and all rows sum to one.*

For the context of this work, we consider a variable *S* to be the observation of the output from an information channel T→S from the target variable *T*, such that the corresponding channel can be obtained from their conditional probability distribution, as shown in Equation (1) where $T=\{t_{1},\ldots ,t_{n}\}$ and $S=\{s_{1},\ldots ,s_{m}\}$.

(1) $$\mu =\left(T\to S\right)=P_{\left(S|T\right)}=\left[\begin{array}{ccc} p\left(s_{1}|t_{1}\right) & \cdots & p\left(s_{m}|t_{1}\right)\\ \vdots & \ddots & \vdots \\ p\left(s_{1}|t_{n}\right) & \cdots & p\left(s_{m}|t_{n}\right) \end{array}\right]$$

**Notation 2**  (Binary input channels). *Throughout this work, we reserve the symbol κ for binary input channels, meaning κ signals a stochastic matrix of dimension 2×m. We use the notation $\vec{v}\in \kappa$ to indicate a column of this matrix.*

**Definition 2**  (More informative [17,21]). *An information channel $\mu_{1}=T\to S_{1}$ is more informative than another channel $\mu_{2}=T\to S_{2}$ if—for any decision problem involving a set of actions a∈Ω and a reward function u:(Ω,T)→R that depends on the chosen action and state of the variable T—an agent with access to $S_{1}$ can always achieve an expected reward at least as high as another agent with access to $S_{2}$.*

**Definition 3**  (Blackwell order [17,21]). *The Blackwell order is a preorder of channels. A channel $\mu_{1}$ is Blackwell superior to channel $\mu_{2}$, if we can pass its output through a second channel λ to obtain an equivalent channel to $\mu_{2}$, as shown in Equation (2).*

(2) $$\mu_{2}⊑\mu_{1}⟺\mu_{2}=\mu_{1}\cdot \lambda \;for\;some\;\mathrm{stochastic}\;\mathrm{matrix}\;\lambda$$

Blackwell [21] showed that a channel is more informative if and only if it is Blackwell superior. Bertschinger and Rauh [17] showed that the Blackwell order does not form a lattice for channels μ=T→S if |T|>2 since the ordering does not provide unique meet and join elements. However, binary target variables |T|=2 are a special case where the Blackwell order is equivalent to the zonogon order (discussed next) and does form a lattice [17].

**Definition 4**  (Zonogon [17]). *The zonogon Z(κ) of a binary input channel κ=T→S is defined using the Minkowski sum from the collection of vector segments as shown in Equation (3). The zonogon Z(κ) can similarly be defined as the image of the unit cube ${\left[0,1\right]}^{\left|S\right|}$ under the linear map of κ.*

(3) $$Z\left(\kappa \right)\left\{\sum_{i}x_{i}{\vec{v}}_{i}\;:\;0\leq x_{i}\leq 1,{\vec{v}}_{i}\in \kappa \right\}=\left\{\kappa a\;:\;a\in {\left[0,1\right]}^{\left|S\right|}\right\}$$

**Figure 2:**
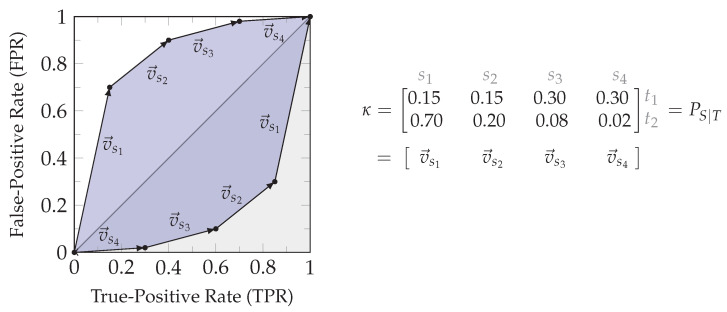
An example zonogon (blue) for a binary input channel κ from $T=\{t_{1},t_{2}\}$ to $S=\{s_{1},s_{2},s_{3},s_{4}\}$. The zonogon is the Neyman–Pearson region, and its perimeter corresponds to the vectors ${\vec{v}}_{s_{i}}\in \kappa$ sorted by an increasing/decreasing slope for the lower/upper half, which results from the likelihood ratio test. The zonogon, thus, represents the achievable (TPR,FPR)-pairs for predicting *T* while knowing *S*.

The zonogon Z(κ) is a centrally symmetric convex polygon, and the set of vectors ${\vec{v}}_{i}\in \kappa$ spans its perimeter. Figure 2 shows an example of a binary input channel and its corresponding zonogon.

**Definition 5**  (Zonogon sum). *The addition of two zonogons corresponds to their Minkowski sum as shown in Equation (4).*

(4) $$Z\left(\kappa_{1}\right)+Z\left(\kappa_{2}\right)\left\{a+b\;:\;a\in Z\left(\kappa_{1}\right),b\in Z\left(\kappa_{2}\right)\right\}=Z\left(\left[\begin{array}{cc} \kappa_{1} & \kappa_{2} \end{array}\right]\right)$$

**Definition 6**  (Zonogon order [17]). *A zonogon $Z(\kappa_{1})$ is zonogon superior to another $Z(\kappa_{2})$ if and only if $Z\left(\kappa_{2}\right)\subseteq Z\left(\kappa_{1}\right)$.*

Bertschinger and Rauh [17] showed that, for binary input channels, the zonogon order is equivalent to the Blackwell order and forms a lattice (Equation (5)). In the remaining work, we will only discuss binary input channels, such that the orderings of Definitions 2, 3, and 6 are equivalent and can be thought of as zonogons with a subset relation.

(5) $$\kappa_{1}⊑\kappa_{2}⟺Z\left(\kappa_{1}\right)\subseteq Z\left(\kappa_{2}\right)$$

To obtain an interpretation of what a channel zonogon Z(κ) represents, we can consider a binary decision problem by aiming to predict the state t∈T of a *binary* target variable *T* using the output of channel κ=T→S. Any decision strategy $\lambda \in {\left[0,1\right]}^{|S|\times 2}$ for obtaining a binary prediction $\hat{T}$ can be fully characterized by its resulting pair of True-Positive Rate (TPR) and False-Positive Rate (FPR), as shown in Equation (6):

(6) $$\kappa \cdot \lambda =\left(T\to S\to \hat{T}\right)=P_{\left(\hat{T}|T\right)}=\left[\begin{array}{cc} p\left(\hat{T}=t|T=t\right) & p\left(\hat{T}\neq t|T=t\right)\\ p\left(\hat{T}=t|T\neq t\right) & p\left(\hat{T}\neq t|T\neq t\right) \end{array}\right]=\left[\begin{array}{cc} \mathrm{TPR} & 1-\mathrm{TPR}\\ \mathrm{FPR} & 1-\mathrm{FPR} \end{array}\right]$$

Therefore, a channel zonogon Z(κ) provides the set of all achievable (TPR,FPR)-pairs for a given channel κ [20,22]. This can also be seen from Equation (3), where the unit cube $a\in {\left[0,1\right]}^{\left|S\right|}$ represents all possible first columns of the decision strategy λ. The first column of λ fully determines the second since each row has to sum to one. As a result, κa provides the (TPR,FPR)-pair for the decision strategy $\lambda =\left[\begin{array}{cc} a\; & \;(1-a) \end{array}\right]$ and the definition of Equation (3) for all achievable (TPR,FPR)-pairs for predicting the state of a binary target variable. Since this will be helpful for operational interpretations, we label the axis of zonogon plots accordingly, as shown in Figure 2. The zonogon ([17], p. 2480) is the Neyman–Pearson region ([7], p. 231).

**Definition 7**  (Neyman–Pearson region [7] and decision regions). *The Neyman–Pearson region for a binary decision problem is the set of achievable (TPR,FPR)-pairs and can be visualized as shown in Figure 2. The Neyman–Pearson regions underlie the zonogon order, and their boundary can be obtained from the likelihood-ratio test. We refer to subsets of the Neyman–Pearson region as reachable decision regions, or simply decision regions, and the boundary as the zonogon perimeter.*

**Remark 2.**  *Due to the zonogon symmetry, the diagram labels can be swapped (FPR x-axis/TPR y-axis), which changes the interpretation to aiming at a prediction for T≠t.*

**Notation 3**  (Channel lattice). *We use the notation $\kappa_{1}⊓\kappa_{2}$ for the meet element of binary input channels under the Blackwell order and $\kappa_{1}⊔\kappa_{2}$ for their join element. We use the notation ${⊤}_{\mathrm{BW}}=\left[\begin{array}{cc} 1\; & \;0\\ 0\; & \;1 \end{array}\right]$ for the top element of binary input channels under the Blackwell order and ${⊥}_{\mathrm{BW}}=\left[\begin{array}{c} 1\\ 1 \end{array}\right]$ for the bottom element.*

For binary input channels, the meet element of the Blackwell order corresponds to the zonogon intersection $Z\left(\kappa_{1}⊓\kappa_{2}\right)=Z\left(\kappa_{1}\right)\cap Z\left(\kappa_{2}\right)$ and the join element of the Blackwell order corresponds to the convex hull of their union $Z\left(\kappa_{1}⊔\kappa_{2}\right)=\mathrm{Conv}\left(Z\left(\kappa_{1}\right)\cup Z\left(\kappa_{2}\right)\right)$. Equation (7) describes this for an arbitrary number of channels.

(7) $$Z\left({⊓}_{\kappa \in A}\kappa \right)=\underset{\kappa \in A}{⋂}Z\left(\kappa \right)\;\mathrm{and}\;Z\left(\underset{\kappa \in A}{⨆}\kappa \right)=Conv\left(\underset{\kappa \in A}{⋃}Z\left(\kappa \right)\right)$$

**Example 1.**  *The remaining work only analyzes indicator variables, so we only need to consider the case |T|=2 where all presented ordering relations of this section are equivalent and form a lattice.*

**Figure 3:**
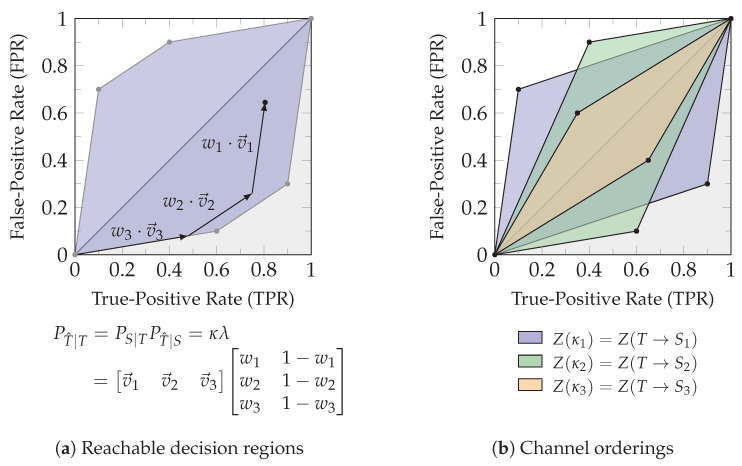
Visualizations for Example 1 where |T|=2. (**a**) A randomized decision strategy for predictions based on $T\overset{\kappa }{\to }S$ can be represented by a |S|×2 stochastic matrix λ. The first column of this decision matrix provides the weights for summing the columns of channel κ to determine the resulting prediction performance (TPR, FPR). Any decision strategy corresponds to a point in the zonogon. (**b**) All presented ordering relations in Section 2.1 are equivalent at binary targets and correspond to the subset relation of the visualized zonogons. The variable $S_{3}$ is less informative than both $S_{1}$ and $S_{2}$ with respect to *T*, and the variables $S_{1}$ and $S_{2}$ are incomparable. The shown channel in (a) is the Blackwell join of $\kappa_{1}$ and $\kappa_{2}$ in (**b**).

*Figure 3a visualizes a channel $T\overset{\kappa }{\to }S$ with |S|=3. We can use the observations of S for making a prediction $\hat{T}$ about T. For example, we predict that T is in its first state with probability $w_{1}$ if S is in its first state, with probability $w_{2}$ if S is in its second state, and with probability $w_{3}$ if S is in its third state. These randomized decision strategies can be noted as stochastic matrix λ shown in Figure 3a. The resulting TPR and FPR of this decision strategy is obtained from the weighted sum of these parameters ($w_{1}$, $w_{2}$, and $w_{3}$) with the vectors in κ. Each decision strategy corresponds to a point within the zonogon, since the probabilities are constrained by $w_{1},w_{2},w_{3}\in \left[0,1\right]$ and the resulting zonogon is the Neyman–Pearson region.*

*Figure 3b visualizes an example for the discussed ordering relations, where all observable variables have two states: $|S_{i}|=2$ where i∈{1,2,3}. The zonogon/Neyman–Pearson region corresponding to variable $S_{3}$ is fully contained within the others ($Z\left(\kappa_{3}\right)\subseteq Z\left(\kappa_{1}\right)$ and $Z\left(\kappa_{3}\right)\subseteq Z\left(\kappa_{2}\right)$). Therefore, we can say that $S_{3}$ is Blackwell inferior (Definition 3) and less informative (Definition 2) than $S_{1}$ and $S_{2}$ about T. Practically, this means that we can construct an equivalent variable to $S_{3}$ by garbling $S_{1}$ or $S_{2}$ and that, for any sequence of actions based on $S_{3}$ and any reward function with dependence on T, we can achieve an expected reward at least as a high by acting based on $S_{1}$ or $S_{2}$ instead. The variables $S_{1}$ and $S_{2}$ are incomparable to the zonogon order, Blackwell order, and informativity order, since the Neyman–Pearson region of one is not fully contained in the other.*

*The zonogon shown in Figure 3a corresponds to the join under the zonogon order, Blackwell order, and informativity order of $S_{1}$ and $S_{2}$ in Figure 3b about T. For binary targets, this distribution can directly be obtained from the convex hull of their Neyman–Pearson regions and corresponds to a valid joint distribution for $\left(T,S_{1},S_{2}\right)$. All other joint distributions are either equivalent or superior to it. When doing this on indicator variables for |T|>2, then the obtained joint distributions for each t∈T may not combine into a specific valid overall joint distribution.*

### 2.2. Partial Information Decomposition

The commonly used framework for PIDs was introduced by Williams and Beer [1]. A PID is computed with respect to a particular random variable that we would like to know information *about*, called the target, and tries to identify *from* which variables that we have access to, called visible variables, we obtain this information. Therefore, this section considers sets of variables that represent their joint distribution.

**Notation 4.**  *Throughout this work, we use the notation T for the target variable and $V=\{V_{1},\ldots ,V_{n}\}$ for the set of visible variables. We use the notation P(V) for the power set of V and $P_{1}\left(V\right)=P\left(V\right)\setminus \emptyset$ for its power set without the empty set.*

**Definition 8**  (Sources, atoms [1]).

- *A source $S_{i}\in P_{1}\left(V\right)$ is a non-empty set of visible variables.*
- *An atom α∈A(V) is a set of sources constructed by Equation (8).*
(8) A(V)={α∈P(P1(V)):∀Sa,Sb∈α,Sa⊂Sb},

The filter used for obtaining the set of atoms (Equation (8)) removes sets that would be equivalent to other elements. This is required for obtaining a lattice from the following two ordering relations:

**Definition 9**  (Redundancy/gain lattice [1]). *The redundancy lattice (A(V),≼) is obtained by applying the ordering relation of Equation (9) to all atoms α,β∈A(V).*

(9) $$\alpha ≼\beta ⟺\forall S_{b}\in \beta ,\exists S_{a}\in \alpha ,S_{a}\subseteq S_{b}$$

**Figure 4:**
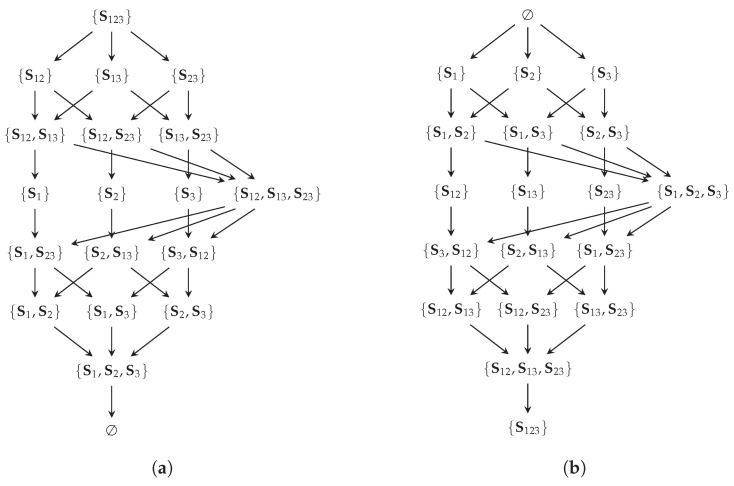
For the visualization, we abbreviated the notation by indicating the contained visible variable as the index of the source, for example $S_{12}=\left\{V_{1},V_{2}\right\}$ to represent their joint distribution: (**a**) A redundancy/gain lattice $\left(A\left(\left\{V_{1},V_{2},V_{3}\right\}\right),\;≼\right)$ based on the ordering of Equation (9) quantifies information present in all sources. The redundancy of all sources within an atom increases while moving up on the redundancy lattice. (**b**) A synergy/loss lattice $\left(A\left(\left\{V_{1},V_{2},V_{3}\right\}\right),\;⪯\right)$ based on the ordering of Equation (10) quantifies information present in neither source. On the synergy lattice, the information that is obtained from neither source of an atom increases while moving up.

The redundancy lattice for three visible variables is visualized in Figure 4a. On this lattice, we can think of an atom as representing the information that can be obtained from all of its sources about the target *T* (their redundancy or informational intersection). For example, the atom $\alpha =\{\left\{V_{1},V_{2}\right\},\left\{V_{1},V_{3}\right\}\}$ represents on the redundancy lattice the information that is contained in both $\left(V_{1},V_{2}\right)$ and $\left(V_{1},V_{3}\right)$ about *T*. Since both sources in α provide the information of $V_{1}$, their redundancy contains at least this information, and the atom $\beta =\{\left\{V_{1}\right\}\}$ is considered its predecessor. Therefore, the ordering indicates an informational subset relation for the redundancy of atoms, and the information that is represented by an atom increases as we move up. The up-set of an atom α on the redundancy lattice indicates the information that is lost when losing all of its sources. Considering the example from above, if we lose access to $\left\{V_{1}\;\left(\mathrm{or}\right)\;V_{2}\right\}$ and $\left\{V_{1}\;\left(\mathrm{or}\right)\;V_{3}\right\}$, then we lose access to all atoms in the up-set of $\alpha =\{\left\{V_{1},V_{2}\right\},\left\{V_{1},V_{3}\right\}\}$.

**Definition 10**  (Synergy/loss lattice [23]). *The synergy lattice (A(V),⪯) is obtained by applying the ordering relation of Equation (10) to all atoms α,β∈A(V).*

(10) $$\alpha ⪯\beta ⟺\forall S_{b}\in \beta ,\exists S_{a}\in \alpha ,S_{b}\subseteq S_{a}$$

The synergy lattice for three visible variables is visualized in Figure 4b. On this lattice, we can think of an atom as representing the information that is contained in neither of its sources (information outside their union). For example, the atom $\alpha =\{\left\{V_{1},V_{2}\right\},\left\{V_{1},V_{3}\right\}\}$ represents on the synergy lattice the information that is obtained from neither $\left(V_{1},V_{2}\right)$ nor $\left(V_{1},V_{3}\right)$ about *T*. The ordering again indicates their expected subset relation: the information that is obtained from neither $\left\{V_{1}\;\left(\mathrm{and}\right)\;V_{2}\right\}$ nor $\left\{V_{1}\;\left(\mathrm{and}\right)\;V_{3}\right\}$ is fully contained in the information that cannot be obtained from $\beta =\{\left\{V_{1}\right\}\}$, and thus, α is a predecessor of β.

With an intuition for both ordering relations in mind, we can see how the filter in the construction of atoms (Equation (8)) removes sets that would be equivalent to another atom: the set $\left\{\left\{V_{1},V_{2}\right\},\left\{V_{1}\right\}\right\}$ is removed from the power set of sources since it would be equivalent to the atom $\left\{\left\{V_{1}\right\}\right\}$ under the ordering of the redundancy lattice and equivalent to the atom $\left\{\left\{V_{1},V_{2}\right\}\right\}$ under the ordering of the synergy lattice. Using Definition 11, one can similarly define the atoms of the decomposition lattices from the power set of sources without the equivalence relation.

**Definition 11.**  *We define equivalence relations for sets of sources under the redundancy and synergy order:*

(11a) $$\begin{array}{cc} \mathrm{Redundancy}\;\mathrm{order}:\;(\alpha ≃\beta ) & ⟺(\alpha ≼\beta \;\;\mathrm{and}\;\;\beta ≼\alpha ) \end{array}$$

(11b) $$\begin{array}{cc} \mathrm{Synergy}\;\mathrm{order}:\;(\alpha ≅\beta ) & ⟺(\alpha ⪯\beta \;\;\mathrm{and}\;\;\beta ⪯\alpha ) \end{array}$$

*We use the notation A{≅}B to indicate that two sets of atoms are equal when comparing their contained atoms with respect to equivalence under the synergy order.*

**Notation 5**  (Redundancy/synergy lattices). *We use the notation (A(V),⋎,⋏) for the join and meet operators on the redundancy lattice, and (A(V),∨,∧) for the join and meet operators on the synergy lattice. We use the notation ${⊤}_{\mathrm{RL}}=\left\{V\right\}$ for the top and ${⊥}_{\mathrm{RL}}=\emptyset$ for the bottom atom on the redundancy lattice, and ${⊤}_{\mathrm{SL}}=\emptyset$ and ${⊥}_{\mathrm{SL}}=\left\{V\right\}$ for the top and bottom atom on the synergy lattice. For an atom α on the redundancy lattice, we use the notation ${↓}_{R}\alpha$ for its down-set, ${\dot{↓}}_{R}\alpha$ for its strict down-set, ${↑}_{R}\alpha$ for its up-set, ${\dot{↑}}_{R}\alpha$ for its strict up-set, and $\alpha^{{-}_{R}}$ for its cover set. For an atom α on the synergy lattice, we use the notation ${↓}_{S}\alpha$ for its down-set, ${\dot{↓}}_{S}\alpha$ for its strict down-set, ${↑}_{S}\alpha$ for its up-set, ${\dot{↑}}_{S}\alpha$ for its strict up-set, and $\alpha^{{-}_{S}}$ for its cover set.*

For convenience, Table 1 provides a summary of the notation used.

The redundant, unique, or synergetic information (partial contributions) can be calculated based on either lattice. They are obtained by quantifying each atom of the redundancy or synergy lattice with a cumulative measure that increases as we move up in the lattice. The partial contributions are then obtained in a second step from a Möbius inverse.

**Definition 12**  ([Cumulative] redundancy measure [1]). *A redundancy measure $I_{\cap }\left(\alpha ;T\right)$ is a function that assigns a real value to each atom of the redundancy lattice. It is interpreted as a cumulative information measure that quantifies the redundancy between all sources S∈α of an atom α∈A(V) about the target T.*

**Definition 13**  ([Cumulative] loss measure [23]). *A loss measure $I_{\cup }\left(\alpha ;T\right)$ is a function that assigns a real value to each atom of the synergy lattice. It is interpreted as a cumulative measure that quantifies the information about T that is provided by neither of the sources S∈α of an atom α∈A(V).*

To ensure that a redundancy measure actually captures the desired concept of redundancy, Williams and Beer [1] defined three axioms that a measure $I_{\cap }$ should satisfy. For the synergy lattice, we consider the equivalent axioms discussed by Chicharro and Panzeri [23]:

**Axiom 1**  (Commutativity [1,23]). *Invariance in the order of sources (σ permuting the order of indices):*

$$\begin{array}{cc} I_{\cap }\left(\left\{S_{1},\ldots ,S_{i}\right\};T\right) & =I_{\cap }\left(\left\{S_{\sigma (1)},\ldots ,S_{\sigma (i)}\right\};T\right)\\ I_{\cup }\left(\left\{S_{1},\ldots ,S_{i}\right\};T\right) & =I_{\cup }\left(\left\{S_{\sigma (1)},\ldots ,S_{\sigma (i)}\right\};T\right) \end{array}$$

**Axiom 2**  (Monotonicity [1,23]). *Additional sources can only decrease redundant information. Additional sources can only decrease the information that is in neither source.*

$$\begin{array}{cc} I_{\cap }\left(\left\{S_{1},\ldots ,S_{i-1}\right\};T\right) & \geq I_{\cap }\left(\left\{S_{1},\ldots ,S_{i}\right\};T\right)\\ I_{\cup }\left(\left\{S_{1},\ldots ,S_{i-1}\right\};T\right) & \geq I_{\cup }\left(\left\{S_{1},\ldots ,S_{i}\right\};T\right) \end{array}$$

**Axiom 3**  (Self-redundancy [1,23]). *For a single source, redundancy equals mutual information. For a single source, the information loss equals the difference between the total available mutual information and the mutual information of the considered source with the target.*

$$\begin{array}{c} I_{\cap }\left(\left\{S_{i}\right\};T\right)=I\left(S_{i};T\right)\;\;\mathrm{and}\;\;I_{\cup }\left(\left\{S_{i}\right\};T\right)=I\left(V;T\right)-I\left(S_{i};T\right) \end{array}$$

The first axiom states that an atom’s redundancy and information loss should not depend on the order of its sources. The second axiom states that adding sources to an atom can only decrease the redundancy of all sources (redundancy lattice) and decrease the information from neither source (synergy lattice). The third axiom binds the measures to be consistent with mutual information and ensures that the bottom element of both lattices is quantified to zero.

Once a lattice with the corresponding cumulative measure ($I_{\cap }$/$I_{\cup }$) is defined, we can use the Möbius inverse to compute the partial contribution of each atom. This partial information can be visualized as the partial area in a Venn diagram (see Figure 1a) and corresponds to the desired redundant, unique, and synergetic contributions. However, the same atom represents different partial contributions on each lattice: As visualized for the case of two visible variables in Figure 1, the unique information of variable $V_{1}$ is represented by $\alpha =\{\left\{V_{1}\right\}\}$ on the redundancy lattice and by $\beta =\{\left\{V_{2}\right\}\}$ on the synergy lattice.

**Definition 14**  (Partial information [1,23]). *Partial information $\Delta I_{\cap }\left(\alpha ;T\right)$ and $\Delta I_{\cup }\left(\alpha ;T\right)$ corresponds to the Möbius inverse of its corresponding cumulative measure on the respective lattice.*

(12a) $$\begin{array}{ccccc} \mathrm{Redundancy}\;\mathrm{lattice}:\; & \; & \Delta I_{\cap }\left(\alpha ;T\right) & =I_{\cap }\left(\alpha ;T\right)-\sum_{\beta \in {\dot{↓}}_{R}\alpha }\Delta I_{\cap }\left(\beta ;T\right), & \end{array}$$

(12b) $$\begin{array}{cccc} \mathrm{Synergy}\;\mathrm{lattice}:\; & \; & \Delta I_{\cup }\left(\alpha ;T\right) & =I_{\cup }\left(\alpha ;T\right)-\sum_{\beta \in {\dot{↓}}_{S}\alpha }\Delta I_{\cup }\left(\beta ;T\right). \end{array}$$

**Remark 3.**  *Using the Möbius inverse for defining partial information enforces an inclusion–exclusion relation in that all partial information contributions have to sum to the corresponding cumulative measure. Kolchinsky [16] argues that an inclusion–exclusion relation should not be expected to hold for PIDs and proposes an alternative decomposition framework. In this case, the sum of partial contributions (unique/redundant/synergetic information) is no longer expected to sum to the total amount I(V;T).*

**Property 1**  (Local positivity, non-negativity [1]). *A partial information decomposition satisfies non-negativity or local positivity if its partial information contributions are always non-negative, as shown in Equation (13).*

(13) $$\forall \alpha \in A\left(V\right).\;\Delta I_{\cap }\left(\alpha ;T\right)\geq 0\;\mathrm{or}\;\Delta I_{\cup }\left(\alpha ;T\right)\geq 0$$

The non-negativity property is important if we assume an inclusion–exclusion relation since it states that the unique, redundant, or synergetic information cannot be negative. If an atom α provides a negative partial contribution in the framework of Williams and Beer [1], then this may indicate that we over-counted some information in its down-set.

**Remark 4.**  *Several additional axioms and properties have been suggested since the original proposal of Williams and Beer [1], such as target monotonicity and the target chain rule [4]. However, this work will only consider the axioms and properties of Williams and Beer [1]. To the best of our knowledge, no other measure since the original proposal (discussed below) has been able to satisfy these properties for an arbitrary number of visible variables while ensuring an inclusion–exclusion relation for their partial contributions.*

It is possible to convert between both representations due to a lattice duality:

**Definition 15**  (Lattice duality and dual-decompositions [23]). *Let $C=(A\left(V\right)\setminus \{{⊥}_{\mathrm{RL}}\},≼)$ be a redundancy lattice with associated measure $I_{\cap }$, and let $D=(A\left(V\right)\setminus \{{⊥}_{\mathrm{SL}}\},⪯)$ be a synergy lattice with measure $I_{\cup }$; then, the two decompositions are said to be dual if and only if the down-set on one lattice corresponds to the up-set in the other, as shown in Equation (14).*

(14a) $$\begin{array}{cc} \forall \alpha \in C,\;\exists \beta \in D & :\Delta I_{\cap }\left(\alpha ;T\right)=\Delta I_{\cup }\left(\beta ;T\right) \end{array}$$

(14b) $$\begin{array}{cc} \forall \alpha \in D,\exists \beta \in C\; & :\Delta I_{\cup }\left(\alpha ;T\right)=\Delta I_{\cap }\left(\beta ;T\right) \end{array}$$

(14c) ∀α∈C,∃β∈D:ΔI∩(α;T)=∑γ∈↓RαΔI∩(γ;T)=∑γ∈↑SβΔI∪(γ;T)

(14d) ∀α∈D,∃β∈C:ΔI∪(α;T)=∑γ∈↓SαΔI∪(γ;T)=∑γ∈↑RβΔI∩(γ;T)

(14e) $$\begin{array}{cc} I_{\cap }\left({⊥}_{\mathrm{RL}};T\right)= & \;I_{\cup }\left({⊥}_{\mathrm{SL}};T\right)=0=\Delta I_{\cap }\left({⊥}_{\mathrm{RL}};T\right)=\Delta I_{\cup }\left({⊥}_{\mathrm{SL}};T\right) \end{array}$$

Williams and Beer [1] proposed $I_{\cap }^{\min}$, as shown in Equation (15), to be used as a measure of redundancy and demonstrated that it satisfies the three required axioms and local positivity. They define redundancy (Equation (15b)) as the expected value of the minimum *specific information* (Equation (15a)).

**Remark 5.**  *Throughout this work, we use the term “target pointwise information” or simply “pointwise information” to refer to “specific information”. This shall avoid confusion when naming their corresponding binary input channels in Section 3.*

(15a) $$\begin{array}{cc} I(S_{i};T=t) & =\sum_{s\in S_{i}}p\left(s|t\right)\left[\log\left(\frac{1}{p(t)}\right)-\log\left(\frac{1}{p(t|s)}\right)\right] \end{array}$$

(15b) $$\begin{array}{cc} I_{\cap }^{\min}\left(S_{1},\ldots ,S_{k};T\right) & =\sum_{t\in T}p\left(t\right)\min_{i\in 1..k}I\left(S_{i};T=t\right). \end{array}$$

To the best of our knowledge, this measure is the only existing non-negative decomposition that satisfies all three axioms listed above for an arbitrary number of visible variables while providing an inclusion–exclusion relation of partial information.

**Figure 5:**
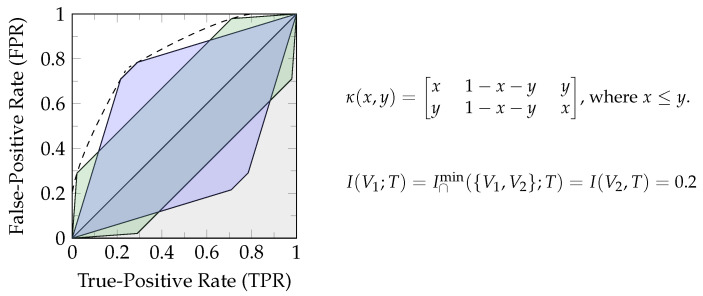
Example of the unexpected behavior of $I_{\cap }^{\min}$: the dashed isoline indicates the pairs (x,y) for which channel $\kappa \left(x,y\right)=T\to V_{i}$ results in pointwise information $\forall t\in T:I(V_{i},T=t)=0.2$ for a uniform binary target variable. Even though observing the output of both indicated example channels (blue/green) provides significantly different abilities for predicting the target variable state, the measure $I_{\cap }^{\min}$ indicates full redundancy.

However, the measure $I_{\cap }^{\min}$ could be criticized for not providing a notion of distinct information due to its use of a pointwise minimum (for each t∈T) over the sources. This leads to the question of distinguishing “the *same* information and the *same amount* of information” [3,4,5,6]. We can use the definition through a pointwise minimum (Equation (15)) to construct examples of unexpected behavior: consider, for example, a uniform binary target variable *T* and two visible variables as the output of the channels visualized in Figure 5. The channels are constructed to be equivalent for both target states and provide access to distinct decision regions while ensuring constant pointwise information $\forall t\in T:I(V_{x},T=t)=0.2$.

Even though our ability to predict the target variable significantly depends on which of the two indicated channel outputs we observe (blue or green in Figure 5, incomparable informativity based on Definition 2), the measure $I_{\cap }^{\min}$ concludes full redundancy between them $I\left(V_{1};T\right)=I_{\cap }^{\min}\left(\left\{V_{1},V_{2}\right\};T\right)=I\left(V_{2},T\right)=0.2$. We think this behavior is undesired and, as discussed in the literature, caused by an underlying lack of distinguishing the *same* information. To resolve this issue, we will present a representation of *f*-information in Section 3.1, which allows the use of all (TPR,FPR)-pairs for each state of the target variable to represent a distinct notion of uncertainty.

### 2.3. Information Measures

This section discusses two generalizations of mutual information at discrete random variables based on *f*-divergences and Rényi-divergences [24,25]. While mutual information has interpretational significance in channel coding and data compression, other *f*-divergences have their significance in parameter estimations, high-dimensional statistics, and hypothesis testing ([7], p. 88), while Rényi-divergences can be found among others in privacy analysis [8]. Finally, we introduce Bhattacharyya information for demonstrating that it is possible to chain decomposition transformations in Section 3.6. All definitions in this section only consider the case of discrete random variables (which is what we need for the context of this work).

**Definition 16**  (*f*-divergence [24]). *Let f:(0,∞)→R be a function that satisfies the following three properties:*

- *f is convex;*
- *f(1)=0;*
- *f(z) is finite for all z>0.*

*By convention, we understand that $f\left(0\right)=\lim_{z\to 0^{+}}f\left(z\right)$ and $0f\left(\frac{0}{0}\right)=0$. For any such function f and two discrete probability distributions P and Q over the event space X, the f-divergence for discrete random variables is defined as shown in Equation (16).*

(16) $$D_{f}\left(P\|Q\right)\sum_{x\in X}Q\left(x\right)f\left(\frac{P(x)}{Q(x)}\right)=E_{Q}\left[f\left(\frac{P(X)}{Q(X)}\right)\right]$$

**Notation 6.**  *Throughout this work, we reserve the name f for functions that satisfy the required properties for an f-divergence of Definition 16.*

An *f*-divergence quantifies a notion of dissimilarity between two probability distributions *P* and *Q*. Key properties of *f*-divergences are their non-negativity, their invariance under bijective transformations, and them satisfying a data-processing inequality ([7], p. 89). A list of commonly used *f*-divergences is shown in Table 2. Notably, the continuation for a=1 of both the Hellinger- and α-divergence results in the KL-divergence [26].

The generator function of an *f*-divergence is not unique since $D_{f(z)}=D_{f(z)+c(z-1)}$ for a real constant c∈R ([7], p. 90f). As a result, the considered α-divergence is a linear scaling of the Hellinger divergence ($D_{H_{a}}=a\cdot D_{\alpha =a}$), as shown in Equation (17).

(17) $$\frac{z^{a}-1}{a-1}+c\left(z-1\right)=a\cdot \frac{z^{a}-1-a\left(z-1\right)}{a(a-1)}\;\;\mathrm{for}\;\;c=-\frac{a}{a-1}$$

**Definition 17**  (*f*-information [7]). *An f-information is defined based on an f-divergence from the joint distribution of two discrete random variables and the product of their marginals, as shown in Equation (18).*

(18) $$\begin{array}{cc} I_{f}(S;T): & =D_{f}\left(P_{\left(S,T\right)}\|P_{S}⊗P_{T}\right)\\ \; & =\sum_{(s,t)\in S\times T}P_{S}\left(s\right)\cdot P_{T}\left(t\right)\cdot f\left(\frac{P_{\left(S,T\right)}\left(s,t\right)}{P_{S}\left(s\right)\cdot P_{T}\left(t\right)}\right)\\ \; & =\sum_{t\in T}P_{T}\left(t\right)\left[\sum_{s\in S}P_{S}\left(s\right)\cdot f\left(\frac{P_{S|T}\left(s|t\right)}{P_{S}\left(s\right)}\right)\right] \end{array}$$

**Definition 18**  (*f*-entropy). *A notion of f-entropy for a discrete random variable is obtained from the self-information of a variable $H_{f}\left(T\right)I_{f}\left(T;T\right)$.*

**Notation 7.**  *Using the KL-divergence results in the definition of mutual information and Shannon entropy. Therefore, we use the notation $I_{\mathrm{KL}}$ for mutual information (KL-information) and $H_{\mathrm{KL}}$ (KL entropy) for the Shannon entropy.*

The remaining part of this section will define Rényi- and Bhattacharyya-information to highlight that they can be represented as an invertible transformation of Hellinger-information. This will be used in Section 3.6 to transform the decomposition of Hellinger-information to a decomposition of Rényi- and Bhattacharyya-information.

**Remark 6.**  *We could similarly choose to represent Rényi-divergence as a transformation of the α-divergence. A liner scaling of the considered f-divergence will, however, not affect our later results (see Section 3.6).*

**Definition 19**  (Rényi divergence [25]). *Let P and Q be two discrete probability distributions over the event space X, then Rényi-divergence $R_{a}$ is defined as shown in Equation (19) for a∈(0,1)∪(1,∞), and extended to a∈{0,1,∞} by continuation.*

(19) $$\begin{array}{cc} R_{a}(P\|Q): & =\frac{1}{a-1}\log\left(E_{Q}\left[{\left(\frac{P(X)}{Q(X)}\right)}^{a}\right]\right)\\ \; & =\frac{1}{a-1}\log\left(1+\left(a-1\right)E_{Q}\left[\frac{{\left(\frac{P(X)}{Q(X)}\right)}^{a}-1}{a-1}\right]\right)\\ \; & =\frac{1}{a-1}\log\left(1+\left(a-1\right)D_{H_{a}}\left(P\|Q\right)\right) \end{array}$$

Notably, the continuation of Rényi-divergence for a=1 also equals the KL-divergence ([7], p. 116). Rényi-divergence can be expressed as an invertible transformation of the Hellinger-divergence ($D_{H_{a}}$; see Equation (19)) [26].

**Definition 20**  (Rényi-information [7]). *Rényi-information is defined equivalent to f-information as shown in Equation (20) and corresponds to an invertible transformation of Hellinger-information ($I_{H_{a}}$).*

(20) $$\begin{array}{cc} I_{R_{a}}\left(S;T\right): & =R_{a}\left(P_{\left(S;T\right)}\|P_{S}⊗P_{T}\right)\\ \; & =\frac{1}{a-1}\log\left(1+\left(a-1\right)I_{H_{a}}\left(S;T\right)\right) \end{array}$$

Finally, we consider the Bhattacharyya distance (Definition 21), which is equivalent to a linear scaling from a special case of Rényi-divergence (Equation (21)) [26]. It is applied, among others, in signal processing [27] and coding theory [28]. The corresponding information measure (Equation (22)) is like its distance, the scaling of a special case of Rényi-information.

**Definition 21**  (Bhattacharyya distance [29]). *Let P and Q be two discrete probability distributions over the event space X, then the Bhattacharyya distance is defined as shown in Equation (21).*

(21) $$\begin{array}{cc} B(P\|Q): & =-\log\left(\sum_{x\in X}\sqrt{P(x)Q(x)}\right)\\ \; & =-\log\left(\sum_{x\in X}Q\left(x\right)\sqrt{\frac{P(x)}{Q(x)}}\right)\\ \; & =-\log\left(1-0.5\cdot E_{Q}\left[\frac{{\left(\frac{P(X)}{Q(X)}\right)}^{0.5}-1}{0.5-1}\right]\right)\\ \; & =-\log\left(1-0.5\cdot D_{H_{0.5}}\left(P\|Q\right)\right)\\ \; & =0.5\cdot R_{0.5}\left(P\|Q\right) \end{array}$$

**Definition 22**  (Bhattacharyya-information). *Bhattacharyya-information is defined equivalent to f-information as shown in Equation (22).*

(22) $$I_{B}\left(S;T\right)B\left(P_{\left(S,T\right)}\|P_{S}⊗P_{T}\right)=0.5\cdot I_{R_{0.5}}\left(S;T\right)$$

**Example 2.**  *Consider the channel $T\overset{\kappa }{\to }S$ with $T=\{t_{1},t_{2}\}$ and $S=\{s_{1},s_{2}\}$. While it will be discussed in more detail in Section 3.1, Equation (23) already indicates that f-information can be interpreted as the expected value of quantifying the boundary of the Neyman–Pearson region for an indicator variable of each target state t∈T. Each state of a source variable s∈S corresponds to one side/edge of this boundary as discussed in Section 2.1 and visualized in Figure 2. Therefore, the sum over s∈S corresponds to the sum of quantifying each edge of the zonogon by some function, which is only parameterized by the distribution of the indicator variable for t. This function satisfies a triangle inequality (Corollary A1), and the total boundary is non-negative (Theorem 2 discussed later). Therefore, we can vaguely think of pointwise f-information as quantifying the length of the boundary of the Neyman–Pearson region or zonogon perimeter to give an oversimplified intuition.*

(23) $$\begin{array}{cc} I_{f}\left(S;T\right) & =\sum_{t\in T}P_{T}\left(t\right)\underset{\mathrm{pointwise}\;\mathrm{information}\;\mathrm{of}\;\mathrm{an}\;\mathrm{indicator}\;\mathrm{variable}\;T=t}{\underset{︸}{\left[\sum_{s\in S}\overset{\overset{\mathrm{quantifies}\;\mathrm{each}\;\mathrm{zonogon}\;\mathrm{edge}}{︷}}{P_{S}\left(s\right)\cdot f\left(\frac{P_{S|T}\left(s|t\right)}{P_{S}\left(s\right)}\right)}\right]}} \end{array}$$

*Below is a stepwise computation of $\chi^{2}$-information ($f\left(z\right)={\left(z-1\right)}^{2}$) on a small example from this interpretation for the setting of Equation (24).*

(24a) $$\begin{array}{cc} \kappa =P_{S|T} & =\left[\begin{array}{cc} p(S=s_{1}|T=t_{1}) & p(S=s_{2}|T=t_{1})\\ p(S=s_{1}|T=t_{2}) & p(S=s_{2}|T=t_{2}) \end{array}\right]=\left[\begin{array}{cc} 0.8 & 0.2\\ 0.35 & 0.65 \end{array}\right] \end{array}$$

(24b) PT=p(T=t1)p(T=t2)=0.40.600

**Figure 6:**
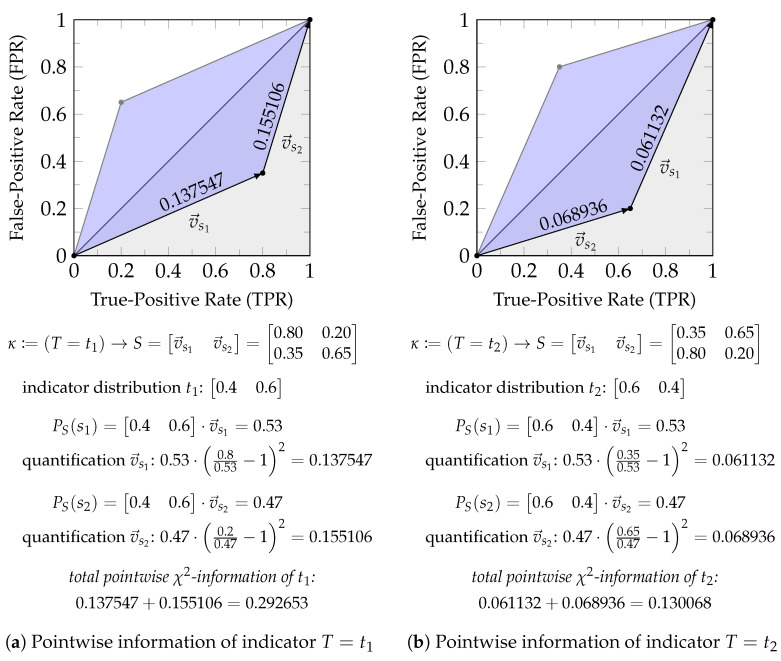
This example visualizes the computation of $\chi^{2}$-information by indicating its results on the representation of zonogons of an indicator variable. (**a**) For the pointwise information of $t_{1}$, both vectors of the zonogon perimeter are quantified to the sum 0.292653. (**b**) For the pointwise information of $t_{2}$, both vectors of the zonogon perimeter are quantified to the sum of 0.130068. The final $\chi^{2}$-information is their expected value $I_{\chi^{2}}\left(S;T\right)=0.4\cdot 0.292653+0.6\cdot 0.130068=0.195102$.

*Since |T|=2, we compute the pointwise information for two indicator variables as shown in Figure 6. Since each state s∈S corresponds to one edge of the zonogon, we compute them individually. Notice that the quantification of each vector $v_{s_{i}}$ can be expressed as a function that is only parameterized by the distribution of the indicator variable. The total zonogon perimeter is quantified as the sum of each of its edges, which equals pointwise information. In this particular case, we obtain 0.292653 for the total boundary on the indicator of $t_{1}$ and 0.130068 for the total boundary on the indicator of $t_{2}$. The expected information corresponds to the expected value of these pointwise quantifications and provides the final result (Equation (25)).*

(25) $$I_{\chi^{2}}\left(S;T\right)=p\left(T=t_{1}\right)\cdot 0.292653+p\left(T=t_{2}\right)\cdot 0.130068=0.195102$$

## 3. Decomposition Methodology

To construct a partial information decomposition in the framework of Williams and Beer [1], we only have to define a cumulative redundancy measure ($I_{\cap }$) or cumulative loss measure ($I_{\cup }$). However, doing this requires a meaningful definition of when information is the *same*. Therefore, Section 3.1 presents an interpretation of *f*-information that enables a representation of distinct information. Specifically, we demonstrate that pointwise *f*-information for a target state t∈T corresponds to the Neyman–Pearson region of its indicator variable, which is quantified by its boundary (zonogon perimeter). This allows for the interpretation that each distinct (TPR,FPR)-pair for predicting a state of the target variable provides a distinct notion of uncertainty. This interpretation of *f*-information is used in Section 3.2 to construct a partial information decomposition on the synergy lattice under the Blackwell order for each state t∈T individually. These individual decompositions are then combined into the final result. Therefore, we decompose specific information based on the Blackwell order rather than using its minimum, like Williams and Beer [1]. The resulting operational interpretation is discussed in Section 3.3. Section 3.4 studies the relation between decomposition lattices to derive the dual-decomposition of any *f*-information on the redundancy lattice in the following Section 3.5 and prove its correctness. We use the obtained decomposition for any *f*-information in Section 3.6 to transform a Hellinger-information decomposition into a Rényi-information decomposition while maintaining its non-negativity and an inclusion–exclusion relation. To achieve the desired axioms and properties, we combine different aspects of the existing literature:

- Like Bertschinger et al. [9] and Kolchinsky [16], we base the decomposition on the Blackwell order and use this to obtain the operational interpretation of the decomposition.
- Like Williams and Beer [1] and related to Lizier et al. [18], Finn and Lizier [13], and Ince [14], we perform a decomposition from a pointwise perspective, but only for the target variable.
- In a similar manner to how Finn and Lizier [13] used probability mass exclusion to differentiate distinct information, we use Neyman–Pearson regions for each state of a target variable to differentiate distinct information.
- We propose applying the concepts about lattice re-graduations discussed by Knuth [19] to PIDs to transform the decomposition of one information measure to another while maintaining its consistency.

We extend Axiom 3 of Williams and Beer [1] as shown below, to allow binding any information measure to the decomposition.

Axiom 3* (Self-redundancy). For a single source, redundancy $I_{\cap ,*}$ and information loss $I_{\cup ,*}$ correspond to information measure $I_{*}$ as shown below:

(26) $$I_{\cap ,*}\left(\left\{S_{i}\right\};T\right)=I_{*}\left(S_{i};T\right)\;\;\mathrm{and}\;\;I_{\cup ,*}\left(\left\{S_{i}\right\};T\right)=I_{*}\left(V;T\right)-I_{*}\left(S_{i};T\right)$$

### 3.1. Representing f-Information

We begin with an interpretation of *f*-information, for which we define a pointwise (indicator) variable π(T,t) that represents one state of the target variable (Equation (27a)) and construct its pointwise information channel (Definition 23). Then, we define a function $r_{f}$ based on the generator function of an *f*-divergence for quantifying (half) the zonogon perimeter of each pointwise information channel (see Figure 2). These perimeter quantifications are pointwise *f*-information.

**Definition 23**  ([Target] pointwise binary input channel). *We define a target pointwise binary input channel κ(S,T,t) from one state of the target variable t∈T to an information source S with event space $S=\{s_{1},\cdots ,s_{m}\}$ as shown in Equation (27b).*

(27a) $$\begin{array}{cc} \pi (T,t) & \left\{\begin{array}{cc} 1 & \mathrm{if}\;\;T=t\\ 0 & \mathrm{otherwise} \end{array}\right. \end{array}$$

(27b) $$\begin{array}{cc} \kappa (S,T,t)\pi (T,t)\to S & =\left[\begin{array}{ccc} p(S=s_{1}|T=t) & \cdots & p(S=s_{m}|T=t)\\ p(S=s_{1}|T\neq t) & \cdots & p(S=s_{m}|T\neq t) \end{array}\right] \end{array}$$

**Definition 24**  ([Target] pointwise *f*-information).

- *We define a function $r_{f}$ as shown in Equation (28a) to quantify a vector, where 0≤p,x,y≤1.*
- *We define a target pointwise f-information function $i_{f}$, as shown in Equation (28b), to quantify half the zonogon perimeter for the corresponding pointwise channel Z(κ(S,T,t)).*

(28a) $$\begin{array}{cc} r_{f}\left(p,\left[\begin{array}{c} x\\ y \end{array}\right]\right): & =\left(px+(1-p)y\right)\cdot f\left(\frac{x}{px+(1-p)y}\right) \end{array}$$

(28b) $$\begin{array}{cc} i_{f}\left(p,\kappa \right): & =\sum_{\vec{v}\in \kappa }r_{f}\left(p,\vec{v}\right) \end{array}$$

**Theorem 1**  (Properties of $r_{f}$). *For a constant 0≤p≤1, (1) the function $r_{f}\left(p,\vec{v}\right)$ is convex in $\vec{v}$, (2) scales linearly in $\vec{v}$, (3) satisfies a triangle inequality in $\vec{v}$, (4) quantifies any vector of slope one to zero, and (5) quantifies the zero vector to zero.*

**Proof.** 

1. The convexity of $r_{f}\left(p,\vec{v}\right)$ in $\vec{v}$ is shown separately in Lemma A1 of Appendix A.
2. That $r_{f}\left(p,\ell \vec{v}\right)=\ell r_{f}\left(p,\vec{v}\right)$ scales linearly in $\vec{v}$ can directly be seen from Equation (28a).
3. The triangle inequality of $r_{f}\left(p,\vec{v}\right)$ in $\vec{v}$ is shown separately in Corollary A1 of Appendix A.
4. A vector of slope one is quantified to zero $r_{f}\left(p,\left[\begin{array}{c} \ell \\ \ell \end{array}\right]\right)=\ell \cdot f\left(1\right)=0$, since $f\left(1\right)=0$ is a requirement on the generator function of an *f*-divergence (Definition 16).
5. The zero vector is quantified to zero $r_{f}\left(p,\left[\begin{array}{c} 0\\ 0 \end{array}\right]\right)=0\cdot f\left(\frac{0}{0}\right)=0$ by the convention of generator functions for an *f*-divergence (Definition 16).

□

The function $r_{f}$ provides the following properties to the pointwise information measure $i_{f}$.

**Theorem 2**  (Properties of $i_{f}$). *The pointwise information measure $i_{f}$ (1) maintains the ordering relation of the Blackwell order for binary input channels and (2) is non-negative.*

**Proof.** 

1. That the function $r_{f}$ maintains the ordering relation of the Blackwell order on binary input channels is shown separately in Lemma A2 of Appendix A (Equation (29a)).
2. The bottom element ${⊥}_{\mathrm{BW}}=\left[\begin{array}{c} 1\\ 1 \end{array}\right]$ consists of a single vector of slope one, which is quantified to zero by Theorem 1 (Equation (29b)). The combination with Equation (29a) ensures the non-negativity.

(29a) $$\begin{array}{cc} \kappa_{1}⊑\kappa_{2}⟹ & \;i_{f}\left(p,\kappa_{1}\right)\leq i_{f}\left(p,\kappa_{2}\right), \end{array}$$

(29b) $$\begin{array}{cc} \; & \;i_{f}\left(p,{⊥}_{\mathrm{BW}}\right)=0. \end{array}$$

□

An *f*-information corresponds to the expected value of the target pointwise *f*-information function defined above (Equation (30)). As a result, we can interpret *f*-information as the expected value of quantifying (half) the zonogon perimeters for the target pointwise channels κ(S,T,t).

(30) $$\begin{array}{cc} I_{f}\left(S;T\right)\; & =\sum_{t\in T}P_{T}\left(t\right)\cdot i_{f}\left(P_{T}\left(t\right),\kappa \left(S,T,t\right)\right)\\ \; & =\sum_{t\in T}P_{T}\left(t\right)\cdot \left[\sum_{\vec{v}\in \kappa \left(S,T,t\right)}r_{f}\left(P_{T}\left(t\right),\vec{v}\right)\right]\\ \; & =\sum_{t\in T}P_{T}\left(t\right)\cdot \left[\sum_{s\in S}P_{S}\left(s\right)\cdot f\left(\frac{P_{S|T}\left(s|t\right)}{P_{S}\left(s\right)}\right)\right] \end{array}$$

### 3.2. Decomposing f-Information on the Synergy Lattice

With the representation of Section 3.1 in mind, we can define a non-negative partial information decomposition for a set of visible variables $V=\{V_{1},\ldots ,V_{n}\}$ about a target variable *T* for any *f*-information. The decomposition is performed from a pointwise perspective, which means that we decompose the pointwise measure $i_{f}$ on the synergy lattice (A(V),⪯) for each t∈T. The pointwise synergy lattices are then combined using a weighted sum to obtain the decomposition of $I_{f}$.

We map each atom of the synergy lattice to the join of pointwise channels for its contained sources.

**Definition 25**  (From atoms to channels). *We define the channel corresponding to an atom α∈A(V) as shown in Equation (31).*

(31) $$\kappa_{⊔}\left(\alpha ,T,t\right)\left\{\begin{array}{cc} {⊥}_{\mathrm{BW}} & \mathrm{if}\;\alpha =\emptyset \\ \underset{S\in \alpha }{⨆}\kappa \left(S,T,t\right)) & \mathrm{otherwise} \end{array}\right.$$

**Lemma 1.**  *For any set of sources $\alpha ,\beta \in P(P_{1}\left(V\right))$ and target variable T with state t∈T, the function $\kappa_{⊔}$ maintains the ordering of the synergy lattice under the Blackwell order as shown in Equation (32).*

(32) $$\alpha ⪯\beta ⟹\kappa_{⊔}\left(\beta ,T,t\right)⊑\kappa_{⊔}\left(\alpha ,T,t\right)$$

Lemma 1 is shown separately in Appendix C. The mapping from Definition 25 provides a lattice that can be quantified using pointwise *f*-information to construct a cumulative loss measure for its decomposition using the Möbius inverse.

**Definition 26**  ([Target] pointwise cumulative and partial loss measures). *We define the target pointwise cumulative and partial loss functions as shown in Equations (33a) and (33b).*

(33a) $$\begin{array}{cc} i_{\cup ,f}\left(\alpha ,T,t\right): & =i_{f}\left(P_{T}\left(t\right),\kappa \left(V,T,t\right)\right)-i_{f}\left(P_{T}\left(t\right),\kappa_{⊔}\left(\alpha ,T,t\right)\right) \end{array}$$

(33b) $$\begin{array}{cc} \Delta i_{\cup ,f}\left(\alpha ,T,t\right): & =i_{\cup ,f}\left(\alpha ,T,t\right)-\sum_{\beta \in {\dot{↓}}_{S}\alpha }\Delta i_{\cup ,f}\left(\beta ,T,t\right) \end{array}$$

The combined cumulative and partial measures are the expected value of their corresponding pointwise measures. This corresponds to combining the pointwise decomposition lattices by a weighted sum.

**Definition 27**  (Combined cumulative and partial loss measures). *The cumulative loss measure $I_{\cup ,f}$ is defined by Equation (34) and the decomposition result $\Delta I_{\cup ,f}$ by Equation (35).*

(34) $$I_{\cup ,f}\left(\alpha ;T\right)\sum_{t\in T}P_{T}\left(t\right)\cdot i_{\cup ,f}\left(\alpha ,T,t\right)$$

(35) $$\begin{array}{cc} \Delta I_{\cup ,f}\left(\alpha ;T\right): & =\sum_{t\in T}P_{T}\left(t\right)\cdot \Delta i_{\cup ,f}\left(\alpha ,T,t\right)\\ \; & =I_{\cup ,f}\left(\alpha ;T\right)-\sum_{\beta \in {\dot{↓}}_{S}\alpha }\Delta I_{\cup ,f}\left(\beta ;T\right) \end{array}$$

**Theorem 3.**  The presented definitions for the pointwise and expected loss measures ($i_{\cup ,f}$ and $I_{\cup ,f}$) provide a non-negative PID on the synergy lattice with an inclusion–exclusion relation that satisfies Axioms 1, 2, and 3* for any f-information measure.

**Proof.** 

- **Axiom 1**: The measure $i_{\cup ,f}$ (Equation (33a)) is invariant to permuting the order of sources in α, since the join operator of the zonogon order (${⨆}_{S\in \alpha }$) is. Therefore, also $I_{\cup ,f}$ satisfies Axiom 1.
- **Axiom 2**: The monotonicity of both $i_{\cup ,f}$ and $I_{\cup ,f}$ on the synergy lattice is shown separately as Corollary A2 in Appendix C.
- Axiom 3*: For a single source, $i_{\cup ,f}$ equals the pointwise information loss by definition (see Equations (26), (28b), and (33a)). Therefore, $I_{\cup ,f}$ satisfies Axiom 3*.
- **Non-negativity**: The non-negativity of $\Delta i_{\cup ,f}$ and $\Delta I_{\cup ,f}$ is shown separately as Lemma A8 in Appendix C.

□

### 3.3. Operational Interpretation

From a pointwise perspective (|T|=2), there always exists a dependency between the sources for which the synergy of this state becomes zero. This dependence corresponds, by definition, to the join of their channels. This is helpful for the operational interpretation in the following paragraph since, individually, each pointwise synergy becomes fully volatile to the dependence between the sources. There may not exist a dependency between the sources for which the expected synergy becomes zero for |T|>2. However, each decision region that is quantified as synergetic becomes inaccessible at some dependence between the sources.

The decomposition obtains the operational interpretation that, if a variable provides pointwise unique information, then there exists a unique decision region for some t∈T that this variable provides access to. Moreover, if a set of variables provides synergetic information, then a decision region for some t∈T may become inaccessible if the dependence between the variables changes. Due to the equivalence of the zonogon and Blackwell order for binary input variables, these interpretations can also be transferred to a set of actions a∈Ω and a *pointwise* reward function u(a,π(T,t)), which only depends on one state of the target variable π(T,t) (see Section 2.1): If a variable provides unique information, then it provides an advantage for some set of actions and pointwise reward function, while synergy indicates that the advantage for some pointwise reward function is based on the dependence between variables.

The implication of the interpretation does not hold in the other direction, which we will also highlight in the example of $I_{\cup ,\mathrm{TV}}$ in Section 4.1. Finally, the definition of the Blackwell order through the chaining of channels (Equation (2)) highlights its suitability for tracing the flows of information in Markov chains (see Section 4.2).

**Remark 7.**  *The operational interpretation can be strengthened further such that the implication between accessible regions and partial information holds in both directions by revising Lemmas A1 and A2 with a strictly convex generator function to obtain $\kappa_{1}⊏\kappa_{2}⟹i_{f}\left(p,\kappa_{1}\right)<i_{f}\left(p,\kappa_{2}\right)$.*

### 3.4. Decomposition Duality

A non-negative decomposition on the synergy lattice raises the question about its dual-decomposition on the redundancy lattice. Unfortunately, the definition of decomposition duality (Definition 15 [23]) does not specify the mapping between atoms to easily construct dual-decompositions. Therefore, this section discusses how the redundancy and synergy lattice are related by identifying operators that transform one lattice into the other. This transformation can then be used to refine the definition of decomposition duality and, correspondingly, transforms the cumulative measure between lattices.

**Definition 28.**  *We define two functions: The function $\Xi :P\left(P_{1}\left(V\right)\right)\to P\left(P_{1}\left(V\right)\right)$ provides the atom with complement sources, and the function $\Psi :P\left(P_{1}\left(V\right)\right)\to P\left(P_{1}\left(V\right)\right)$ is the n-ary Cartesian product. We indicate the i-th source of an atom as α[i] and indicate some variable within the i-th source as $x_{i}$.*

(36) $$\begin{array}{cc} \Xi (\alpha ) & \left\{\begin{array}{cc} \emptyset & \mathrm{if}\;\alpha =\{V\}\\ \left\{V\right\} & \mathrm{if}\;\alpha =\emptyset \\ \left\{V\setminus \{S\}\;:\;S\in \alpha \right\}\; & \mathrm{otherwise} \end{array}\right.\\ \Psi (\alpha ) & \left\{\begin{array}{cc} \left\{\emptyset \right\} & \mathrm{if}\;\alpha =\emptyset \\ \left\{\left\{x_{1},\cdots ,x_{m}\right\}\;:\;x_{1}\in \alpha \left[1\right],\cdots ,x_{m}\in \alpha \left[m\right]\right\}\; & \mathrm{otherwise},\;\mathrm{where}\;m=|\alpha | \end{array}\right. \end{array}$$

**Example 3.**  *For an example of these functions, let $V=\{V_{1},V_{2},V_{3},V_{4}\}$ and $\alpha =\{\left\{V_{1}\right\},\left\{V_{2},V_{3}\right\}\}$:*

$$\begin{array}{ccc} \Xi (\alpha ) & =\{\left\{\left\{V_{2},V_{3},V_{4}\right\},\left\{V_{1},V_{4}\right\}\right\}\}\\ \Xi (\Xi (\alpha )) & =\{\left\{V_{1}\right\},\left\{V_{2},V_{3}\right\}\}=\alpha \\ \Psi (\alpha ) & =\{\left\{V_{1},V_{2}\right\},\left\{V_{1},V_{3}\right\}\}\\ \Psi (\Psi (\alpha )) & =\{\left\{V_{1}\right\},\left\{V_{1},V_{3}\right\},\left\{V_{2},V_{1}\right\},\left\{V_{2},V_{3}\right\}\}\\ \; & ≃\{\left\{V_{1}\right\},\left\{V_{2},V_{1}\right\},\left\{V_{2},V_{3}\right\}\} & (\mathrm{since}\;\left\{V_{1}\right\}\subseteq \left\{V_{1},V_{3}\right\})\\ \; & ≃\{\left\{V_{1}\right\},\left\{V_{2},V_{3}\right\}\}≃\alpha & (\mathrm{since}\;\left\{V_{1}\right\}\subseteq \left\{V_{2},V_{1}\right\})\\ \Xi (\Psi (\Psi (\alpha ))) & =\{\left\{V_{2},V_{3},V_{4}\right\},\left\{V_{2},V_{4}\right\},\left\{V_{3},V_{4}\right\},\left\{V_{1},V_{4}\right\}\}\\ \; & ≅\{\left\{V_{2},V_{3},V_{4}\right\},\left\{V_{3},V_{4}\right\},\left\{V_{1},V_{4}\right\}\} & (\mathrm{since}\;\left\{V_{2},V_{4}\right\}\subseteq \left\{V_{2},V_{3},V_{4}\right\})\\ \; & ≅\left\{\left\{V_{2},V_{3},V_{4}\right\},\left\{V_{1},V_{4}\right\}\right\}≅\Xi \left(\alpha \right) & (\mathrm{since}\;\left\{V_{3},V_{4}\right\}\subseteq \left\{V_{2},V_{3},V_{4}\right\}) \end{array}$$

**Lemma 2.**  *The function Ψ(·) is a bijection on the redundancy lattice without the bottom element (∅) that reverses its order. Let $\alpha ,\beta \in A\left(V\right)\setminus \left\{{⊥}_{\mathrm{RL}}\right\}$:*

1.
Ψ(Ψ(α))≃α

2.
α≼β⟺Ψ(β)≼Ψ(α)

**Lemma 3.**  *The function Ξ(·) is a bijection that maintains the ordering of atoms between the redundancy and synergy order. Let α,β∈A(V):*

1.
α=Ξ(Ξ(α))

2.
α≼β⟺Ξ(α)⪯Ξ(β)

The proofs of Lemmas 2 and 3 are given separately in Appendix D.

**Corollary 1.**  *Without bottom elements, the redundancy ($A\left(V\right)\setminus \left\{{⊥}_{\mathrm{RL}}\right\},≼$) and synergy lattice ($A\left(V\right)\setminus \left\{{⊥}_{\mathrm{SL}}\right\},⪯$) are related, as shown below with $\alpha ,\beta \in A\left(V\right)\setminus \left\{{⊥}_{\mathrm{RL}}\right\}$:*

(37a) $$\begin{array}{cc} \alpha ≼\beta & ⟺\Xi (\Psi (\beta ))⪯\Xi (\Psi (\alpha )) \end{array}$$

(37b) $$\begin{array}{cc} \Xi (\Psi (\alpha ⋏\beta )) & \;\;≅\;\;\Xi (\Psi (\alpha ))\vee \Xi (\Psi (\beta )) \end{array}$$

(37c) $$\begin{array}{cc} \Xi (\Psi (\alpha ⋎\beta )) & \;\;≅\;\;\Xi (\Psi (\alpha ))\wedge \Xi (\Psi (\beta )) \end{array}$$

(37d) $$\begin{array}{cc} \{\Xi \left(\Psi \left(\beta \right)\right)\;:\;\beta \in {↓}_{R}\alpha \}\;\; & {\;\left\{≅\right\}\;\;↑}_{S}\Xi \left(\Psi \left(\alpha \right)\right) \end{array}$$

(37e) $$\begin{array}{cc} \{\Xi \left(\Psi \left(\beta \right)\right)\;:\;\beta \in {↑}_{R}\alpha \}\;\; & {\;\left\{≅\right\}\;\;↓}_{S}\Xi \left(\Psi \left(\alpha \right)\right) \end{array}$$

**Proof.**  Follows directly from Lemma 2 and 3. □

**Figure 7:**
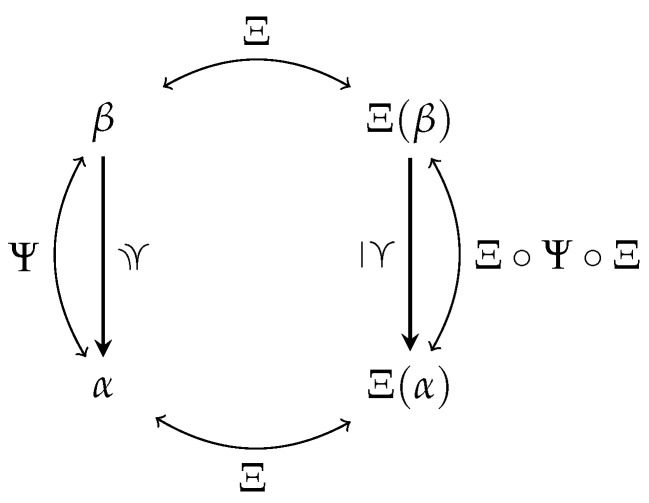
Visualization of the functions Ψ and Ξ: The application of function Ψ is equal to reversing the redundancy order, and the application of function Ξ is equal to swapping the ordering relation used between the redundancy and synergy lattice.

Figure 7 visualizes the relations from the introduced operators to provide an intuition. Applying the function Ψ to all atoms is equal to reversing the redundancy order, while applying the function Ξ to all atoms is equal to swapping the ordering relation used (synergy/redundancy order).

With these definitions in place, we can refine the definition of decomposition duality:

**Lemma 4**  (Decomposition duality). *A redundancy- and synergy-based information decomposition is pointwise dual if, for all $\alpha \in A\left(V\right)\setminus \left\{{⊥}_{\mathrm{RL}}\right\}$:*

(38) $$\begin{array}{cc} \Delta i_{\cap ,f}\left(\alpha ,T,t\right) & =\Delta i_{\cup ,f}\left(\Xi \left(\Psi \left(\alpha \right)\right),T,t\right)\\ \Delta i_{\cap ,f}\left({⊥}_{\mathrm{RL}},T,t\right) & =0=\Delta i_{\cup ,f}\left({⊥}_{\mathrm{SL}},T,t\right) \end{array}$$

*A redundancy- and synergy-based information decomposition is dual if, for all $\alpha \in A\left(V\right)\setminus \left\{{⊥}_{\mathrm{RL}}\right\}$:*

(39) $$\begin{array}{cc} \Delta I_{\cap ,f}\left(\alpha ;T\right) & =\Delta I_{\cup ,f}\left(\Xi \left(\Psi \left(\alpha \right)\right);T\right)\\ \Delta I_{\cap ,f}\left({⊥}_{\mathrm{RL}};T\right) & =0=\Delta I_{\cup ,f}\left({⊥}_{\mathrm{SL}};T\right) \end{array}$$

The proof of Lemma 4 is shown separately in Appendix D. To convert a decomposition from the synergy lattice into its dual-decomposition on to the redundancy lattice, the following relation is particularly useful. It states that, on the synergy lattice, all atoms are either in the up-set of Ξ(Ψ(α)) or in the down-set of an atom that corresponds to an individual source within α.

**Lemma 5.**  *For $\alpha \in A\left(V\right)\setminus \left\{{⊥}_{\mathrm{RL}}\right\}$:*

(40) $${A\left(V\right)\setminus ↑}_{S}\Xi \left(\Psi \left(\alpha \right)\right)=\underset{S_{a}\in \alpha }{⋃}{↓}_{S}\left\{S_{a}\right\}$$

**Proof.**  When expanding the definition of up- and down-sets, it can directly be seen from Lemma A9 that both sets provide an exclusive partitioning of all atoms.

(41) $$\begin{array}{ccc} {↑}_{S}\Xi \left(\Psi \left(\alpha \right)\right)= & \left\{\beta \in A\left(V\right)\;:\;\left(\Xi (\Psi (\alpha ))⪯\beta \right)\right\}\\ \underset{S_{a}\in \alpha }{⋃}{↓}_{S}\left\{S_{a}\right\}= & \left\{\beta \in A\left(V\right)\;:\;\left(\exists S_{a}\in \alpha .\;\beta ⪯\left\{S_{a}\right\}\right)\right\}\\ \left(\Xi (\Psi (\alpha ))⪯\beta \right) & ⟺\neg \left(\exists S_{a}\in \alpha .\;\beta ⪯\left\{S_{a}\right\}\right) & (\mathrm{by}\;\mathrm{Lemma}\;A9) \end{array}$$

□

**Figure 8:**
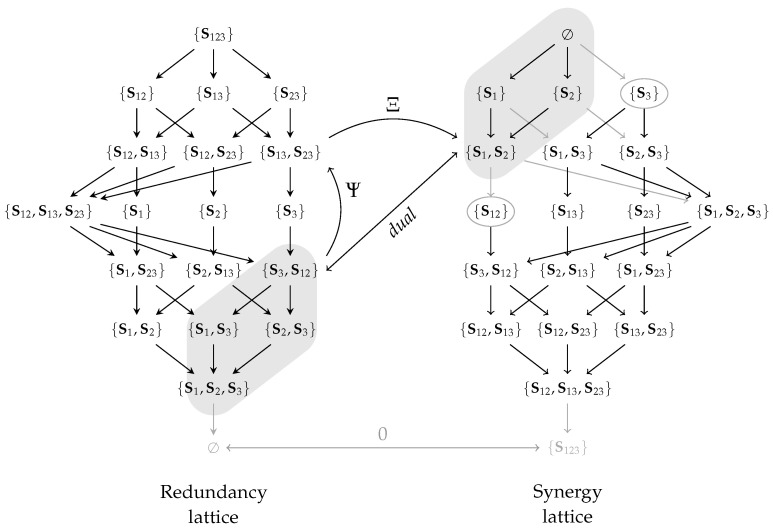
Visualization of lattice duality and Lemma 5. We abbreviate the notation of sources within this figure by listing the contained visible variables as source index ($S_{12}=\left\{V_{1},V_{2}\right\}$). (i) All bottom elements are mapped to each other and quantified to zero. (ii) To identify the dual for $\alpha =\{S_{3},S_{12}\}$ from the redundancy lattice, we first apply the transformation $\Psi \left(\alpha \right)≃\left\{S_{13},S_{23}\right\}$ and, then, $\Xi \left(\Psi \left(\alpha \right)\right)≅\left\{S_{1},S_{2}\right\}$. (iii) Ignoring the bottom elements, the down-set of α on the redundancy lattice corresponds to the up-set of Ξ(Ψ(α)) on the synergy lattice for duality (gray areas). (iv) Lemma 5 states that, on the synergy lattice, exactly those atoms that are not in the up-set of $\Xi (\Psi \left(\left\{S_{3},S_{12}\right\}\right))$ must be in the down-set of either $\left\{S_{3}\right\}$ or $\left\{S_{12}\right\}$.

Figure 8 summarizes and visualizes the required relations for the following transformation of the cumulative measure: (i) The bottom elements of all lattices are mapped to each other and quantified to zero. (ii) The function Ψ reverses the redundancy lattice (β≃Ψ(α) such that α≃Ψ(β)) to relate the down-set of α to the up-set of β while ignoring the bottom element. The function Ξ captures the relation between both orderings ($\alpha^{\prime }≅\Xi \left(\beta \right)$ such that $\beta ≃\Xi (\alpha^{\prime })$), to relate the up-set of β on the redundancy lattice to the up-set of $\alpha^{\prime }$ on the synergy lattice. This provides the desired mapping from the down-set of α on the redundancy lattice to the up-set of $\alpha^{\prime }$ on the synergy lattice for duality. Alternatively, we could first transform the down-set of α on the redundancy to the down-set of $\beta^{\prime }=\Xi \left(\alpha \right)$ on the synergy lattice, then reverse the synergy order and obtain the same result. (iii) Lemma 5 states that all atoms on the synergy lattice are either in the up-set of Ξ(Ψ(α)) or in the down-set of $\left\{S_{a}\right\}$ with $S_{a}\in \alpha$. The example $\alpha =\{S_{3},S_{12}\}$ is visualized in Figure 8, and we encourage the reader to view another example such as $\alpha =\left\{S_{13}\right\}\overset{\Psi }{\to }\left\{S_{1},S_{3}\right\}\overset{\Xi }{\to }\left\{S_{12},S_{23}\right\}$.

With these relations in place, we can construct dual-decompositions and prove their correctness.

**Lemma 6.**  *The pointwise dual-decomposition for the redundancy lattice of a loss measure on the synergy lattice is defined by:*

(42) $$i_{\cap ,f}\left(\alpha ,T,t\right):=\left\{\begin{array}{cc} 0 & \mathrm{if}\;\alpha =\emptyset \\ i_{\cup ,f}\left({⊤}_{\mathrm{SL}},T,t\right)-\sum_{\beta \in P_{1}\left(\alpha \right)}{\left(-1\right)}^{|\beta |-1}i_{\cup ,f}\left(\beta ,T,t\right) & \mathrm{otherwise} \end{array}\right.$$

The proof of Lemma 6 is shown separately in Appendix D. This section discussed the relation between four decomposition lattices, which are the redundancy and synergy lattice, as well as their reversed counterparts. Additionally, we demonstrated how this relation can be used to transform a cumulative decomposition measure between them. Decomposition duality enforces each lattice to be consistent with its set-theoretic interpretation. The function Ψ corresponds to taking the set-theoretic complement on the redundancy lattice and, thus, reflects on the cumulative measure by subtracting it from the top atom. The function Ξ corresponds to the relation between the union and intersection and, thus, introduces an inclusion–exclusion principle between their cumulative measures.

### 3.5. Decomposing f-Information on the Redundancy Lattice

Using the results from Section 3.4, we can now convert the decomposition of Section 3.2 to the redundancy lattice. The conversion can be applied to both the expected or pointwise measure. The partial contributions ($\Delta i_{\cap ,f}$ and $\Delta I_{\cap ,f}$) are obtained from the Möbius inverse.

**Lemma 7**  (Dual-decomposition on the redundancy lattice). *The definitions of Equation (43) correspond to the dual-decomposition of Definition 26.*

(43a) $$\begin{array}{cc} i_{\cap ,f}\left(\alpha ,T,t\right) & =\left\{\begin{array}{cc} 0 & \mathrm{if}\;\alpha =\emptyset \\ \sum_{\beta \in P_{1}\left(\alpha \right)}{\left(-1\right)}^{|\beta |-1}i_{f}\left(P_{T}\left(t\right),\kappa_{⊔}\left(\beta ,T,t\right)\right) & \mathrm{otherwise} \end{array}\right. \end{array}$$

(43b) $$\begin{array}{cc} I_{\cap ,f}\left(\alpha ;T\right) & =\sum_{t\in T}P_{T}\left(t\right)\cdot i_{\cap ,f}\left(\alpha ,T,t\right)=I_{f}\left(V;T\right)-\sum_{\beta \in P_{1}\left(\alpha \right)}{\left(-1\right)}^{|\beta |-1}I_{\cup ,f}\left(\beta ;T\right) \end{array}$$

**Proof.**  The duality of the pointwise measure is obtained from Lemma 6 and Definition 26. The duality of the pointwise measure implies the duality of the combined measure. □

The function $i_{f}\left(P_{T}\left(t\right),\kappa_{⊔}\left(\alpha ,T,t\right)\right)$ quantifies the convex hull/blackwell join of the Neyman–Pearson regions of its sources and represents a notion of pointwise union information about the target state t∈T. It is used in Equation (33a) to define a pointwise loss measure for the synergy lattice by subtracting it from the total information. As expected, we can see that the corresponding dual-decomposition on the redundancy lattice enforces an inclusion–exclusion relation between our notions of pointwise union information ($i_{f}\left(P_{T}\left(t\right),\kappa_{⊔}\left(\alpha ,T,t\right)\right)$) and pointwise intersection information ($i_{\cap ,f}\left(\alpha ,T,t\right)$).

**Theorem 4.**  *The dual-decomposition as defined by Equation (43) provides a non-negative PID, which satisfies an inclusion–exclusion relation and the axioms of Williams and Beer [1] on the redundancy lattice for any f-information.*

**Proof.** 

- **Axiom 1**: The measure $i_{\cap ,f}$ is invariant to permuting the order of sources in α, since the join operator of the zonogon order (${⨆}_{S\in \alpha }$) is. Therefore, also, $I_{\cap ,f}$ satisfies Axiom 1.
- **Non-negativity**: The non-negativity of $\Delta i_{\cap ,f}$ is obtained from Lemma 7 and Theorem 3 as shown in Equation (44). The non-negativity of the pointwise measure implies the non-negativity of the combined measure $\Delta I_{\cap ,f}$.
  (44) $$\forall \alpha \in A\left(V\right).\;\Delta i_{\cap ,f}\left(\alpha ,T,t\right)=\left\{\begin{array}{cc} 0 & \geq 0\;\mathrm{if}\;\alpha ={⊥}_{\mathrm{RL}}\\ \Delta i_{\cup ,f}\left(\Xi \left(\Phi \left(\alpha \right)\right),T,t\right)\; & \geq 0\;\mathrm{otherwise} \end{array}\right.$$
- **Axiom 2**: Since the cumulative measures $i_{\cap ,f}$ and $I_{\cap ,f}$ correspond to the sum of partial contributions in their down-set, the non-negativity of partial information implies the monotonicity of the cumulative measures.
- Axiom 3*: For a single source, $I_{\cap ,f}$ equals *f*-information by definition (see Equation (30)). Therefore, $I_{\cap ,f}$ satisfies Axiom 3*.

□

The operational interpretation of Section 3.3 is maintained since the partial contributions are identical between both lattices.

**Remark 8.**  *The definitions of Equations (34) and (43) satisfy the desired property of Bertschinger et al. [9], who argued that any sensible measure for unique and redundant information should only depend on the marginal distribution of sources.*

**Remark 9.**  *As discussed before [20], it is possible to further split redundancy into two components for extracting the pointwise meet under the Blackwell order (zonogon intersection, first component). The second component of redundancy as defined above contains decision regions that are part of the convex hull, but not the individual channel zonogons (discussed as shared information in [20]). By combining Equation (43) and Lemma A7, we obtain that both components of this split for redundancy are non-negative.*

### 3.6. Decomposing Rényi-Information

Since Rényi-information is an invertible transformation of Hellinger-information and α-information, we argue that their decompositions should be consistent. We propose to view the decomposition of Rényi-information as a transformation from an *f*-information and demonstrate the approach by transferring the Hellinger-information decomposition to a Rényi-information decomposition. Then, we demonstrate that the result is invariant to a linear scaling of the considered *f*-information, such that the transformation from α-information provides identical results. The obtained Rényi-information decomposition is non-negative and satisfies the three axioms proposed by Williams and Beer [1] (see below). However, its inclusion–exclusion relation is based on a transformed addition operator. For transforming the decomposition, we consider Rényi-information to be a re-graduation of Hellinger-information, as shown in Equation (45).

(45a) $$\begin{array}{cc} v_{a}\left(z\right): & =\frac{1}{a-1}\log\left(1+(a-1)z\right) \end{array}$$

(45b) $$\begin{array}{cc} I_{R_{a}}\left(S;T\right) & =v_{a}\left(I_{H_{a}}\left(S;T\right)\right) \end{array}$$

To maintain consistency when transforming the measure, we also have to transform its operators ([19], p. 6 ff.):

**Definition 29**  (Addition of Rényi-information). *We define the addition of Rényi-information ${⊕}_{a}$ with its corresponding inverse function ${⊖}_{a}$ by Equation (46).*

(46a) $$\begin{array}{cc} x{⊕}_{a}y & v_{a}\left(v_{a}^{-1}\left(x\right)+v_{a}^{-1}\left(y\right)\right)=\frac{\log\left(e^{(a-1)x}+e^{(a-1)y}-1\right)}{a-1} \end{array}$$

(46b) $$\begin{array}{cc} x{⊖}_{a}y & v_{a}\left(v_{a}^{-1}\left(x\right)-v_{a}^{-1}\left(y\right)\right)=\frac{\log\left(e^{(a-1)x}-e^{(a-1)y}+1\right)}{a-1} \end{array}$$

To transform a decomposition of the synergy lattice, we define the cumulative loss measures as shown in Equation (47) and use the transformed operators when computing the Möbius inverse (Equation (48a)) to maintain consistency in the results (Equation (48b)).

**Definition 30.**  *The cumulative and partial Rényi-information loss measures are defined as transformations of the cumulative and partial Hellinger-information loss measures, as shown in Equations (47) and (48).*

(47) $$I_{\cup ,R_{a}}(\alpha ;T):=v_{a}\left(I_{\cup ,H_{a}}\left(\alpha ;T\right)\right)$$

(48a) $$\begin{array}{cc} \Delta I_{\cup ,R_{a}}(\alpha ;T):= & I_{\cup ,R_{a}}\left(\alpha ;T\right)\;\;{⊖}_{a}\;\sum_{\beta \in {\dot{↓}}_{S}\alpha }\Delta I_{\cup ,R_{a}}\left(\beta ;T\right)\;\mathrm{where}:\;\;+:={⊕}_{a} \end{array}$$

(48b) $$\begin{array}{cc} \; & =v_{a}\left(\Delta I_{\cup ,H_{a}}\left(\alpha ;T\right)\right) \end{array}$$

**Remark 10.**  *We show in Lemma A11 of Appendix E that re-scaling the original f-information does not affect the resulting decomposition or transformed operators. Therefore, transforming a Hellinger-information decomposition or a α-information decomposition to a Rényi-information decomposition provides identical results.*

The operational interpretation presented in Section 3.2 is similarly applicable to partial Rényi-information ($\Delta I_{\cup ,R_{a}}$, Equation (48b)), since the function $v_{a}$ satisfies $v_{a}\left(0\right)=0$ and $x\leq 0⟹0\leq v_{a}\left(x\right)$.

**Theorem 5.**  The presented definitions for the cumulative loss measure $I_{\cup ,R_{a}}$ provide a non-negative PID on the synergy lattice with an inclusion–exclusion relation under the transformed addition (Definition 29) that satisfies Axioms 1, 2, and 3* for any Rényi-information measure.

**Proof.** 

- **Axiom 1**: $I_{\cup ,R_{a}}\left(\alpha ;T\right)$ is invariant to permuting the order of sources, since $I_{\cup ,H_{a}}\left(S;T\right)$ satisfies Axiom 1 (see Section 3.2).
- **Axiom 2**: $I_{\cup ,R_{a}}\left(\alpha ;T\right)$ satisfies monotonicity, since $I_{\cup ,H_{a}}\left(S;T\right)$ satisfies Axiom 2 (see Section 3.2) and the transformation function $v_{a}$ is monotonically increasing for a∈(0,1)∪(1,∞).
- Axiom 3*: Since $I_{\cup ,H_{a}}$ satisfies Axiom 3* (see Section 3.2, Equations (45) and (47)), $I_{\cup ,R_{a}}$ satisfies the self-redundancy axiom by definition, however, at a transformed operator: $I_{\cup ,R_{a}}\left(\left\{S_{i}\right\};T\right)=I_{R_{a}}\left(\left\{V\right\};T\right){⊖}_{a}I_{R_{a}}\left(\left\{S_{i}\right\};T\right)$.
- **Non-negativity**: The decomposition of $I_{\cup ,R_{a}}$ is non-negative, since $\Delta I_{\cup ,H_{a}}$ is non-negative (see Section 3.2), the Möbius inverse is computed with transformed operators (Equation (48b)) and the function $v_{a}$ satisfies $x\leq 0⟹0\leq v_{a}\left(x\right)$.

□

**Remark 11.**  *To obtain an equivalent decomposition of Rényi-information on the redundancy lattice, we can correspondingly transform the dual-decomposition from the redundancy lattice of Hellinger-information as shown in Equation (49). The resulting decomposition will satisfy the non-negativity, the axioms of Williams and Beer [1], and an inclusion–exclusion relation under the transformed operators (Definition 29) for the same reasons described above from Theorem 4.*

(49a) $$\begin{array}{cc} I_{\cap ,R_{a}}(\alpha ;T):= & v_{a}\left(I_{\cap ,H_{a}}\left(\alpha ;T\right)\right) \end{array}$$

(49b) $$\begin{array}{cc} \Delta I_{\cap ,R_{a}}(\alpha ;T):= & v_{a}\left(\Delta I_{\cap ,H_{a}}\left(\alpha ;T\right)\right) \end{array}$$

**Remark 12.**  *The relation between the redundancy and synergy lattice can be used for the definition of a bi-valuation [19] in calculations as discussed in [20]. This is also possible for Rényi-information at transformed operators.*

When taking the limit of Rényi-information for a→1, we obtain mutual information ($I_{\mathrm{KL}}$). Since mutual information is also an *f*-information, we expect its operators in the Möbius inverse to be addition. This is indeed the case (Equation (50)), and the measures will be consistent.

(50) $$\begin{array}{cc} \lim_{a\to 1}x{⊕}_{a}y & =x+y\\ \lim_{a\to 1}x{⊖}_{a}y & =x-y\; \end{array}$$

Finally, the decomposition of Bhattacharyya-information can be obtained by re-scaling the decomposition of Rényi-information at a=0.5, which causes another transform of the addition operator for the inclusion–exclusion relation.

## 4. Evaluation

A comparison of the proposed decomposition with other methods of the literature can be found in [20] for mutual information. Therefore, this section first compares different *f*-information measures for typical decomposition examples and discusses the special case of total variation (TV)-information to explain its distinct behavior. Since we can see larger differences between measures in more complex scenarios, we compare the measures by analyzing the information flows in a Markov chain. We provide the implementation used for both dual-decompositions of f-information and the examples used in this work in [30].

### 4.1. Partial Information Decomposition

#### 4.1.1. Comparison of Different f-Information Measures

**Figure 9:**
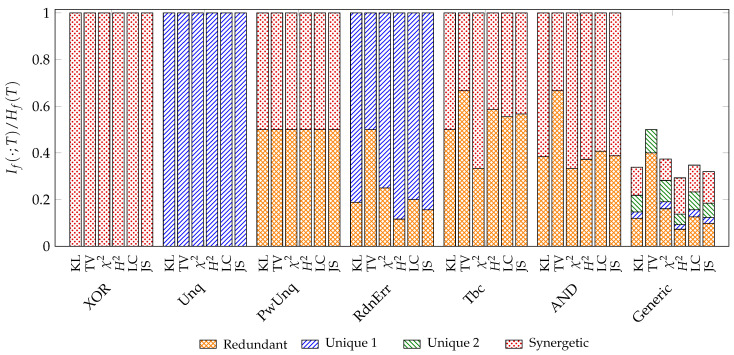
Comparison of different *f*-information measures normalized to the *f*-entropy of the target variable. All distributions are shown in Appendix F and correspond to the examples of [13,20]. The example name abbreviations are listed below in Table A1. The measures behave mostly similarly since the decompositions follow an identical structure. However, it can be seen that total variation attributes more information to being redundant than other measures and appears to behave differently in the generic example since it does not attribute any partial information to the first variable or their synergy.

We use the examples discussed by Finn and Lizier [13] to compare different *f*-information decompositions and add a generic example from [20]. All probability distributions used and their abbreviations can be found in Appendix F. We normalize the decomposition results to the *f*-entropy of the target variable for the visualization in Figure 9.

Since all results are based on the same framework, they behave similarly for examples that analyze a specific aspect of the decomposition function (XOR, Unq, PwUnq, RdnErr, Tbc, AND). However, it can be observed that the decomposition of total variation (TV) appears to differ from others: (1) In all examples, total variation attributes more information to being redundant than other measures. (2) In the generic example, total variation is the only measure that does not attribute any information to being unique to variable one or synergetic. We discuss the case of total variation in Section 4.1.2 to explain its distinct behavior.

**Figure A2:**
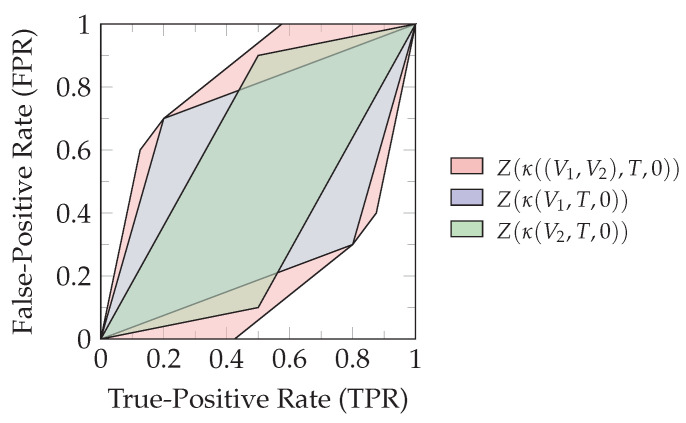
Visualization of the zonogons from the generic example of [20] in state t=0. The target variable *T* has two states. Therefore, the zonogons of its second state are symmetric (second column of Equation (6)) and have identical heights.

We visualize the zonogons for the generic example in Figure A2, which shall highlight that the implication of the operational interpretation does not hold in the other direction: the existence of partial information implies an advantage for the expected reward towards some state of the target variable, but an advantage for the expected reward towards some state of the target variable does not imply partial information in the example of total variation.

#### 4.1.2. The Special Case of Total Variation

**Figure 10:**
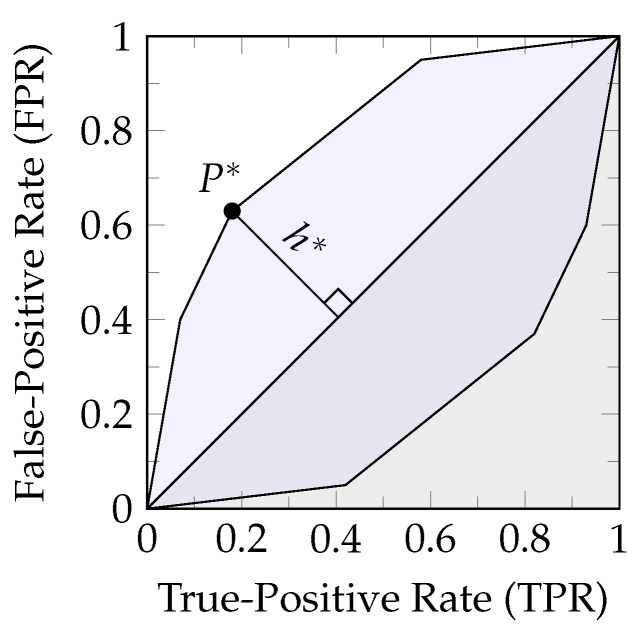
Visualization of the maximal (Euclidean) height $h^{*}$ at point $P^{*}$ that a zonogon (blue) reaches above the diagonal.

The behavior of total variation appears different compared to other *f*-information measures (Figure 9). This is due to total variation measuring the perimeter of a zonogon such that the result corresponds to a linear scaling of the maximal (Euclidean) height $h^{*}$ that the zonogon reaches above the diagonal, as visualized in Figure 10.

**Remark 13.**  From a cost perspective, the height $h^{*}$ can be interpreted as the performance evaluation of the optimal decision strategy (symmetric point to $P^{*}$ in the lower zonogon half) for a prediction $\hat{T}$ with minimal expected cost at the cost ratio $\frac{\mathrm{Cos}t\left(T=t,\hat{T}\neq t\right)-\mathrm{Cos}t\left(T=t,\hat{T}=t\right)}{\mathrm{Cos}t\left(T\neq t,\hat{T}=t\right)-\mathrm{Cos}t\left(T\neq t,\hat{T}\neq t\right)}=\frac{1-P_{T}\left(t\right)}{P_{T}\left(t\right)}$ (see Equation (8) of [31]) for each target state individually.

**Lemma 8.** 

(a) The pointwise total variation ($i_{\mathrm{TV}}$) is a linear scaling of the maximal (Euclidean) height $h^{*}$ that the corresponding zonogon reaches above the diagonal, as visualized in Figure 10 (Equation (51a)).
(b) *For a non-empty set of pointwise channels A, pointwise total variation $i_{\mathrm{TV}}$ quantifies the join element to the maximum of its individual channels (Equation (51b)).*
(c) *The loss measure $i_{\cup ,\mathrm{TV}}$ quantifies the meet for a set of sources on the synergy lattice to their minimum (Equation (51c)).*

(51a) $$\begin{array}{cc} i_{\mathrm{TV}}\left(p,\kappa \right) & =\frac{1-p}{2}\sum_{v\in \kappa }\left|v_{x}-v_{y}\right|=\left(1-p\right)\frac{h^{*}}{\sqrt{2}} \end{array}$$

(51b) $$\begin{array}{cc} i_{\mathrm{TV}}\left(p,\underset{\kappa \in A}{⨆}\kappa \right) & =\max_{\kappa \in A}\;i_{\mathrm{TV}}\left(p,\kappa \right) \end{array}$$

(51c) $$\begin{array}{cc} i_{\cup ,\mathrm{TV}}\left(\underset{\alpha \in A}{⋀}\alpha ,T,t\right) & =\min_{\alpha \in A}\;i_{\cup ,\mathrm{TV}}\left(\alpha ,T,t\right) \end{array}$$

**Proof.**  The proof of the first two statements (Equations (51a) and (51b)) is provided separately in Appendix G, which imply the third (Equation (51c)) by Definition 26. □

**Figure 11:**
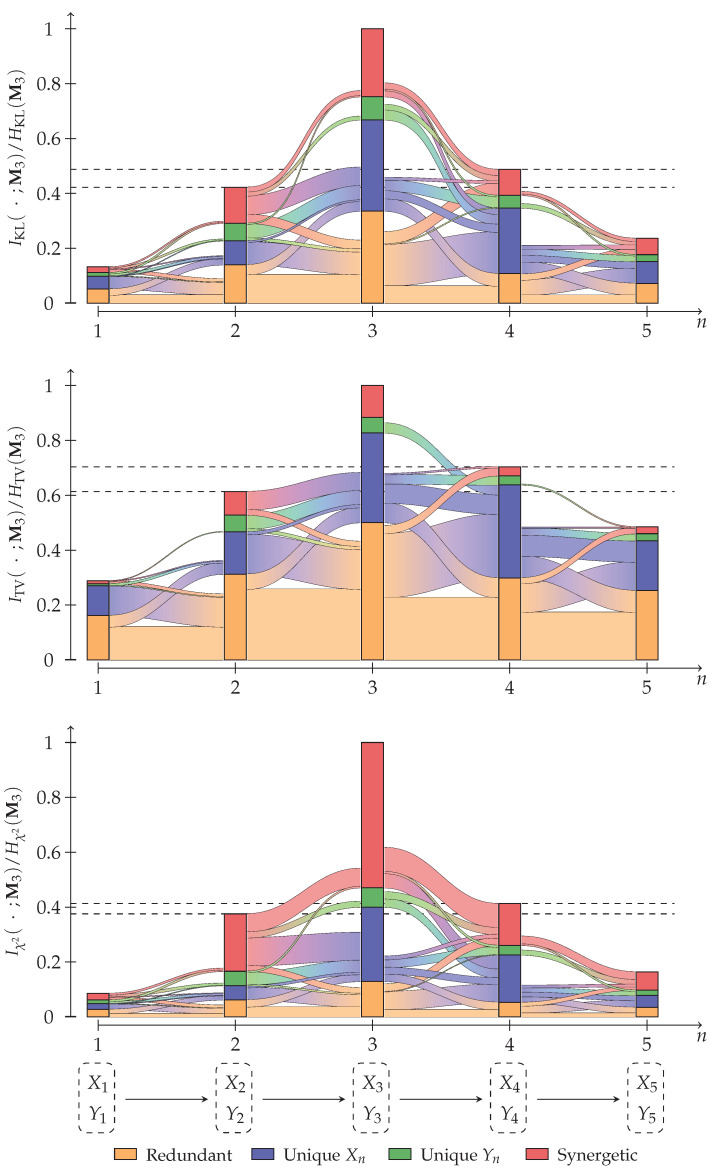
Analysis of the Markov chain information flow (Equation (Appendix H)). Visualized results for the information measures: KL, TV, and $\chi^{2}$. The remaining results ($H^{2}$-, LC-, and JS-information) can be found in Figure A3.

Quantifying the meet element on the synergy lattice to the minimum has the following consequences for total variation: (1) It attributes a minimum amount of synergy, and therefore more information to redundancy than other measures. (2) For each state of the target, at most one variable can provide unique information. In the case of |T|=2, the pointwise channels are symmetric (see Equation (6)), such that the same variable provides the maximal zonogon height both times. This is the case in the generic example of Figure 9, and the reason why at most one variable can provide unique information in this setting. However, beyond binary targets (|T|>2), both variables may provide unique information at the same time since different sources can provide the maximal zonogon height for different target states (see the later example in Figure 11).

**Remark 14.**  *Using the pointwise minimum on the synergy lattice results in a similar structure to the proposed measure of Williams and Beer [1]. However, TV-information is based on a different pointwise measure $i_{TV}$, which displays the same behavior (Equation (51b)), unlike pointwise KL-information.*

### 4.2. Information Flow Analysis

The differences between *f*-information measures in Section 4.1 appear more visible in complex scenarios. Therefore, this section compares different measures in the information flow analysis of a Markov chain.

Consider a Markov chain $M_{1}\to M_{2}\to \cdots \to M_{5}$, where $M_{i}=\left(X_{i},Y_{i}\right)$ is the joint distribution of two variables. Assume that we are interested in state three, and thus, define $T=M_{3}$ as the target variable. Using the approach described in Section 3, we can compute an information decomposition for each state $M_{i}$ of the Markov chain with respect to the target. Now, we are additionally interested in how the partial information decomposition from stage $M_{i}$ propagates into the next $M_{i+1}$, as visualized in Figure 11.

**Definition 31**  (Partial information flow). *The partial information flow of an atom $\alpha \in A(M_{i})$ into the atom $\beta \in A(M_{i+1})$ quantifies the redundancy between the partial contributions of their respective decomposition lattices.*

**Notation 8.**  *We use the notation $I_{\circ ,f}$ with ∘∈{∪,∩} to refer to either the loss measure $I_{\cup ,f}$ or redundancy measure $I_{\cap ,f}$. The same applies to the functions $J_{\circ \to \circ ,f}$ and $J_{\Delta \to \circ ,f}$ of Equation (52).*

Let $\alpha \in A(M_{i})$ and $\beta \in A(M_{i+1})$, then we compute information flows equivalently on the redundancy or synergy lattice as shown in Equation (52). When using a redundancy measure ∘=∩, then the strict down-set of ${\dot{↓}}_{\circ }\alpha$ refers to the strict down-set on its redundancy lattice $\left(A\left(M_{i}\right),≼\right)$, and when using a loss measure ∘=∪, then the strict down-set ${\dot{↓}}_{\circ }\alpha$ refers to the strict down-set on its synergy lattice $\left(A\left(M_{i}\right),⪯\right)$. We obtain the intersection of cumulative measures by quantifying their meet, which is on both lattice equivalent to their union of sources ($J_{\circ \to \circ ,f}$, Equation (52a)). To obtain how much of the partial contribution of α can be found in the cumulative measure of β ($J_{\Delta \to \circ ,f}$), we remove the contributions of its down-set (${\dot{↓}}_{\circ }\alpha$ on the lattice for $A(M_{i})$, see Equation (52b)). To finally obtain the flow from the partial contribution of α to the partial contribution of β ($J_{\Delta \to \Delta ,f}$), we similarly remove the contributions of the down-set of β (${\dot{↓}}_{\circ }\beta$ on the lattice for $A(M_{i+1})$, see Equation (52c)). The approach can be extended for tracing information flows over multiple steps; however, we will only trace one step in this example.

(52a) $$\begin{array}{cc} J_{\circ \to \circ ,f}(\alpha ,\beta ,T):= & I_{\circ ,f}\left(\alpha \cup \beta ;T\right) \end{array}$$

(52b) $$\begin{array}{cc} J_{\Delta \to \circ ,f}(\alpha ,\beta ,T):= & J_{\circ \to \circ ,f}(\alpha ,\beta ,T):=-\sum_{\gamma \in {\dot{↓}}_{\circ }\alpha }J_{\Delta \to \circ ,f}\left(\gamma ,\beta ,T\right) \end{array}$$

(52c) $$\begin{array}{cc} J_{\Delta \to \Delta ,f}\left(\alpha ,\beta ,T\right) & J_{\Delta \to \circ ,f}\left(\alpha ,\beta ,T\right)-\sum_{\gamma \in {\dot{↓}}_{\circ }\beta }J_{\Delta \to \Delta ,f}\left(\alpha ,\gamma ,T\right) \end{array}$$

**Remark 15.**  *The resulting partial information flows are equivalent (dual) between the redundancy and loss measure, except for the bottom element since their functionality differs: The flow from or to the bottom element on the redundancy lattice is always zero. In contrast, the flow from or to the bottom element on the synergy lattice quantifies the information gained or lost in the step.*

**Remark 16.**  *The information flow analysis of Rényi- and Bhattacharyya-information can be obtained as a transformation of the information flow from Hellinger-information. Alternatively, the information flow can be computed directly using Equation (52) under the corresponding definition of addition and subtraction for the information measure used.*

**Figure A3:**
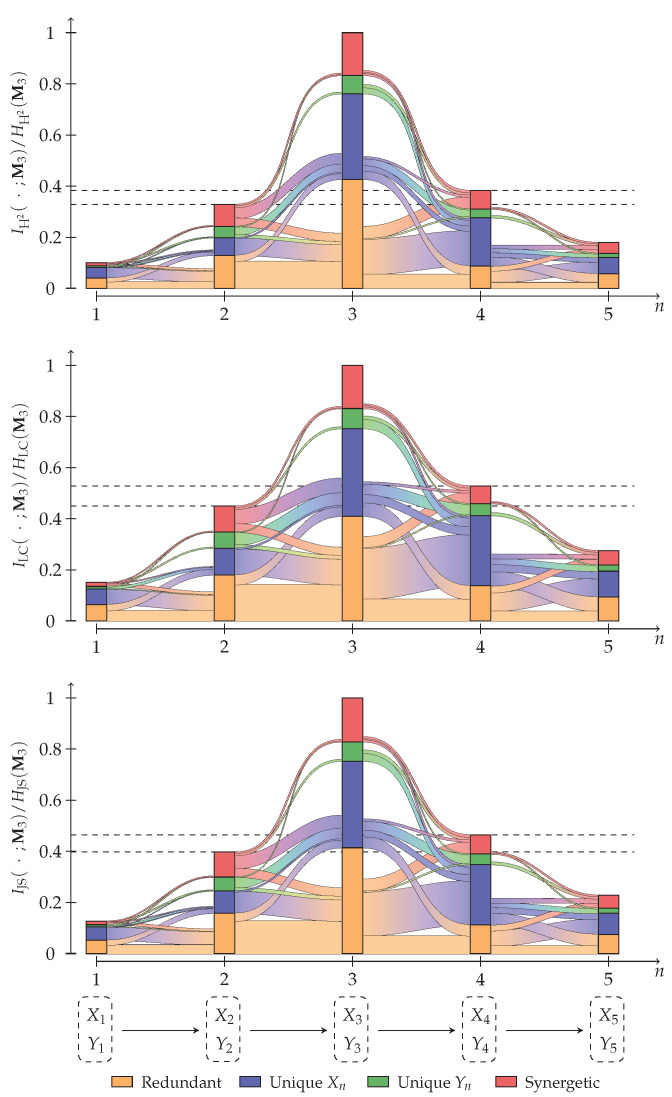
Analysis of the Markov chain information flow (Equation (A47)). Visualized results for the information measures: $H^{2}$, LC, and JS. The remaining results (KL, TV, and $\chi^{2}$) can be found in Figure 11.

We randomly generate an initial distribution and each row of a transition matrix under the constraint that at least one value shall be above 0.8 to avoid an information decay that is too rapid through the chain. The specific parameters of the example are shown in Appendix H. The event spaces used are X={0,1,2} and Y={0,1} such that $|M_{i}|=6$. We construct a Markov chain of five steps with the target $T=M_{3}$ and trace each partial information for one step using Equation (52). We visualized the results for KL-, TV-, and $\chi^{2}$-information in Figure 11, and the results for $H^{2}$-, LC-, and JS-information in Figure A3 of Appendix H.

All results display the expected behavior that the information that $M_{i}$ provides about $M_{3}$ increases for 1≤i≤3 and decreases for 3≤i≤5. The information flow results of KL-, $H^{2}$-, LC-, and JS-information are conceptually similar. Their main differences appear in the rate at which the information decays and, therefore, how much of the total information we can trace. In contrast, the results of TV- and $\chi^{2}$-information display different behavior, as shown in Figure 11: TV-information indicates significantly more redundancy, and $\chi^{2}$-information displays significantly more synergy than the other measures. Additionally, the decomposition of TV-information contains fever information flows. For example, it is the only analysis that does not show any information flow from $M_{2}$ into the unique contribution of $Y_{3}$ or from $M_{2}$ into the synergy of $\left(X_{3},Y_{3}\right)$. This demonstrates that the same decomposition method can obtain different behaviors from different *f*-divergences.

## 5. Discussion

Using the Blackwell order to construct pointwise lattices and to decompose pointwise information is motivated from the following three aspects:

- All information measures in Section 2.3 are the expected value of the pointwise information (quantification of the Neyman–Pearson region boundary) for an indicator variable of each target state. Therefore, we argue for acknowledging the “pointwise nature” [13] of these information measures and to decompose them accordingly. A similar argument was made previously by Finn and Lizier [13] for the case of mutual information and motivated their proposed pointwise partial information decomposition.
- The Blackwell order does not form a lattice beyond indicator variables since it does not provide a unique meet or join element for |T|>2 [17]. However, from a pointwise perspective, the informativity (Definition 2) provides a unique representation of union information. This enables separating the definition of redundant, unique, and synergetic information from a specific information measure, which then only serves for its quantification. We interpret these observations as an indication that the Blackwell order should be used to decompose pointwise information based on indicator variables rather than decomposing the expected information based on the full target distribution.
- We can consider where the alternative approach would lead, if we decomposed the expected information from the full target distribution using the Blackwell order: the decomposition would become identical to the method of Bertschinger et al. [9] and Griffith and Koch [10]. For bivariate examples (|V|=2), this decomposition [9,10] is non-negative and satisfies an additional property (*identity*, proposed by Harder et al. [5]). However, the identity property is inconsistent [32] with the axioms of Williams and Beer [1] and non-negativity for |V|>2. This causes negative partial information when extending the approach to |V|>2. The identity property also contradicts the conclusion of Finn and Lizier [13] from studying Kelly Gambling that, “information should be regarded as redundant information, regardless of the independence of the information sources” ([13], p. 26). It also contradicts our interpretation of distinct information through distinct decision regions when predicting an indicator variable for some target state. We do *not* argue that this interpretation should be applicable to the concept of information in general, but acknowledge that this behavior seems present in the information measures studied in this work and construct their decomposition accordingly.

Our critique for the decomposition measure of Williams and Beer [1] focuses on the implication that a less informative variable (Definition 2) about t∈T provides less pointwise information (I(S;T=t), Equation (15a)): $\kappa \left(S_{1},T,t\right)⊑\kappa \left(S_{2},T,t\right)⟹I\left(S_{1};T=t\right)\leq I\left(S_{2};T=t\right)$. This implication does not hold in the other direction. Therefore, equal pointwise information does not imply equal informativity and, thus, does not mean being redundant.

We chose to define a notion of pointwise union information based on the join of the Blackwell order since it leads to a meaningful operational interpretation: the convex hull of the pointwise Neyman–Pearson regions is always a subset of their joint distribution. Moreover, it is possible to construct joint distributions for which each individual decision region outside the convex hull becomes inaccessible, even if there may not exist one unique joint distribution at which all synergetic regions are lost simultaneously. This volatility due to the dependence between variables appears suitable for a notion of synergy. Similarly, the resulting unique information appears suitable since it ensures that a variable with unique information must provide access to some additional decision region. Finally, the obtained unique and redundant information is sensible [9] since it only depends on the marginal distributions with the target. The operational interpretation can be strengthened further such that the implication between accessible regions and partial information holds in both directions by revising Lemmas A1 and A2 with a strictly convex generator function.

We perform the decomposition on a pointwise lattice using the Blackwell join since it is possible to represent *f*-information as the expected value of quantifying the Neyman–Pearson region boundary (zonogon perimeter) for indicator variables (pointwise channels). Since the pointwise measures satisfy a triangle inequality, we mentioned the oversimplified intuition of pointwise *f*-information as the *length* of the zonogon perimeter. Correspondingly, if we identified an information measure that behaved more like the *area* of the zonogon (which could also maintain their ordering), then we would need to decompose it on a pointwise lattice using the Blackwell meet to achieve non-negativity. We assume that most information measures behave more similar to quantifying the boundary length rather than its area, since the boundary segments can directly be obtained from the conditional probability distribution and do not require an actual construction from the likelihood-ratio test.

In the literature, PIDs have been defined based on different ordering relations [16], the Blackwell order being only one of them. We think that this diversity is desirable since each approach provides a different operational interpretation of redundancy and synergy. For this reason, we wonder if obtaining a non-negative decomposition with the inclusion–exclusion relation for other ordering relations was possible when transferring them to a pointwise perspective or from mutual information to other information measures.

Studying the relations between different information measures for the same decomposition method may provide further insights into their properties, as demonstrated by the example of total variation in Section 4.2. The ability to decompose different information measures is also a necessity to apply the method in a variety of areas, since each information measure can then provide the operational meaning within its respective domains. To ensure consistency between related information measures, we allowed the re-definition of information addition, as demonstrated in the example of Rényi-information in Section 3.6, which also opens new possibilities for satisfying the inclusion–exclusion relation.

There is currently no universally accepted definition of conditional Rényi information. Assuming that $I_{R_{a}}\left(T;S_{i}|S_{j}\right)$ should capture the information that $S_{i}$ provides about *T* when already knowing the information from $S_{j}$, then one could propose that this quantity should correspond to the according partial information contributions (unique/synergetic) and, thus, the definition of Equation (53).

With this in mind, it is also possible to define, model, decompose, and trace *Transfer Entropy* [33], used in the analysis of complex systems, for each presented information measure with the methodology of Section 4.2.

(53) $$I_{R_{a}}\left(T;S_{i}|S_{j}\right)I_{R_{a}}\left(T;S_{i},S_{j}\right)⊖I_{R_{a}}\left(T;S_{j}\right)$$

Finally, studying the corresponding definitions for continuous random variables and identifying suitable information measures for specific applications would be interesting directions for future work.

## 6. Conclusions

In this work, we demonstrated a non-negative PID in the framework of Williams and Beer [1] for any *f*-information with practical operational interpretation and the conversion of measures between decomposition lattices. We demonstrated that the decomposition of *f*-information can be used to obtain a non-negative decomposition of Rényi-information, for which we re-defined the addition to demonstrate that its results satisfy an inclusion–exclusion relation. Finally, we demonstrated how the proposed decomposition method can be used for tracing the flow of information through Markov chains and how the decomposition obtains different properties depending on the chosen information measure.

## Figures and Tables

**Table 1:** Summary of the notation used for the redundancy and synergy lattice.

	Redundancy Order	Synergy Order
Ordering/equivalence	≼/≃	⪯/≅
Join/meet	⋎/⋏	∨/∧
Up-set/strict up-set	${↑}_{R}$/${\dot{↑}}_{R}$	${↑}_{S}$/${\dot{↑}}_{S}$
Down-set/strict down-set	${↓}_{R}$/${\dot{↓}}_{R}$	${↓}_{S}$/${\dot{↓}}_{S}$
Cover-set	$\alpha^{{-}_{R}}$	$\alpha^{{-}_{S}}$
Top/bottom	${⊤}_{\mathrm{RL}}=\left\{V\right\}$/${⊥}_{\mathrm{RL}}=\emptyset$	${⊤}_{\mathrm{SL}}=\emptyset$/${⊥}_{\mathrm{SL}}=\left\{V\right\}$

**Table 2:** Commonly used functions for *f*-divergences.

Notation	Name	Generator Function
$D_{\mathrm{KL}}$	Kullback-Leiber (KL)-divergence	f(z)=zlogz
$D_{\mathrm{TV}}$	Total Variation (TV)	$f\left(z\right)=\frac{1}{2}\left|z-1\right|$
$D_{\chi^{2}}$	$\chi^{2}$-divergence	$f\left(z\right)={\left(z-1\right)}^{2}$
$D_{H^{2}}$	Squared Hellinger distance	$f\left(z\right)={\left(1-\sqrt{z}\right)}^{2}$
$D_{\mathrm{LC}}$	Le Cam distance	$f\left(z\right)=\frac{1-z}{2z+2}$
$D_{\mathrm{JS}}$	Jensen–Shannon divergence	$f\left(z\right)=z\log\frac{2z}{z+1}+\log\frac{2}{z+1}$
$D_{H_{a}}$	Hellinger-divergence with a∈(0,1)∪(1,∞)	$f\left(z\right)=\frac{z^{a}-1}{a-1}$
$D_{\alpha =a}$	α-divergence with a∈(0,1)∪(1,∞)	$f\left(z\right)=\frac{z^{a}-1-a\left(z-1\right)}{a(a-1)}$

**Figure A1:**
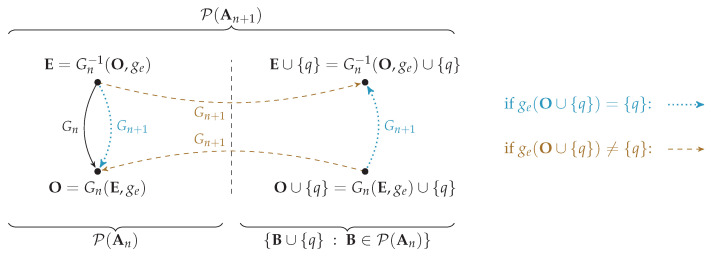
Intuition for the definition of Equation (A8). We can divide the set $P(A_{n+1})$ into $P(A_{n})$ and $\left\{B\cup \left\{q\right\}\;:\;B\in P\left(A_{n}\right)\right\}$. The definition of function $G_{n+1}$ mirrors $G_{n}$ if $g_{e}\left(O\cup \left\{q\right\}\right)=\left\{q\right\}$ (blue) and otherwise breaks its mapping (orange).

## Appendix A. Quantifying Zonogon Perimeters

**Lemma A1.** 
*If the function f is convex, then the function $r_{f}\left(p,\vec{v}\right)$ as defined in Equation (28a) is convex in its second argument $\vec{v}\in {\left[0,1\right]}^{2}$ for a constant p∈[0,1].*

**Proof.** We use the following definitions for abbreviating the notation. Let 0≤t≤1 and ${\vec{v}}_{i}=\left[\begin{array}{c} x_{i}\\ y_{i} \end{array}\right]$:

$$\begin{array}{cc} a_{1}:= & x_{1}p+y_{1}\left(1-p\right)\\ a_{2}:= & x_{2}p+y_{2}\left(1-p\right)\\ b_{1}:= & \frac{ta_{1}}{ta_{1}+\left(1-t\right)a_{2}}\\ b_{2}:= & \frac{\left(1-t\right)a_{2}}{ta_{1}+\left(1-t\right)a_{2}} \end{array}$$

The case of $a_{i}=0$ is handled by the convention that $0\cdot f\left(\frac{0}{0}\right)=0$. Therefore, we can assume that $a_{i}\neq 0$ and use $0\leq b_{1}\leq 1$ with $b_{2}=1-b_{1}$ to apply the definition of convexity on the function *f*:

$$\begin{array}{ccc} r_{f}\left(p,\left[\begin{array}{c} tx_{1}+\left(1-t\right)x_{2}\\ ty_{1}+\left(1-t\right)y_{2} \end{array}\right]\right) & =\left(ta_{1}+\left(1-t\right)a_{2}\right)\cdot f\left(\frac{tx_{1}+\left(1-t\right)x_{2}}{ta_{1}+\left(1-t\right)a_{2}}\right)\\ \; & =\left(ta_{1}+\left(1-t\right)a_{2}\right)\cdot f\left(b_{1}\frac{x_{1}}{a_{1}}+b_{2}\frac{x_{2}}{a_{2}}\right)\\ \; & \leq \left(ta_{1}+\left(1-t\right)a_{2}\right)\cdot \left(b_{1}f\left(\frac{x_{1}}{a_{1}}\right)+b_{2}f\left(\frac{x_{2}}{a_{2}}\right)\right)\; & (\mathrm{by}\;\mathrm{convexity}\;\mathrm{of}\;f)\\ \; & =ta_{1}\cdot f\left(\frac{x_{1}}{a_{1}}\right)+\left(1-t\right)a_{2}\cdot f\left(\frac{x_{2}}{a_{2}}\right)\\ \; & =t\cdot r_{f}\left(p,\left[\begin{array}{c} x_{1}\\ y_{1} \end{array}\right]\right)+\left(1-t\right)\cdot r_{f}\left(p,\left[\begin{array}{c} x_{2}\\ y_{2} \end{array}\right]\right) \end{array}$$

□

**Corollary A1.** 
*For ${\vec{v}}_{1},{\vec{v}}_{2},\left({\vec{v}}_{1}+{\vec{v}}_{2}\right)\in {\left[0,1\right]}^{2}$ and a constant p∈[0,1], the function $r_{f}\left(p,\vec{v}\right)$ as defined in Equation (28a) satisfies a triangle inequality on its second argument: $r_{f}\left(p,{\vec{v}}_{1}+{\vec{v}}_{2}\right)\leq r_{f}\left(p,{\vec{v}}_{1}\right)+r_{f}\left(p,{\vec{v}}_{2}\right)$.*

**Proof.** 

$$\begin{array}{ccc} r_{f}\left(p,\ell {\vec{v}}_{1}+\left(1-\ell \right){\vec{v}}_{2}\right) & \leq \ell r_{f}\left(p,{\vec{v}}_{1}\right)+\left(1-\ell \right)r_{f}\left(p,{\vec{v}}_{2}\right) & (\mathrm{be}\;\mathrm{Lemma}\;A1)\\ r_{f}\left(p,0.5({\vec{v}}_{1}+{\vec{v}}_{2})\right) & \leq 0.5\left(r_{f}\left(p,{\vec{v}}_{1}\right)+r_{f}\left(p,{\vec{v}}_{2}\right)\right) & (\mathrm{let}\;\ell =0.5)\\ r_{f}\left(p,{\vec{v}}_{1}+{\vec{v}}_{2}\right) & \leq r_{f}\left(p,{\vec{v}}_{1}\right)+r_{f}\left(p,{\vec{v}}_{2}\right) & (\mathrm{by}\;r_{f}\left(p,\ell \vec{v}\right)=\ell r_{f}\left(p,\vec{v}\right)) \end{array}$$

□

**Lemma A2.** 
*For a constant p∈[0,1], the function $i_{f}$ maintains the ordering relation from the Blackwell order on binary input channels: $\kappa_{1}⊑\kappa_{2}⟹i_{f}\left(p,\kappa_{1}\right)\leq i_{f}\left(p,\kappa_{2}\right)$.*

**Proof.** Let $\kappa_{1}$ be represented by a 2×n matrix and $\kappa_{2}$ by a 2×m matrix. By the definition of the Blackwell order ($\kappa_{1}⊑\kappa_{2}$, Equation (2)), there exists a stochastic matrix λ such that $\kappa_{1}=\kappa_{2}\cdot \lambda$. We use the notation $\kappa_{2}\left[:,i\right]$ to refer to the $i^{\mathrm{th}}$ column of matrix $\kappa_{2}$ and indicate the element at row i∈{1..m} and column j∈{1..n} of λ by λ[i,j]. Since λ is a valid (row) stochastic matrix of dimension m×n, its rows sum to one $\forall i\in \left\{1..m\right\}.\;\sum_{j=1}^{n}\lambda \left[i,j\right]=1$.

$$\begin{array}{ccc} i_{f}\left(p,\kappa_{1}\right) & =\sum_{j=1}^{n}r_{f}\left(p,\kappa_{1}\left[:,j\right]\right) & (\mathrm{by}\;\mathrm{Equation}\;(28b))\\ \; & =\sum_{j=1}^{n}r_{f}\left(p,\sum_{i=1}^{m}\kappa_{2}\left[:,i\right]\lambda \left[i,j\right]\right) & (\mathrm{by}\;\mathrm{Equation}\;(2))\\ \; & \leq \sum_{j=1}^{n}\sum_{i=1}^{m}r_{f}\left(p,\kappa_{2}\left[:,i\right]\lambda \left[i,j\right]\right) & (\mathrm{by}\;\mathrm{Corollary}\;A1)\\ \; & =\sum_{j=1}^{n}\sum_{i=1}^{m}\lambda \left[i,j\right]r_{f}\left(p,\kappa_{2}\left[:,i\right]\right)\; & (\mathrm{by}\;r_{f}\left(p,\ell \vec{v}\right)=\ell r_{f}\left(p,\vec{v}\right))\\ \; & =\sum_{i=1}^{m}r_{f}\left(p,\kappa_{2}\left[:,i\right]\right)\; & (\mathrm{by}\;\sum_{j=1}^{n}\lambda \left[i,j\right]=1)\\ \; & =i_{f}\left(p,\kappa_{2}\right) & (\mathrm{by}\;\mathrm{Equation}\;(28b)) \end{array}$$

□

**Lemma A3.** 
*Consider two non-empty sets of binary input channels with equal cardinality (|A|=|B|) and a constant p∈[0,1]. If the Minkowski sum for the zonogons of channels in A is a subset of the Minkowski sum for the zonogons of channels in B, then the sum of pointwise information for the channels in A is less than the sum of pointwise information for the channels in B as shown in Equation (A1).*

(A1)
$$\sum_{\kappa \in A}Z\left(\kappa \right)\subseteq \sum_{\kappa \in B}Z\left(\kappa \right)⟹\sum_{\kappa \in A}i_{f}\left(p,\kappa \right)\leq \sum_{\kappa \in B}i_{f}\left(p,\kappa \right)$$

**Proof.** Let n=|A|=|B|. We use the notation A[i] with 1≤i≤n to indicate the channel $\kappa_{i}$ within the set A.

$$\begin{array}{ccc} \sum_{i=1}^{n}Z\left(A\left[i\right]\right) & \subseteq \sum_{i=1}^{n}Z\left(B\left[i\right]\right)\\ Z\left(\left[\begin{array}{ccc} A[1] & \cdots & A[n] \end{array}\right]\right) & \subseteq Z\left(\left[\begin{array}{ccc} B[1] & \cdots & B[n] \end{array}\right]\right) & (\mathrm{by}\;\mathrm{Equation}\;(4))\\ Z\left(\frac{1}{n}\cdot \left[\begin{array}{ccc} A[1] & \cdots & A[n] \end{array}\right]\right) & \subseteq Z\left(\frac{1}{n}\cdot \left[\begin{array}{ccc} B[1] & \cdots & B[n] \end{array}\right]\right) & (\mathrm{scale}\;\mathrm{to}\;\mathrm{sum}\;\left(1,1\right))\\ i_{f}\left(p,\frac{1}{n}\cdot \left[\begin{array}{ccc} A[1] & \cdots & A[n] \end{array}\right]\right) & \leq i_{f}\left(p,\frac{1}{n}\cdot \left[\begin{array}{ccc} B[1] & \cdots & B[n] \end{array}\right]\right) & (\mathrm{by}\;\mathrm{Equation}\;(5),\;\mathrm{Lemma}\;A2)\\ \sum_{i=1}^{n}i_{f}\left(p,\frac{1}{n}A\left[i\right]\right) & \leq \sum_{i=1}^{n}i_{f}\left(p,\frac{1}{n}B\left[i\right]\right) & (\mathrm{by}\;\mathrm{Equation}\;(28b))\\ \frac{1}{n}\sum_{i=1}^{n}i_{f}\left(p,A[i]\right) & \leq \frac{1}{n}\sum_{i=1}^{n}i_{f}\left(p,B[i]\right) & (\mathrm{by}\;r_{f}\left(p,\ell \vec{v}\right)=\ell r_{f}\left(p,\vec{v}\right))\\ \sum_{\kappa \in A}i_{f}\left(p,\kappa \right) & \leq \sum_{\kappa \in B}i_{f}\left(p,\kappa \right) \end{array}$$

□

## Appendix B. Inclusion-Exclusion Inequality of Zonogons

Let P(A) represent the power set of a non-empty set A≠∅ and separate the subsets of even ($L_{e}$) and odd ($L_{o}$) cardinality as shown below. Additionally, let $L_{\leq 1}$ represent all subsets with cardinality less than or equal to one and $L_{1}$ all subsets of cardinality equal to one:

(A2) $$\begin{array}{cc} L_{\leq 1}:= & \left\{B\in P(A)\;:\;|B|\leq 1\right\}\\ L_{1}:= & \left\{B\in P(A)\;:\;|B|=1\right\}\\ L_{e}:= & \left\{B\in P(A)\;:\;|B|even\right\}\\ L_{o}:= & \left\{B\in P(A)\;:\;|B|odd\right\}\\ P(A):= & =L_{e}\cup L_{o}\;\mathrm{and}\;\emptyset =L_{e}\cap L_{o} \end{array}$$

The number of subsets with even cardinality is equal to the number of subsets with odd cardinality as shown in Equation (A3).

(A3) |Le|=∑i=0|A|2|A|2i=2|A|−1=∑i=0|A|2|A|2i+1=|Lo|

Consider a function $g_{e}:L_{e}\to L_{\leq 1}$, which takes an even subset $E\in L_{e}$ and returns a subset of cardinality $|g_{e}\left(E\right)|=\min(|E|,1)$ according to Equation (A4).

(A4) $$\forall E\in S_{e}\;:\;\left\{\begin{array}{cc} g_{e}\left(E\right)=\emptyset & \mathrm{if}E=\emptyset \\ g_{e}\left(E\right)=\left\{e\right\}\;\;s.t.e\in E & \mathrm{otherwise} \end{array}\right.$$

**Lemma A4.** 
*For any function $g_{e}\in G_{e}$, there exists a function $G:\left(L_{e},G_{e}\right)\to L_{o}$ that satisfies the following two properties:*

(a) *For any subset with even cardinality, the function $g_{e}\left(\cdot \right)$ returns a subset of function G(·):*
  (A5)
  $$\forall g_{e}\in G_{e},E\in L_{e}\;:\;g_{e}\left(E\right)\subseteq G\left(E,g_{e}\right).$$
(b) *The function G(·) that satisfies Equation (A5) has an inverse on its first argument $G^{-1}:\left(L_{o},G_{e}\right)\to L_{e}$.*
  (A6)
  $$\forall g_{e}\in G_{e},E\in L_{e},\exists G^{-1}:\;G^{-1}\left(G\left(E,g_{e}\right),g_{e}\right)=E.$$

**Proof.** We construct a function *G* for an arbitrary $g_{e}$ and demonstrate that it satisfies both properties (Equations (A5) and A6) by induction on the cardinality of A. We indicate the cardinality of A with n=|A| as subscripts $A_{n}$, $L_{e,n}$, $L_{o,n}$, and $G_{n}$:

1. In the base case $A_{1}=\left\{a\right\}$, the sets of subsets are $L_{e,1}=\left\{\emptyset \right\}$ and $L_{o,1}=\left\{\left\{a\right\}\right\}$. We define the function $G_{1}\left(\emptyset ,g_{e}\right)\left\{a\right\}$ for any $g_{e}$ to satisfy both required properties:
  (a) The constraints of Equation (A4) ensure that $g_{e}\left(\emptyset \right)=\emptyset$. Since the empty set is the only element in $S_{e,1}$, the subset relation (requirement of Equation (A5)) is satisfied $g_{e}\left(\emptyset \right)=\emptyset \subseteq \left\{a\right\}=G_{1}\left(\emptyset ,g_{e}\right)$.
  (b) The function $G_{1}:\left(L_{e,1},G_{e}\right)\to L_{o,1}$ is a bijection from $L_{e,1}$ to $L_{o,1}$ and, therefore, has an inverse on its first argument $G_{1}^{-1}:\left(L_{o,1},G_{e}\right)\to L_{e,1}$ (requirement of Equation (A6)).
2. Assume there exists a function $G_{n}$ that satisfies both required properties (Equations (A5) and (A6)) of sets of cardinality $1\leq n=|A_{n}|$.
3. For the induction step, we show the definition of a function $G_{n+1}$ that satisfies both required properties. For sets $A_{n+1}=A_{n}\cup \left\{q\right\}$, the subsets of even and odd cardinality can be expanded as shown in Equation (A7).
  (A7) $$\begin{array}{cc} L_{e,n+1} & =L_{e,n}\;\cup \;\left\{O\cup \left\{q\right\}\;:\;O\in L_{o,n}\right\},\\ L_{o,n+1} & =L_{o,n}\;\cup \;\left\{E\cup \left\{q\right\}\;:\;E\in L_{e,n}\right\}. \end{array}$$
  We define $G_{n+1}$ for $E\in L_{e,n}$ and $O\in L_{o,n}$ at any $g_{e}$ as shown in Equation (A8) using the function $G_{n}$ and its inverse $G_{n}^{-1}$ from the induction hypothesis. The function $G_{n+1}$ is defined for any subset in $L_{e,n+1}$ as can be seen from Equation (A7).
  (A8) $$\begin{array}{cc} G_{n+1}(E,g_{e}):= & \left\{\begin{array}{cc} E\cup \{q\}\;\; & \mathrm{if}g_{e}\left(G_{n}\left(E,g_{e}\right)\cup \left\{q\right\}\right)\neq \left\{q\right\}\\ G_{n}\left(E,g_{e}\right) & \mathrm{if}g_{e}\left(G_{n}\left(E,g_{e}\right)\cup \left\{q\right\}\right)=\left\{q\right\} \end{array}\right.\\ G_{n+1}(O\cup \left\{q\right\},g_{e}):= & \left\{\begin{array}{cc} O & \mathrm{if}g_{e}\left(O\cup \left\{q\right\}\right)\neq \left\{q\right\}\\ G_{n}^{-1}\left(O,g_{e}\right)\cup \left\{q\right\} & \mathrm{if}g_{e}\left(O\cup \left\{q\right\}\right)=\left\{q\right\} \end{array}\right. \end{array}$$
  Figure A1 provides an intuition for the definition of $G_{n+1}$: the outcome of $g_{e}\left(O\cup \left\{q\right\}\right)$ determines if the function $G_{n+1}$ maintains or breaks the mapping of $G_{n}$.
  The function *F* as defined in Equation (A8) satisfies both requirements (Equations (A5) and (A6)) for any $g_{e}$:
  (a) To demonstrate that the function satisfies the subset relation of Equation (A5), we analyze the four cases for the return value of $G_{n+1}$ as defined in Equation (A8) individually:
    - $g_{e}\left(E\right)\subseteq E\cup \left\{q\right\}$ holds, since the function $g_{e}$ always returns a subset of its input (Equation (A4)).
    - $g_{e}\left(E\right)\subseteq G_{n}\left(E,g_{e}\right)$ holds by the induction hypothesis.
    - If $g_{e}\left(O\cup \left\{q\right\}\right)\neq \left\{q\right\}$, then $g_{e}\left(O\cup \left\{q\right\}\right)\subseteq O$: Since the input to function $g_{e}$ is not the empty set, the function $g_{e}\left(O\cup \left\{q\right\}\right)$ returns a singleton subset of its input (Equation (A4)). If the element in the singleton subset is unequal to *q*, then it is a subset of O.
    - If $g_{e}\left(O\cup \left\{q\right\}\right)=\left\{q\right\}$, then $g_{e}\left(O\cup \left\{q\right\}\right)\subseteq \left\{q\right\}\cup G_{n}^{-1}\left(O,g_{e}\right)$ holds trivially.
  (b) To demonstrate that the function $G_{n+1}$ has an inverse (Equation (A6)), we show that the function $G_{n+1}$ is a bijection from $L_{e,n+1}$ to $L_{o,n+1}$. Since the function $G_{n+1}$ is defined for all elements in $L_{e,n+1}$ and both sets have the same cardinality ($|L_{e,n+1}\left|=\right|L_{o,n+1}|$, Equation (A3)), it is sufficient to show that the function $G_{n+1}$ is distinct for all inputs.
    The return value of $G_{n+1}$ has four cases, two of which return a set containing *q* (cases 1 and 4 in Equation (A8)), while the two others do not (cases 2 and 3 in Equation (A8)). Therefore, we have to show that both of these cases cannot coincide for any input:
    - Cases 2 and 3 in Equation (A8): If the return value of both cases was equal, then $O=G_{n}\left(E,g_{e}\right)$, and therefore, $g_{e}\left(O\cup \left\{q\right\}\right)=g_{e}\left(G_{n}\left(E,g_{e}\right)\cup \left\{q\right\}\right)$. This leads to a contradiction, since the condition of case 3 ensures $g_{e}\left(O\cup \left\{q\right\}\right)\neq \left\{q\right\}$, while the condition of case 2 ensures $g_{e}\left(G_{n}\left(E,g_{e}\right)\cup \left\{q\right\}\right)=\left\{q\right\}$. Hence, the return values of cases 2 and 3 are distinct.
    - Cases 1 and 4 in Equation (A8): If the return value of both cases was equal, then $E=G_{n}^{-1}\left(O,g_{e}\right)$, and therefore, $g_{e}\left(O\cup \left\{q\right\}\right)=g_{e}\left(G_{n}\left(E,g_{e}\right)\cup \left\{q\right\}\right)$. This leads to a contradiction, since the condition of case 4 ensures $g_{e}\left(O\cup \left\{q\right\}\right)=\left\{q\right\}$, while the condition of case 1 ensures $g_{e}\left(G_{n}\left(E,g_{e}\right)\cup \left\{q\right\}\right)\neq \left\{q\right\}$. Hence, the return values of cases 1 and 4 are distinct.
    Since the function $G_{n+1}$ is a bijection, there exists an inverse $G_{n+1}^{-1}$.

□

**Lemma A5.** 
*For a non-empty set of 2×x row stochastic matrices A≠∅:*

(A9)
$$Z\left({⊓}_{\kappa \in A}\kappa \right)+\sum_{\overset{\emptyset \neq B\subseteq A}{|B|\;\mathrm{even}}}\;Z\left(\underset{\lambda \in B}{⨆}\lambda \right)\subseteq \sum_{\overset{B\subseteq A}{|B|\;\mathrm{odd}}}\;Z\left(\underset{\nu \in B}{⨆}\nu \right)$$

**Proof.** Consider a function $g_{o}:L_{o}\to L_{1}$, where $g_{o}\left(O\right)\subseteq O$ such that the function returns a singleton subset for a set of odd cardinality. Equation (A10) can be obtained from the constraints on $g_{e}$ (Equation (A4)) and Lemma A4.

(A10) $$\forall g_{e}\in G_{e},E\in L_{e},\exists g_{o}\in G_{o},G\;:\;\left\{\begin{array}{cc} g_{e}\left(\emptyset \right)\subseteq g_{o}\left(G\left(\emptyset \right)\right) & \mathrm{if}E=\emptyset \\ g_{e}\left(E\right)=g_{o}\left(G\left(E\right)\right) & \mathrm{otherwise} \end{array}\right.$$

Equation (A11a) holds since we can replace $g_{e}\left(\emptyset \right)$ with $g_{o}\left(G\left(\emptyset \right)\right)$, meaning there exists a κ∈A for creating a (Minkowski) sum over the same set of channel zonogons on both sides of the quality. Equation (A11b) holds since Lemma A4 ensured that the existing function *G* is a bijection. Equation (A11c) holds since the intersection is a subset of each individual zonogon.

(A11a) $$\begin{array}{cc} \forall g_{e}\in G_{e},\exists g_{o}\in G_{o},\kappa \in A,G & :\;\;Z\left(\kappa \right)+\sum_{E\in L_{e}\setminus \emptyset }Z\left(g_{e}\left(E\right)\right)=\sum_{E\in L_{e}}Z\left(g_{o}\left(G\left(E\right)\right)\right) \end{array}$$

(A11b) $$\begin{array}{cc} \forall g_{e}\in G_{e},\exists g_{o}\in G_{o},\kappa \in A & :\;\;Z\left(\kappa \right)+\sum_{E\in L_{e}\setminus \emptyset }Z\left(g_{e}\left(E\right)\right)=\sum_{O\in L_{o}}Z\left(g_{o}\left(O\right)\right) \end{array}$$

(A11c) $$\begin{array}{cc} \forall g_{e}\in G_{e},\exists g_{o}\in G_{o} & :\;\underset{\kappa \in A}{⋂}Z\left(\kappa \right)+\sum_{E\in L_{e}\setminus \emptyset }Z\left(g_{e}\left(E\right)\right)\subseteq \sum_{O\in L_{o}}Z\left(g_{o}\left(O\right)\right) \end{array}$$

Equation (A11c) is parameterized by $g_{e}$, and the subsets are closed under set union. Therefore, we can combine all choices for $g_{e}$ and $g_{o}$ using the set-theoretic union as shown below. For the notation, let $m=2^{|A|-1}$, and we indicate subsets of A with even cardinality as $E_{i}\in L_{e}$, where 1≤i≤m. We use the last index for the empty set $E_{m}=\emptyset$. The subsets of A with odd cardinality are correspondingly noted as $O_{i}\in L_{o}$. For clarity, we note binary input channels from an even subset as λ∈E and binary input channels from an odd subset as ν∈O.

$$\begin{array}{cccc} \underset{\begin{array}{c} \lambda_{1}\in E_{1}\\ \lambda_{2}\in E_{2}\\ \cdots \\ \lambda_{m-1}\in E_{m-1} \end{array}}{⋃}\left(\underset{\kappa \in A}{⋂}Z\left(\kappa \right)+\sum_{i=1}^{m-1}Z\left(\lambda_{i}\right)\right) & \subseteq \underset{\begin{array}{c} \nu_{1}\in O_{1}\\ \nu_{2}\in O_{2}\\ \cdots \\ \nu_{m}\in O_{m} \end{array}}{⋃}\left(\sum_{j=1}^{m}Z\left(\nu_{j}\right)\right) & \\ \underset{\kappa \in A}{⋂}Z\left(\kappa \right)+\sum_{i=1}^{m-1}\;\underset{\lambda \in E_{i}}{⋃}Z\left(\lambda \right) & \subseteq \sum_{j=1}^{m}\;\underset{\nu \in O_{j}}{⋃}Z\left(\nu \right) & \left(\begin{array}{c} \mathrm{Minkowski}\;\mathrm{sum}\;\mathrm{dis}\\ \mathrm{tributes}\;\mathrm{over}\;\mathrm{set}\;\mathrm{union} \end{array}\right)\\ \mathrm{Conv}\left(\underset{\kappa \in A}{⋂}Z\left(\kappa \right)+\sum_{i=1}^{m-1}\;\underset{\lambda \in E_{i}}{⋃}Z\left(\lambda \right)\right) & \subseteq \mathrm{Conv}\left(\sum_{j=1}^{m}\;\underset{\nu \in O_{j}}{⋃}Z\left(\nu \right)\right) & \left(\begin{array}{c} \mathrm{if}\;X\subseteq Y\;\mathrm{then}\\ \;\mathrm{Conv}(X)\subseteq \mathrm{Conv}(Y) \end{array}\right)\\ \underset{\kappa \in A}{⋂}Z\left(\kappa \right)+\sum_{i=1}^{m-1}\;\mathrm{Conv}\left(\underset{\lambda \in E_{i}}{⋃}Z\left(\lambda \right)\right) & \subseteq \sum_{j=1}^{m}\;\mathrm{Conv}\left(\underset{\nu \in O_{j}}{⋃}Z\left(\nu \right)\right) & \left(\begin{array}{c} \mathrm{Convex}\;\mathrm{hull}\;\mathrm{distributes}\\ \mathrm{over}\;\mathrm{Minkowski}\;\mathrm{sum} \end{array}\right) & \\ Z\left({⊓}_{\kappa \in A}\kappa \right)+\sum_{i=1}^{m-1}\;Z\left(\underset{\lambda \in E_{i}}{⨆}\lambda \right) & \subseteq \sum_{j=1}^{m}\;Z\left(\underset{\nu \in O_{j}}{⨆}\nu \right) & \left(\mathrm{by}\;\mathrm{Equation}\;(7)\right)\\ Z\left({⊓}_{\kappa \in A}\kappa \right)+\sum_{\overset{\emptyset \neq E_{i}\subseteq A}{|E_{i}|\;\mathrm{even}}}\;Z\left(\underset{\lambda \in E_{i}}{⨆}\lambda \right) & \subseteq \sum_{\overset{O_{j}\subseteq A}{|O_{j}|\;\mathrm{odd}}}\;Z\left(\underset{\nu \in O_{j}}{⨆}\nu \right) & \left(\mathrm{replace}\;\mathrm{notation}\right)\\ Z\left({⊓}_{\kappa \in A}\kappa \right)+\sum_{\overset{\emptyset \neq B\subseteq A}{|B|\;\mathrm{even}}}\;Z\left(\underset{\lambda \in B}{⨆}\lambda \right) & \subseteq \sum_{\overset{B\subseteq A}{|B|\;\mathrm{odd}}}\;Z\left(\underset{\nu \in B}{⨆}\nu \right) \end{array}$$

□

## Appendix C. Non-Negativity of Partial f-Information on the Synergy Lattice

The proof of non-negativity can be divided into three parts. First, we show that the loss measure maintains the ordering relation of the synergy lattice and how the quantification of a meet element $i_{\cup ,f}\left(\alpha \wedge \beta ,T,t\right)$ can be computed. Second, we demonstrate how the inclusion–exclusion inequality of zonogons under the Minkowski sum from Appendix B leads to relating pointwise information measures with respect to the Blackwell order. Finally, we combine these two results to demonstrate that an inclusion–exclusion relation using the convex hull of zonogons is greater than their intersection and obtain the non-negativity of the decomposition by transitivity.

### Appendix C.1. Properties of the Loss Measure on the Synergy Lattice

**Lemma A6.** 
*Any set of sources $\alpha \in P(P_{1}\left(V\right))$ is equivalent (≅) to some atom of the synergy lattice γ∈A(V).*

$$\forall \alpha \in P(P_{1}\left(V\right)).\;\exists \gamma \in A\left(V\right).\;\gamma ≅\alpha$$

*The union for two sets of sources is equivalent to the meet of their corresponding atoms on the synergy lattice. Let $\alpha ,\beta \in P(P_{1}\left(V\right))$ and γ,δ∈A(V):*

$$\begin{array}{c} \gamma ≅\alpha \;\mathrm{and}\;\delta ≅\beta ⟹(\gamma \wedge \delta )≅(\alpha \cup \beta ) \end{array}$$

**Proof.** The used filter in the definition of an atom ($A\left(V\right)\subseteq P(P_{1}\left(V\right))$, Equation (8)) only removes sets of cardinality 2≤|α|, and for any removed set of sources, we can construct an equivalent set that contains one less source by removing the subset $S_{a}\subset S_{b}$ as shown in Equation (A12a). Therefore, all sets of sources $\alpha \in P(P_{1}\left(V\right))$ are equivalent to some atom γ∈A(V) within the lattice (Equation (A12b)).

(A12a) $$\begin{array}{ccc} S_{a}\subset S_{b}⟹\alpha & ≅(\alpha \setminus S_{a}) & \mathrm{where}:\;\;S_{a},S_{b}\in \alpha \end{array}$$

(A12b) $$\begin{array}{cc} \forall \alpha \in P(P_{1}\left(V\right)),\exists \gamma \in A\left(V\right).\;\alpha & ≅\gamma \end{array}$$

The union of two sets of sources $\alpha \in P(P_{1}\left(V\right))$ is inferior to each individual set α and β:

$$\begin{array}{ccc} \;\;\;(\alpha \cup \beta ) & ⪯\alpha \;\;\; & (\mathrm{by}\;\mathrm{Equation}\;(10))\\ \left(\alpha \cup \beta \right) & ⪯\beta & (\mathrm{by}\;\mathrm{Equation}\;(10)) \end{array}$$

All sets of sources $\varepsilon \in P(P_{1}\left(V\right))$ that are inferior to both α and β (ε⪯α and ε⪯β) are also inferior to their union.

$$\begin{array}{ccc} \varepsilon ⪯\alpha \;\;\mathrm{and}\;\;\varepsilon ⪯\beta & ⟹\varepsilon ⪯(\alpha \cup \beta )\; & (\mathrm{by}\;\mathrm{Equation}\;(10)) \end{array}$$

Therefore, the union of α and β is equivalent to the meet of their corresponding atoms on the synergy lattice. □

**Proof of Lemma 1 from Section 3.2.**

For any set of sources $\alpha ,\beta \in P(P_{1}\left(V\right))$ and target variable *T* with state t∈T, the function $\kappa_{⊔}$ (Equation (31)) maintains the ordering from the synergy lattice under the Blackwell order.

(A13) $$\alpha ⪯\beta ⟹\kappa_{⊔}\left(\beta ,T,t\right)⊑\kappa_{⊔}\left(\alpha ,T,t\right)$$

**Proof.** We consider two cases for β:

1. If β=∅, then the implication holds for any α since the bottom element $\kappa_{⊔}\left(\emptyset ,T,t\right)={⊥}_{\mathrm{BW}}$ is inferior (⊑) to any other channel.
2. If β≠∅, then α is also a non-empty set since $\alpha ⪯\beta ≺{⊤}_{\mathrm{SL}}=\emptyset$.
  $$\begin{array}{ccccc} \; & \; & \alpha & ⪯\beta \\ \forall S_{b}\in \beta ,\exists S_{a}\in \alpha . & \; & S_{b} & \subseteq S_{a} & (\mathrm{by}\;\mathrm{Equation}\;(10))\\ \forall S_{b}\in \beta ,\exists S_{a}\in \alpha . & \; & \kappa (S_{b},T,t) & ⊑\kappa (S_{a},T,t) & (\mathrm{by}\;\mathrm{Equation}\;(2))\\ \; & \; & \underset{S_{b}\in \beta }{⨆}\kappa \left(S_{b},T,t\right) & ⊑\underset{S_{a}\in \alpha }{⨆}\kappa \left(S_{a},T,t\right) & \\ \; & \; & \kappa_{⊔}\left(\beta ,T,t\right) & ⊑\kappa_{⊔}\left(\alpha ,T,t\right) & \end{array}$$

Since the implication holds for both cases, the ordering is maintained. □

**Corollary A2.** 
*The defined cumulative loss measures ($i_{\cup ,f}$ of Equation (33a) and $I_{\cup ,f}$ of Equation (34)) maintain the ordering relation of the synergy lattice for any set of sources $\alpha ,\beta \in P(P_{1}\left(V\right))$ and target variable T with state t∈T:*

$$\begin{array}{cc} \alpha ⪯\beta ⟹ & \;i_{\cup ,f}\left(\alpha ,T,t\right)\leq i_{\cup ,f}\left(\beta ,T,t\right)\\ \alpha ⪯\beta ⟹ & \;I_{\cup ,f}\left(\alpha ;T\right)\leq I_{\cup ,f}\left(\beta ;T\right) \end{array}$$

**Proof.** The pointwise monotonicity of the cumulative loss measure ($\alpha ⪯\beta ⟹i_{\cup ,f}\left(\alpha ,T,t\right)\leq i_{\cup ,f}\left(\beta ,T,t\right)$) is obtained from Lemmas 1 and A2 with Equation (33a). Sine all cumulative pointwise losses $i_{\cup ,f}$ are smaller for α than β, so will be their weighted sum ($\alpha ⪯\beta ⟹I_{\cup ,f}\left(\alpha ;T\right)\leq I_{\cup ,f}\left(\beta ;T\right)$, see Equation (34)). □

**Corollary A3.** 
*The cumulative pointwise loss of the meet from two atoms is equivalent to the cumulative pointwise loss of their union for any target variable T with state t∈T:*

*$i_{\cup ,f}\left(\alpha \wedge \beta ,T,t\right)=i_{\cup ,f}\left(\alpha \cup \beta ,T,t\right)$.*

**Proof.** The result follows from Lemma A6 and Corollary A2. □

### Appendix C.2. The Non-Negativity of the Decomposition

**Lemma A7.** 
*Consider a non-empty set of of binary input channel A≠∅ and 0≤p≤1. Quantifying an inclusion–exclusion principle on the pointwise information of their Blackwell join is larger than the pointwise information of their Blackwell meet as shown in Equation (A14).*

(A14)
$$i_{f}\left(p,\underset{\kappa \in A}{⊓}\kappa \right)\leq \sum_{\emptyset \neq B\subseteq A}{\left(-1\right)}^{|B|-1}i_{f}\left(p,\underset{\kappa \in B}{⨆}\kappa \right)$$

**Proof.** 

$$\begin{array}{ccc} Z\left(\underset{\kappa \in A}{⊓}\kappa \right)+\sum_{\overset{\emptyset \neq B\subseteq A}{|B|\;\mathrm{even}}}\;Z\left(\underset{\lambda \in B}{⨆}\lambda \right) & \subseteq \sum_{\overset{B\subseteq A}{|B|\;\mathrm{odd}}}\;Z\left(\underset{\nu \in B}{⨆}\nu \right) & \left(\mathrm{by}\;\mathrm{Lemma}\;A5\right)\\ i_{f}\left(p,\underset{\kappa \in A}{⊓}\kappa \right)+\sum_{\overset{\emptyset \neq B\subseteq A}{|B|even}}\;i_{f}\left(p,\underset{\kappa \in B}{⨆}\kappa \right) & \leq \sum_{\overset{\emptyset \neq B\subseteq A}{|B|odd}}\;i_{f}\left(p,\underset{\kappa \in B}{⨆}\kappa \right) & \left(\mathrm{by}\;\mathrm{Lemma}\;A3\right)\\ i_{f}\left(p,\underset{\kappa \in A}{⊓}\kappa \right)\leq \sum_{\emptyset \neq B\subseteq A} & {\left(-1\right)}^{|B|-1}i_{f}\left(p,\underset{\kappa \in B}{⨆}\kappa \right) \end{array}$$

□

**Lemma A8** (Non-negativity on the synergy lattice). *The decomposition of f-information is non-negative on the pointwise and combined synergy lattice for any target variable T with state t∈T:*

$$\begin{array}{cc} \forall \alpha \in A(V). & \;0\leq \Delta i_{\cup ,f}\left(\alpha ,T,t\right),\\ \forall \alpha \in A(V). & \;0\leq \Delta I_{\cup ,f}\left(\alpha ;T\right). \end{array}$$

**Proof.** We show the non-negativity of pointwise partial information ($\Delta i_{\cup ,f}\left(\alpha ,T,t\right)$) in two cases. We write $\alpha^{{-}_{S}}$ to represent the cover set of α on the synergy lattice and use $p=P_{T}\left(t\right)$ as the abbreviation:

1. Let $\alpha ={⊥}_{\mathrm{SL}}=\left\{V\right\}$. The bottom element of the synergy lattice is quantified to zero (by Equation (33a), $i_{\cup ,f}\left({⊥}_{\mathrm{SL}},T,t\right)=0$), and therefore, also its partial contribution will be zero ($\Delta i_{\cup ,f}\left({⊥}_{\mathrm{SL}},T,t\right)=0$), which implies Equation (A15).
  (A15) $$\alpha ={⊥}_{\mathrm{SL}}⟹0\leq \Delta i_{\cup ,f}\left(\alpha ,T,t\right)$$
2. Let $\alpha \in A\left(V\right)\setminus \left\{{⊥}_{\mathrm{SL}}\right\}$, then its cover set is non-empty ($\alpha^{{-}_{S}}\neq \emptyset$). Additionally, we know that no atom in the cover set is the empty set ($\forall \beta \in \alpha^{{-}_{S}}.\;\beta \neq \emptyset$), since the empty atom is the top element (${⊤}_{\mathrm{SL}}=\emptyset$).
  Since it will be required later, note that the inclusion–exclusion principle of a constant is the constant itself as shown in Equation (A16) since, without the empty set, there exists one more subset of odd cardinality than with even cardinality (see Equation (A3)).
  (A16) $$i_{f}\left(p,\kappa \left(V,T,t\right)\right)=\sum_{\emptyset \neq B\subseteq \alpha^{{-}_{S}}}{\left(-1\right)}^{|B|-1}i_{f}\left(p,\kappa \left(V,T,t\right)\right)$$
  We can re-write the Möbius inverse as shown in Equation (A17), where Equation (A17b) is obtained from ([23], p. 15)).
  (A17a) $$\begin{array}{ccc} \Delta i_{\cup ,f}\left(\alpha ,T,t\right)= & \;i_{\cup ,f}\left(\alpha ,T,t\right)-\sum_{\beta \in {\dot{↓}}_{S}\alpha }\Delta i_{\cup ,f}\left(\beta ,T,t\right) & (\mathrm{by}\;\mathrm{Equation}\;(33b)) \end{array}$$
  (A17b) $$\begin{array}{ccc} = & \;i_{\cup ,f}\left(\alpha ,T,t\right)-\sum_{\emptyset \neq B\subseteq \alpha^{{-}_{S}}}{\left(-1\right)}^{|B|-1}\cdot i_{\cup ,f}\left(\underset{\beta \in B}{⋀}\beta ,T,t\right) & \;\; \end{array}$$
  (A17c) $$\begin{array}{ccc} = & \;i_{\cup ,f}\left(\alpha ,T,t\right)-\sum_{\emptyset \neq B\subseteq \alpha^{{-}_{S}}}{\left(-1\right)}^{|B|-1}\cdot i_{\cup ,f}\left(\underset{\beta \in B}{⋃}\beta ,T,t\right) & (\mathrm{by}\;\mathrm{Corollary}\;A3) \end{array}$$
  (A17d) $$\begin{array}{ccc} = & -i_{f}\left(p,\kappa_{⊔}\left(\alpha ,T,t\right)\right)+\sum_{\emptyset \neq B\subseteq \alpha^{{-}_{S}}}{\left(-1\right)}^{|B|-1}\cdot i_{f}\left(p,\kappa_{⊔}\left(\underset{\beta \in B}{⋃}\beta ,T,t\right)\right) & (\mathrm{by}\;\mathrm{Equations}\;(33a),\;(A16)) \end{array}$$
  (A17e) $$\begin{array}{ccc} = & -i_{f}\left(p,\kappa_{⊔}\left(\alpha ,T,t\right)\right)+\sum_{\emptyset \neq B\subseteq \alpha^{{-}_{S}}}{\left(-1\right)}^{|B|-1}\cdot i_{f}\left(p,\underset{S\in ({⋃}_{\beta \in B}\beta )}{⨆}\kappa \left(S,T,t\right)\right) & (\mathrm{by}\;\forall \beta \in \alpha^{{-}_{S}}.\beta \neq \emptyset ) \end{array}$$
  (A17f) $$\begin{array}{cc} = & -i_{f}\left(p,\kappa_{⊔}\left(\alpha ,T,t\right)\right)+\sum_{\emptyset \neq B\subseteq \alpha^{{-}_{S}}}{\left(-1\right)}^{|B|-1}\cdot i_{f}\left(p,\underset{\beta \in B}{⨆}\underset{S\in \beta }{⨆}\kappa \left(S,T,t\right)\right) \end{array}$$
  (A17g) $$\begin{array}{cc} = & -i_{f}\left(p,\kappa_{⊔}\left(\alpha ,T,t\right)\right)+\sum_{\emptyset \neq B\subseteq \{\kappa_{⊔}\left(\beta ,T,t\right)\;:\;\beta \in \alpha^{{-}_{S}}\}}{\left(-1\right)}^{|B|-1}\cdot i_{f}\left(p,\underset{\kappa \in B}{⨆}\kappa \right) \end{array}$$
  Consider the non-empty set of channels $D=\{\kappa_{⊔}\left(\beta ,T,t\right)\;:\;\beta \in \alpha^{{-}_{S}}\}$, then we obtain Equation (A18b) from Lemma A7.
  (A18a) $$\begin{array}{cc} i_{f}\left(p,\underset{\kappa \in \{\kappa_{⊔}\left(\beta ,T,t\right)\;:\;\beta \in \alpha^{{-}_{S}}\}}{⊓}\kappa \right) & \leq \sum_{\emptyset \neq B\subseteq \{\kappa_{⊔}\left(\beta ,T,t\right)\;:\;\beta \in \alpha^{{-}_{S}}\}}{\left(-1\right)}^{|B|-1}i_{f}\left(p,\underset{\kappa \in B}{⨆}\kappa \right) \end{array}$$
  (A18b) $$\begin{array}{cc} i_{f}\left(p,\underset{\beta \in \alpha^{{-}_{S}}}{⊓}\kappa_{⊔}\left(\beta ,T,t\right)\right) & \leq \sum_{\emptyset \neq B\subseteq \{\kappa_{⊔}\left(\beta ,T,t\right)\;:\;\beta \in \alpha^{{-}_{S}}\}}{\left(-1\right)}^{|B|-1}i_{f}\left(p,\underset{\kappa \in B}{⨆}\kappa \right) \end{array}$$
  We can construct an upper bound on $i_{f}\left(p,\kappa_{⊔}\left(\alpha ,T,t\right)\right)$ based on the cover set $\alpha^{{-}_{S}}$ as shown in Equation (A19).
  (A19a) $$\begin{array}{cccc} \; & \forall \beta \in \alpha^{{-}_{S}}.\; & \;\beta & ⪯\alpha \end{array}$$
  (A19b) $$\begin{array}{ccccc} \; & \forall \beta \in \alpha^{{-}_{S}}.\; & \;\kappa_{⊔}\left(\alpha ,T,t\right) & ⊑\kappa_{⊔}\left(\beta ,T,t\right) & (\mathrm{by}\;\mathrm{Lemma}\;1) \end{array}$$
  (A19c) $$\begin{array}{cccc} \; & \; & \;\kappa_{⊔}\left(\alpha ,T,t\right) & ⊑\underset{\beta \in \alpha^{{-}_{S}}}{⊓}\kappa_{⊔}\left(\beta ,T,t\right) \end{array}$$
  (A19d) $$\begin{array}{ccccc} \; & \; & \;i_{f}\left(p,\kappa_{⊔}\left(\alpha ,T,t\right)\right) & \leq i_{f}\left(p,\underset{\beta \in \alpha^{{-}_{S}}}{⊓}\kappa_{⊔}\left(\beta ,T,t\right)\right) & (\mathrm{by}\;\mathrm{Lemma}\;A2) \end{array}$$
  By the transitivity of Equations (A18b) and (A19d), we obtain Equation (A20).
  (A20) $$i_{f}\left(p,\kappa_{⊔}\left(\alpha ,T,t\right)\right)\leq \sum_{\emptyset \neq B\subseteq \{\kappa_{⊔}\left(\beta ,T,t\right)\;:\;\beta \in \alpha^{{-}_{S}}\}}{\left(-1\right)}^{|B|-1}i_{f}\left(p,\underset{\kappa \in B}{⨆}\kappa \right)$$
  By Equations (A17) and (A20), we obtain the non-negativity of pointwise partial information as shown in Equation (A21).
  (A21) $$\alpha \in A\left(V\right)\setminus \left\{{⊥}_{\mathrm{SL}}\right\}.\;0\leq \Delta i_{\cup ,f}\left(\alpha ,T,t\right)$$

From Equations (A15) and (A21), we obtain that pointwise partial information is non-negative for all atoms of the lattice:

(A22) $$\forall \alpha \in A\left(V\right).\;0\leq \Delta i_{\cup ,f}\left(\alpha ,T\right)$$

If all pointwise partial components are non-negative, then their expected value will also be non-negative (see Equation (35)):

(A23) $$\forall \alpha \in A\left(V\right).\;0\leq \Delta I_{\cup ,f}\left(\alpha ;T\right)$$

□

## Appendix D. Mappings between Decomposition Lattices and Their Duality

**Proof of Lemma 2 from Section 3.4**

The function Ψ(·) is a bijection on the redundancy lattice without the bottom element (*∅*) that reverses its order. Let $\alpha ,\beta \in A\left(V\right)\setminus \left\{{⊥}_{\mathrm{RL}}\right\}$:

1. Ψ(Ψ(α))≃α;
2. α≼β⟺Ψ(β)≼Ψ(α).

**Proof.** 

- Property 1: the n-ary Cartesian product (Ψ) provides all combinations of one variable from each source (Definition 28). Let γ=Ψ(α), then by Definition 11 (≃) of equivalence Ψ(γ)≃α, we have to show that both elements are inferior to each other under the redundancy order:
  - Ψ(γ)≼α: We begin by expanding the definition of the redundancy order as shown in Equation (A24) to highlight that it is sufficient to show that α⊆Ψ(γ).
    (A24) $$\alpha \subseteq \Psi \left(\gamma \right)⟹\forall S_{a}\in \alpha ,\exists S_{b}\in \Psi \left(\gamma \right),S_{b}\subseteq S_{a}⟹\Psi \left(\gamma \right)≼\alpha$$
    To show α⊆Ψ(γ), we have to demonstrate that is is possible to select one variable from each source in γ to reconstruct each source in α:
    * By definition γ=Ψ(α), each source in γ contains one variable from each source in α, and all variables from each source in α can be found in some source of γ.
    * By selecting the variable in each source of γ that originated from the same source in α, we can exactly reconstruct each source in α.
    * Therefore, α⊆Ψ(Ψ(α)), which implies Ψ(Ψ(α))≼α.
  - α≼Ψ(γ): We begin by expanding the definition of the redundancy order (Equation (9)) as shown in Equation (A25) to highlight that we have to show that all sources in Ψ(γ) are a super-set of some source in α.
    (A25) $$\alpha ≼\Psi \left(\gamma \right)⟺\forall S_{b}\in \Psi \left(\gamma \right).\;\exists S_{a}\in \alpha .\;S_{a}\subseteq S_{b}$$
    For a proof by induction, the recursive definition $\Psi^{\prime }\left(\alpha \right)$ as shown in Equation (A26) highlights the relation of interest more clearly. We use the notation S[i] to indicate the *i*-th variable in source S. That both functions are equivalent $\Psi^{\prime }\left(\alpha \right)=\Psi \left(\alpha \right)$ can directly be seen, since $\Psi^{\prime }\left(\alpha \right)$ recursively combines all possible choices of selecting one variable from each source in α, which is the definition of Ψ(α).
    (A26) $$\begin{array}{cc} \Psi^{\prime }\left(\alpha \right) & \left\{\begin{array}{cc} \left\{\emptyset \right\} & \mathrm{if}\;\alpha =\emptyset \\ \underset{i\in 1..|S|}{⋃}\left\{x\cup \left\{S\left[i\right]\right\}\;:\;x\in \Psi^{\prime }\left(\alpha \setminus \left\{S\right\}\right)\right\}\; & \mathrm{otherwise},\;\mathrm{where}\;S\in \alpha \end{array}\right. \end{array}$$
    Induction on the cardinality of α:
    * *Hypothesis:* It is impossible to choose one variable from each source in $\Psi^{\prime }\left(\alpha \right)$ without selecting all variables of some source $S_{a}\in \alpha$:
      (A27) $$\forall S_{b}\in \Psi \left(\Psi^{\prime }\left(\alpha \right)\right).\;\exists S_{a}\in \alpha .\;S_{a}\subseteq S_{b}$$
    * Base case |α|=1: The condition is satisfied as shown in Equation (A28), since $\Psi^{\prime }\left(\left\{S\right\}\right)$ turns each variable in S into its own source. The second application $\Psi (\Psi^{\prime }\left(\left\{S\right\}\right))$ recombines them.
      (A28) $$\begin{array}{cc} \Psi^{\prime }\left(\left\{S\right\}\right) & =\{\{V\}\;:\;V\in S\}\\ \Psi (\Psi^{\prime }\left(\left\{S\right\}\right)) & =\{S\} \end{array}$$
    * Assume the induction hypothesis holds for |α|=m.
    * For the induction step, let $\alpha^{\prime }=\alpha \cup \left\{S^{\prime }\right\}$: From the recursive definition shown in Equation (A29), we can directly see all relevant options of choosing one element from each resulting source.
      (A29) $$\Psi^{\prime }\left(\alpha^{\prime }\right)=\underset{i\in 1..|S^{\prime }|}{⋃}\left\{x\cup \left\{S^{\prime }\left[i\right]\right\}\;:\;x\in \Psi^{\prime }\left(\alpha \right)\right\}$$
      · Case 1: From every source in $\Psi^{\prime }\left(\alpha^{\prime }\right)$, we choose the variable $S^{\prime }\left[i\right]$ that was contributed by the new source $S^{\prime }$. The resulting set contains all variables of $S^{\prime }$.
      · Case 2: To avoid choosing all variables from $S^{\prime }$, we have to select the variables contributed by $x\in \Psi^{\prime }\left(\alpha \right)$ instead for some $S^{\prime }\left[i\right]$. By the induction hypothesis, choosing one variable from each set in $\Psi^{\prime }\left(\alpha \right)$ leads to choosing all variables of some source $S_{a}\in \alpha$.
      · Choosing one variable from each set in $\alpha^{\prime }=\alpha \cup \left\{S^{\prime }\right\}$ leads to choosing all variables of $S^{\prime }$ or all variables of some source $S_{a}\in \alpha$.
      · Thus, the induction hypothesis holds for $|\alpha^{\prime }|=|\alpha |+1$.
  - As shown above, Ψ(Ψ(α))≼α and α≼Ψ(Ψ(α)), which implies α≃Ψ(Ψ(α)).
- Property 2: We first expand the definitions:
  $$\begin{array}{ccc} \alpha ≼\beta & ⟺\Psi (\beta )≼\Psi (\alpha )\\ \forall S_{b}\in \beta .\;\exists S_{a}\in \alpha .\;S_{a}\subseteq S_{b} & ⟺\forall S_{c}\in \Psi \left(\alpha \right).\;\exists S_{d}\in \Psi \left(\beta \right).\;S_{d}\subseteq S_{c} & (\mathrm{by}\;\mathrm{Definition}\;9) \end{array}$$
  Then, we view both implications separately:
  1. Assume $\forall S_{b}\in \beta .\;\exists S_{a}\in \alpha .\;S_{a}\subseteq S_{b}$. Then, there exists a function $w:P\left(P_{1}\left(V\right)\right)\to P\left(P_{1}\left(V\right)\right)$ that associates each source in β with a source in α.
    (A30) $$\forall S_{b}\in \beta .\;w\left(S_{b}\right)\subseteq S_{b}\;\mathrm{and}\;w\left(S_{b}\right)\in \alpha .$$
    All sets $S_{c}\in \Psi \left(\alpha \right)$ contain one variable of each source in α. Let the function $v_{c}:P_{1}\left(V\right)\to V$ indicate this selection:
    (A31) $$S_{c}=\left\{v_{c}\left(S_{x}\right)\;:\;S_{x}\in \alpha \right\}\;\mathrm{where}:\;v_{c}\left(S_{x}\right)\in S_{x}$$
    Define the set $S_{d}\in \Psi \left(\beta \right)$ using the defined functions above as shown in Equation (A32). The function *w* is defined for all sources in β, and the selected element is in the original source ($v_{c}\left(w\left(S_{x}\right)\right)\in S_{x}$) by Equation (A30).
    (A32) $$S_{d}=\left\{v_{c}\left(w\left(S_{x}\right)\right)\;:\;S_{x}\in \beta \right\}$$
    The constructed set $S_{d}\in \Psi \left(\beta \right)$ is a subset of $S_{c}\in \Psi \left(\alpha \right)$, and it can be constructed for each $S_{c}\in \Psi \left(\alpha \right)$. This proves Equation (A33):
    (A33) $$\forall S_{b}\in \beta .\;\exists S_{a}\in \alpha .\;S_{a}\subseteq S_{b}⟹\forall S_{c}\in \Psi \left(\alpha \right).\;\exists S_{d}\in \Psi \left(\beta \right).\;S_{d}\subseteq S_{c}$$
  2. For the other direction, we show Equation (A34) and start with its simplification:
    (A34) $$\begin{array}{cc} \neg (\forall S_{b}\in \beta .\;\exists S_{a}\in \alpha .\;S_{a}\subseteq S_{b}) & ⟹\neg (\forall S_{c}\in \Psi \left(\alpha \right).\;\exists S_{d}\in \Psi \left(\beta \right).\;S_{d}\subseteq S_{c})\\ \exists S_{b}\in \beta .\;\forall S_{a}\in \alpha .\;\neg \left(S_{a}\subseteq S_{b}\right) & ⟹\exists S_{c}\in \Psi \left(\alpha \right).\;\forall S_{d}\in \Psi \left(\beta \right).\;\neg \left(S_{d}\subseteq S_{c}\right)\\ \exists S_{b}\in \beta .\;\forall S_{a}\in \alpha .\;\exists x\in S_{a}.\;x\notin S_{b} & ⟹\exists S_{c}\in \Psi \left(\alpha \right).\;\forall S_{d}\in \Psi \left(\beta \right).\;\exists x\in S_{d}.\;x\notin S_{c} \end{array}$$
    The left-hand side states that, for some $S_{b}\in \beta$, all sources $S_{a}\in \alpha$ contain an element that is not in $S_{b}$. Let us fix a particular $S_{b}$ and define a function returning this element $v:P_{1}\left(V\right)\to V$:
    (A35) $$\forall S_{a}\in \alpha .\;v\left(S_{a}\right)\in S_{a}\;\mathrm{and}\;v\left(S_{a}\right)\notin S_{b}$$
    Then, we can define the set $S_{c}=\left\{v\left(S_{a}\right)\;:\;S_{a}\in \alpha \right\}$. The source $S_{c}$ selects one variable from each source; thus, $S_{c}\in \Psi \left(\alpha \right)$, and by definition, $S_{c}\cap S_{b}=\emptyset$. All sets $S_{d}\in \Psi \left(\beta \right)$ must select one element from $S_{b}$ and, thus, contain one element that is not in $S_{c}$. This provides the required implication of Equation (A34).

□

**Proof of Lemma 3 from Section 3.4**

The function Ξ(·) is a bijection that maintains the ordering of atoms between the redundancy and synergy order. Let α,β∈A(V):

1. α=Ξ(Ξ(α));
2. α≼β⟺Ξ(α)⪯Ξ(β).

**Proof.** 

- Property 1 is obtained from Definition 28: the first two cases revert each other, and the third case (α≠∅ and α≠{V}) holds since ∀S∈α:S=V∖(V∖S).
- Property 2:
  - Case 1: If $\alpha =\emptyset ={⊥}_{\mathrm{RL}}$, then $\Xi \left(\alpha \right)=\left\{V\right\}={⊥}_{\mathrm{SL}}$. Therefore, $\forall \beta \in A\left(V\right).\;{⊥}_{\mathrm{RL}}≼\beta ⟺{⊥}_{\mathrm{SL}}⪯\beta$.
  - Case 2: If $\alpha =\left\{V\right\}={⊤}_{\mathrm{RL}}$, then $\Xi \left(\alpha \right)=\emptyset ={⊤}_{\mathrm{SL}}$. Therefore, $\forall \alpha \in A\left(V\right).\;\alpha ≼{⊤}_{\mathrm{RL}}⟺\alpha ⪯{⊤}_{\mathrm{SL}}$.
  - Case 3: If α≠∅, then β≠∅:
    $$\begin{array}{ccc} \alpha ≼\beta & =\forall S_{b}\in \beta ,\exists S_{a}\in \alpha ,S_{a}\subseteq S_{b} & (\mathrm{by}\;\mathrm{Definition}\;9)\\ \; & =\forall S_{b}\in \beta ,\exists S_{a}\in \alpha ,\left(V\setminus S_{b}\right)\subseteq \left(V\setminus S_{a}\right)\\ \; & =\left\{V\setminus S_{a}:S_{a}\in \alpha \right\}⪯\left\{V\setminus S_{b}:S_{b}\in \beta \right\} & (\mathrm{by}\;\mathrm{Definition}\;10)\\ \; & =\Xi (\alpha )⪯\Xi (\beta ) & (\mathrm{by}\;\mathrm{Definition}\;28) \end{array}$$

□

**Proof of Lemma 4 from Section 3.4**

A redundancy- and synergy-based information decomposition is pointwise dual if, for all $\alpha \in A\left(V\right)\setminus \left\{{⊥}_{\mathrm{RL}}\right\}$:

(A36) $$\begin{array}{cc} \Delta i_{\cap ,f}\left(\alpha ,T,t\right) & =\Delta i_{\cup ,f}\left(\Xi \left(\Psi \left(\alpha \right)\right),T,t\right)\\ \Delta i_{\cap ,f}\left({⊥}_{\mathrm{RL}},T,t\right) & =0=\Delta i_{\cup ,f}\left({⊥}_{\mathrm{SL}},T,t\right) \end{array}$$

A redundancy- and synergy-based information decomposition is dual if, for all $\alpha \in A\left(V\right)\setminus \left\{{⊥}_{\mathrm{RL}}\right\}$:

(A37) $$\begin{array}{cc} \Delta I_{\cap ,f}\left(\alpha ,T\right) & =\Delta I_{\cup ,f}\left(\Xi \left(\Psi \left(\alpha \right)\right),T\right)\\ \Delta I_{\cap ,f}\left({⊥}_{\mathrm{RL}},T\right) & =0=\Delta I_{\cup ,f}\left({⊥}_{\mathrm{SL}},T\right) \end{array}$$

**Proof.** 

$$\begin{array}{ccc} \sum_{{\beta \in ↓}_{R}\alpha }\Delta i_{\cap ,f}\left(\beta ,T,t\right) & =\sum_{\beta \in \left({↓}_{R}\alpha \right)\setminus \left\{{⊥}_{\mathrm{RL}}\right\}}\Delta i_{\cap ,f}\left(\beta ,T,t\right) & \left(\Delta i_{\cap ,f}\left({⊥}_{\mathrm{RL}},T,t\right)=i_{\cup ,f}\left({⊥}_{\mathrm{SL}},T,t\right)=0\right)\\ \; & =\sum_{{\beta \in ↑}_{S}\Xi \left(\Psi \left(\alpha \right)\right)}\Delta i_{\cap ,f}\left(\Psi \left(\Xi \left(\beta \right)\right),T,t\right) & (\mathrm{by}\;\mathrm{Corollary}\;1)\\ \; & =\sum_{{\beta \in ↑}_{S}\Xi \left(\Psi \left(\alpha \right)\right)}\Delta i_{\cup ,f}\left(\Xi \left(\Psi \left(\Psi \left(\Xi \left(\beta \right)\right)\right)\right),T,t\right) & (\mathrm{by}\;\mathrm{Equation}\;(38))\\ \; & =\sum_{{\beta \in ↑}_{S}\Xi \left(\Psi \left(\alpha \right)\right)}\Delta i_{\cup ,f}\left(\beta ,T,t\right) \end{array}$$

The duality of the pointwise measure ($i_{\cap ,f}$, $i_{\cup ,f}$) implies the duality of the combined measure ($I_{\cap ,f}$, $I_{\cup ,f}$). □

**Lemma A9.** 
*For $\alpha \in A\left(V\right)\setminus \left\{{⊥}_{\mathrm{RL}}\right\}$ and β∈A(V):*

(A38)
$$\neg \left(\exists S_{a}\in \alpha .\;\beta ⪯\left\{S_{a}\right\}\right)⟺\left(\Xi (\Psi (\alpha ))⪯\beta \right)$$

**Proof.** 

- Case $\beta ={⊤}_{\mathrm{SL}}=\emptyset$: The condition holds since it implies α to be the minimal element in $A\left(V\right)\setminus \left\{{⊥}_{\mathrm{RL}}\right\}$.
- Case $\beta \neq {⊤}_{\mathrm{SL}}$: We start by simplifying the expression.
  (A39) $$\begin{array}{ccc} \neg \left(\exists S_{a}\in \alpha .\;\beta ⪯\left\{S_{a}\right\}\right)\; & ⟺\left(\Xi (\Psi (\alpha ))⪯\beta \right)\\ \left(\forall S_{a}\in \alpha .\;\neg \left(\beta ⪯\left\{S_{a}\right\}\right)\right)\; & ⟺\left(\Xi (\Psi (\alpha ))⪯\beta \right)\\ \left(\forall S_{a}\in \alpha .\;\neg \left(\exists S_{b}\in \beta .\;S_{a}\subseteq S_{b}\right)\right) & ⟺\left(\Xi (\Psi (\alpha ))⪯\beta \right) & (\mathrm{by}\;\mathrm{Definition}\;10)\\ \left(\forall S_{a}\in \alpha .\;\forall S_{b}\in \beta .\;\neg \left(S_{a}\subseteq S_{b}\right)\right) & ⟺\left(\Xi (\Psi (\alpha ))⪯\beta \right)\\ \left(\forall S_{a}\in \alpha .\;\forall S_{b}\in \beta .\;\neg \left(S_{a}\subseteq S_{b}\right)\right) & ⟺\left(\Psi (\alpha )≼\Xi (\beta )\right) & (\mathrm{by}\;\mathrm{Lemma}\;3)\\ \left(\forall S_{a}\in \alpha .\;\forall S_{b}\in \beta .\;\neg \left(S_{a}\subseteq S_{b}\right)\right) & ⟺\left(\forall S_{b}\in \Xi \left(\beta \right).\;\exists S_{c}\in \Psi \left(\alpha \right).\;S_{c}\subseteq S_{b}\right) & (\mathrm{by}\;\mathrm{Definition}\;9)\\ \left(\forall S_{b}\in \beta .\;\forall S_{a}\in \alpha .\;\neg \left(S_{a}\subseteq S_{b}\right)\right) & ⟺\left(\forall S_{b}\in \beta .\;\exists S_{c}\in \Psi \left(\alpha \right).\;S_{c}\subseteq V\setminus S_{b}\right) & (\mathrm{by}\;\mathrm{Definition}\;28)\\ \left(\forall S_{b}\in \beta .\;\forall S_{a}\in \alpha .\;\neg \left(S_{a}\subseteq S_{b}\right)\right) & ⟺\left(\forall S_{b}\in \beta .\;\exists S_{c}\in \Psi \left(\alpha \right).\;S_{c}\cap S_{b}=\emptyset \right)\\ \left(\forall S_{b}\in \beta .\;\forall S_{a}\in \alpha .\;\exists x\in S_{a}.\;x\notin S_{b}\right) & ⟺\left(\forall S_{b}\in \beta .\;\exists S_{c}\in \Psi \left(\alpha \right).\;\forall x\in S_{c}.\;x\notin S_{b}\right) \end{array}$$
  - The left-hand side states that, for all $S_{b}\in \beta$, all $S_{a}\in \alpha$ must have at least one element that is not in $S_{b}$.
  - The right-hand side states that, for all $S_{b}\in \beta$, there exists a combination of one variable per source in α such that no element of the resulting collection is in $S_{b}$. This is possible if and only if all sources $S_{a}\in \alpha$ have at least one element that is not in $S_{b}$.
  Therefore, both statements imply each other.

□

**Lemma A10.** 
*For $\alpha \in A\left(V\right)\setminus \left\{{⊥}_{\mathrm{RL}}\right\}$:*

(A40)
$$P_{1}\left(\alpha \right)\;\left\{≅\right\}\;\left\{\underset{\beta \in B}{⋀}\beta :\;\emptyset \neq B\subseteq \left\{\left\{S\right\}\;:\;S\in \alpha \right\}\right\}$$

**Proof.** 

(A41) $$\begin{array}{ccc} \; & =P_{1}\left(\alpha \right)\\ \; & =\left\{B:\;\emptyset \neq B\subseteq \alpha \right\}\\ \; & =\left\{\underset{\beta \in B}{⋃}\beta :\;\emptyset \neq B\subseteq \left\{\left\{S\right\}\;:\;S\in \alpha \right\}\right\}\\ \; & \;\left\{≅\right\}\;\left\{\underset{\beta \in B}{⋀}\beta :\;\emptyset \neq B\subseteq \left\{\left\{S\right\}\;:\;S\in \alpha \right\}\right\} & (\mathrm{by}\;\mathrm{Lemma}\;A6) \end{array}$$

□

**Proof of Lemma 6 from Section 3.4**

The pointwise dual-decomposition for the redundancy lattice of a loss measure on the synergy lattice is defined by:

(A42) $$i_{\cap ,f}\left(\alpha ,T,t\right)\left\{\begin{array}{cc} 0 & \mathrm{if}\;\alpha =\emptyset \\ i_{\cup ,f}\left({⊤}_{\mathrm{SL}},T,t\right)-\sum_{\beta \in P_{1}\left(\alpha \right)}{\left(-1\right)}^{|\beta |-1}i_{\cup ,f}\left(\beta ,T,t\right) & \mathrm{otherwise} \end{array}\right.$$

**Proof.** The case α=∅ is satisfied by definition. Therefore, we proceed assuming α≠∅:

(A43) $$\begin{array}{ccc} i_{\cap ,f}\left(\alpha ,T,t\right)\; & =\sum_{{\gamma \in ↓}_{R}\alpha }\Delta i_{\cap ,f}\left(\gamma ,T,t\right)\\ \; & =\sum_{{\gamma \in ↑}_{S}\Xi \left(\Psi \left(\alpha \right)\right)}\Delta i_{\cup ,f}\left(\gamma ,T,t\right) & (\mathrm{by}\;\mathrm{Lemma}\;4)\\ \; & =i_{\cup ,f}\left({⊤}_{\mathrm{SL}},T,t\right)-i_{\cup ,f}\left({⊤}_{\mathrm{SL}},T,t\right)+\sum_{{\gamma \in ↑}_{S}\Xi \left(\Psi \left(\alpha \right)\right)}\Delta i_{\cup ,f}\left(\gamma ,T,t\right)\\ \; & =i_{\cup ,f}\left({⊤}_{\mathrm{SL}},T,t\right)-\left(\sum_{\gamma \in A(V)}\Delta i_{\cup ,f}\left(\gamma ,T,t\right)-\sum_{{\gamma \in ↑}_{S}\Xi \left(\Psi \left(\alpha \right)\right)}\Delta i_{\cup ,f}\left(\gamma ,T,t\right)\right)\\ \; & =i_{\cup ,f}\left({⊤}_{\mathrm{SL}},T,t\right)-\sum_{{\gamma \in A\left(V\right)\setminus ↑}_{S}\Xi \left(\Psi \left(\alpha \right)\right)}\Delta i_{\cup ,f}\left(\gamma ,T,t\right)\\ \; & =i_{\cup ,f}\left({⊤}_{\mathrm{SL}},T,t\right)-\sum_{\gamma \in {⋃}_{S_{a}\in \alpha }{↓}_{S}\left\{S_{a}\right\}}\Delta i_{\cup ,f}\left(\gamma ,T,t\right) & (\mathrm{by}\;\mathrm{Lemma}\;5)\\ \; & =i_{\cup ,f}\left({⊤}_{\mathrm{SL}},T,t\right)-\sum_{\gamma \in {⋃}_{\beta \in \{\left\{S_{a}\right\}\;:\;S_{a}\in \alpha \}}{↓}_{S}\beta }\Delta i_{\cup ,f}\left(\gamma ,T,t\right)\\ \; & =i_{\cup ,f}\left({⊤}_{\mathrm{SL}},T,t\right)-\sum_{\emptyset \neq B\subseteq \{\left\{S_{a}\right\}\;:\;S_{a}\in \alpha \}}{\left(-1\right)}^{|\beta |-1}i_{\cup ,f}\left(\underset{\beta \in B}{⋀}\beta ,T,t\right) & (\mathrm{by}\;\mathrm{inclusion}-\mathrm{exclusion})\\ \; & =i_{\cup ,f}\left({⊤}_{\mathrm{SL}},T,t\right)-\sum_{\gamma \in \left\{{⋀}_{\beta \in B}\beta :\;\emptyset \neq B\subseteq \left\{\left\{S_{a}\right\}\;:\;S_{a}\in \alpha \right\}\right\}}{\left(-1\right)}^{|\beta |-1}i_{\cup ,f}\left(\gamma ,T,t\right)\\ \; & =i_{\cup ,f}\left({⊤}_{\mathrm{SL}},T,t\right)-\sum_{\gamma \in P_{1}\left(\alpha \right)}{\left(-1\right)}^{|\beta |-1}i_{\cup ,f}\left(\gamma ,T,t\right) & (\mathrm{by}\;\mathrm{Lemma}\;A10) \end{array}$$

□

## Appendix E. Scaling f-Information Does Not Affect Its Transformation

**Lemma A11.** 
*The linear scaling of an f-information does not affect the transformation result and operator: Consider scaling an f-information measure $I_{a_{2}}\left(S;T\right)=k\cdot I_{a_{1}}\left(S;T\right)$ with k∈(0,∞), then their decomposition transformation to another measure $I_{b}\left(S;T\right)$ will be equivalent.*

**Proof.** Based on the definitions of Section 3.2, the loss measures scale linearly with the scaling of their *f*-divergence. Therefore, we obtain two cumulative loss measures, where $I_{\cup ,a_{1}}$ and $I_{\cup ,a_{2}}$ are a linear scaling of each other (Equation (A44a)). They can be transformed into another measure $I_{\cup ,b}$, as shown in Equation (A44b).

(A44a) $$\begin{array}{cc} I_{\cup ,a_{2}}\left(\alpha ;T\right) & =k\cdot I_{\cup ,a_{1}}\left(\alpha ;T\right) \end{array}$$

(A44b) $$\begin{array}{cc} I_{\cup ,b}\left(\alpha ;T\right) & =v_{1}\left(I_{\cup ,a_{1}}\left(\alpha ;T\right)\right)=v_{2}\left(I_{\cup ,a_{2}}\left(\alpha ;T\right)\right) \end{array}$$

Equation (A44b) already demonstrates that their transformation results will be equivalent and that $v_{1}\left(z\right)=v_{2}\left(k\cdot z\right)$ and $k\cdot v_{1}^{-1}\left(z\right)=v_{2}^{-1}\left(z\right)$. Therefore, their operators will also be equivalent as shown below:

x ±2 y := v2 (v2−1(x) ± v2−1(y))x ±1 y := v1 (v1−1(x) ± v1−1(y))= v2 (kv1−1(x) ± kv1−1(y))= v2 (v2−1(x) ± v2−1(y))= x ±2 y

□

## Appendix F. Decomposition Example Distributions

The probability distributions used in Figure 9 can be found in Table A1. For providing an intuition of the decomposition result for $I_{\cup ,\mathrm{TV}}$ in the generic example, we visualize its corresponding zonogons in Figure A2. It can be seen that the maximal zonogon height is obtained from $V_{1}$ (blue), which equals the maximal zonogon height of their joint distribution $\left(V_{1},V_{2}\right)$ (red). Therefore, $I_{\cup ,\mathrm{TV}}$ does not attribute partial information uniquely to $V_{2}$ or their synergy by Lemma 8.

**Table A1:** The distributions used from [13] and the generic example from [20]. The example names are abbreviations for: XOR-gate (XOR), Unique (Unq), Pointwise Unique (PwUnq), Redundant-Error (RdnErr), Two-Bit-copy (Tbc), and the AND-gate (AND) [13].

State			Probability						
$V_{1}$	$V_{2}$	T	**XOR**	**Unq**	**PwUnq**	**RdnErr**	**Tbc**	**AND**	**Generic**
0	0	0	1/4	1/4	0	3/8	1/4	1/4	0.0625
0	0	1	-	-	-	-	-	-	0.3000
0	1	0	-	1/4	1/4	1/8	-	1/4	0.1875
0	1	1	1/4	-	-	-	1/4	-	0.1500
0	2	1	-	-	1/4	-	-	-	-
1	0	0	-	-	1/4	-	-	1/4	0.0375
1	0	1	1/4	1/4	-	1/8	-	-	0.0500
1	0	2	-	-	-	-	1/4	-	-
1	1	0	1/4	-	-	-	-	-	0.2125
1	1	1	-	1/4	-	3/8	-	1/4	-
1	1	3	-	-	-	-	1/4	-	-
2	0	1	-	-	1/4	-	-	-	-

## Appendix G. The Relation of Total Variation to the Zonogon Height

**Proof of Lemma 8(a) from Section 4.1.2**

The pointwise total variation ($i_{\mathrm{TV}}$) is a linear scaling of the maximal (Euclidean) height $h^{*}$ that the corresponding zonogon Z(κ) reaches above the diagonal, as visualized in Figure 10 for any 0≤p≤1.

$$i_{\mathrm{TV}}\left(p,\kappa \right)=\frac{1-p}{2}\sum_{v\in \kappa }\left|v_{x}-v_{y}\right|=\left(1-p\right)\frac{h^{*}}{\sqrt{2}}$$

**Proof.** The point of maximal height $P^{*}$ that a zonogon Z(κ) reaches above the diagonal is visualized in Figure 10 and can be obtained as shown in Equation (A45), where $\Delta \vec{v}$ represents the slope of vector $\vec{v}$.

(A45) $$P^{*}=\sum_{\vec{v}\in \left\{\vec{v}\in \kappa \;:\;\Delta \vec{v}>1\right\}}\vec{v}$$

The maximal height (Euclidean distance) above the diagonal is calculated as shown in Equation (A46), where $P^{*}=\left(P_{x}^{*},P_{y}^{*}\right)$.

(A46) $$h^{*}=\frac{1}{2}\;{∥\left(\begin{array}{c} P_{x}^{*}-P_{y}^{*}\\ P_{y}^{*}-P_{x}^{*} \end{array}\right)∥}_{2}=\sqrt{{\left(P_{x}^{*}-P_{y}^{*}\right)}^{2}+{\left(P_{y}^{*}-P_{x}^{*}\right)}^{2}}=\sqrt{2}\left(P_{y}^{*}-P_{x}^{*}\right)$$

The pointwise total variation $i_{\mathrm{TV}}$ can be expressed as the invertible transformation of the maximal euclidean zonogon height above the diagonal as shown below, where $\vec{v}=\left({\vec{v}}_{x},{\vec{v}}_{y}\right)$.

$$\begin{array}{ccc} i_{\mathrm{TV}}\left(p,\kappa \right) & =\sum_{\vec{v}\in \kappa }\frac{1}{2}\left|\frac{{\vec{v}}_{x}}{p{\vec{v}}_{x}+\left(1-p\right){\vec{v}}_{y}}-1\right|\left(p{\vec{v}}_{x}+\left(1-p\right){\vec{v}}_{y}\right)\\ \; & =\frac{1-p}{2}\sum_{\vec{v}\in \kappa }\left|{\vec{v}}_{x}-{\vec{v}}_{y}\right|\\ \; & =\frac{1-p}{2}\left(\sum_{\vec{v}\in \left\{\vec{v}\in \kappa \;:\;\Delta \vec{v}>1\right\}}\left({\vec{v}}_{y}-{\vec{v}}_{x}\right)+\sum_{\vec{v}\in \left\{\vec{v}\in \kappa \;:\;\Delta \vec{v}\leq 1\right\}}\left({\vec{v}}_{x}-{\vec{v}}_{y}\right)\right)\\ \; & =\frac{1-p}{2}\left(\left(P_{y}^{*}-P_{x}^{*}\right)+\left(\left(1-P_{x}^{*}\right)-\left(1-P_{y}^{*}\right)\right)\right) & (\mathrm{by}\;\mathrm{Equation}\;(A45))\\ \; & =\left(1-p\right)\left(P_{y}^{*}-P_{x}^{*}\right)\\ & =\left(1-p\right)\frac{h^{*}}{\sqrt{2}} & (\mathrm{by}\;\mathrm{Equation}\;(A46)) \end{array}$$

□

**Proof of Lemma 8(b) from Section 4.1.2**

For a non-empty set of pointwise channel A and 0≤p≤1, pointwise total variation $i_{\mathrm{TV}}$ quantifies the join element to the maximum of its individual channels:

$$i_{\mathrm{TV}}\left(p,\underset{\kappa \in A}{⨆}\kappa \right)=\max_{\kappa \in A}\;i_{\mathrm{TV}}\left(p,\kappa \right)$$

**Proof.** The join element $Z({⨆}_{\kappa \in A}\kappa )$ corresponds to the convex hull of all individual zonogons (see Equation (7)). The maximal height that the convex hull reaches above the diagonal is equal to the maximum of the maximal height that each individual zonogon reaches. Since pointwise total variation is a linear scaling of the (Euclidean) zonogon height above the diagonal (Lemma 8(a) shown above), the join element is valuated to the maximum of its individual channels. □

## Appendix H. Information Flow Example Parameters and Visualization

The parameters for the Markov chain used in Section 4.2 are shown in Equation (A47), where $M_{n}=\left(X_{n},Y_{n}\right)$, $X_{i}=\left\{0,1,2\right\}$, $Y_{i}=\left\{0,1\right\}$, $P_{M_{1}}$ is the initial distribution, and $P_{M_{n+1}|M_{n}}$ is the transition matrix. The visualized results for the information flow of KL-, TV-, and $\chi^{2}$-information can be found in Figure 11, and the visualized results of $H^{2}$-, LC-, and JS-information in Figure A3.

(A47a)
$$\begin{array}{cc} \; & \begin{array}{cc} \mathrm{States}\;(X_{1},Y_{1}):\; & \;\;\;(0,0)\;\;\;(0,1)\;\;\;(1,0)\;\;\;(1,1)\;\;\;(2,0)\;\;\;(2,1)\\ P_{M_{1}}= & \;\;\left[\begin{array}{cccccc} 0.01\; & 0.81\; & 0.00\; & 0.02\; & 0.09\; & 0.07 \end{array}\right] \end{array} \end{array}$$

(A47b)
$$\begin{array}{cc} \; & \;\;\;P_{M_{n+1}|M_{n}}=\left[\begin{array}{cccccc} 0.05\;\; & 0.01\;\; & 0.04\;\; & 0.82\;\; & 0.02\;\; & 0.06\\ 0.05 & 0.82 & 0.00 & 0.01 & 0.06 & 0.06\\ 0.04 & 0.01 & 0.82 & 0.05 & 0.04 & 0.04\\ 0.03 & 0.84 & 0.02 & 0.06 & 0.04 & 0.01\\ 0.04 & 0.03 & 0.03 & 0.02 & 0.06 & 0.82\\ 0.07 & 0.04 & 0.01 & 0.03 & 0.81 & 0.04 \end{array}\right] \end{array}$$
